# Ring Enlargements of in Situ-Formed Cyclopropanones by Sulfoxonium Ylides: One-Pot Synthesis of Alkylidene Cyclobutanones

**DOI:** 10.1021/acs.joc.5c03024

**Published:** 2026-02-16

**Authors:** Ishika Agrawal, Pedram Kalvani, Daniel B. Werz

**Affiliations:** 9174 Albert-Ludwigs-Universität Freiburg, Institute of Organic Chemistry, Albertstr. 21, Freiburg 79104, Germany

## Abstract

A one-pot method for the stereoselective synthesis of alkylidenecyclobutanones from cyclopropanone surrogates is reported. Reaction partners are stable sulfoxonium ylides, leading to the four-membered rings in up to 84% yield. Mechanistic studies indicate that the transformation proceeds via nucleophilic attack of the sulfoxonium ylide on the three-membered ring, followed by ring enlargement. The stereochemical outcome of the ring expansion is substituent-dependent: alkyl groups promote complete retention of configuration, whereas aryl groups result in partial erosion of enantiopurity.

## Graphical abstract

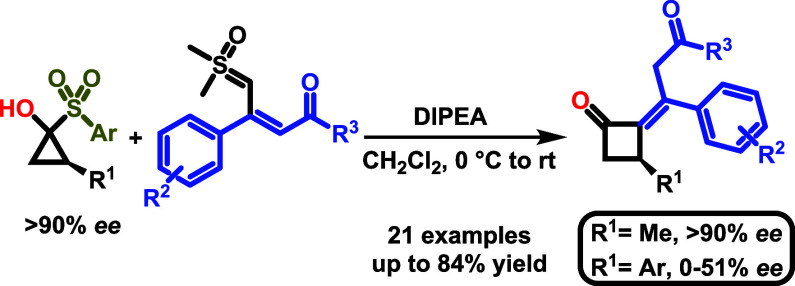

## Introduction

Cyclobutanone derivatives are highly valuable synthetic intermediates, as their inherent ring strain can be exploited in a range of strain-releasing transformations, enabling rapid access to structurally diverse scaffolds.^1^ Moreover, both cyclobutanones and cyclobutanes represent core motifs in numerous bioactive natural products and pharmaceuticals.^2–4^ Among them, alkylidene cyclobutanones have emerged as versatile building blocks, participating in stereospecific ring-opening and rearrangement reactions.^5a,5b^ Furthermore, they serve as divergent substrates to access functionalized cyclobutanones through conjugate additions of heteronucleophiles,^6a,6b^ conjugate reductions,^7a,7b^ and hydroformylations.^8^ They also display regioselective bond cleavage under photoinduced or thermal activation^9^ and can be employed as unique precursors of oxatetramethyleneethane intermediates under photoinduced electron transfer.^10a,10b^ Racemic alkylidene cyclobutanones can be prepared through several established routes, such as [2 + 2] cycloadditions between ketenes and allenes,^11a–11c^ metal-catalyzed cyclization of methylenecyclopropanes,^12^ oxidation or halohydroxylation of alkylidene cyclopropanes,^13a–13c^ ring expansion and rearrangements of 1-alkynylcyclopropanols.^14a–14c^ While these approaches efficiently deliver racemic scaffolds, stereoselective access to alkylidene cyclobutanones remains comparatively underdeveloped. Recent advances in asymmetric catalysis and diastereoselective strategies, however, have begun to provide promising solutions to this challenge.^15^

In 2021, Lindsay^16^ and coworkers reported how cyclopropanones, generated in situ from stable precursors, can serve as versatile three-carbon building blocks for the synthesis of enantioenriched alkylidenecyclobutanones. Their approach involves the addition of alkenyl-Grignard reagents to furnish alkenylcyclopropanol intermediates, which undergo *N*-bromosuccinimide-mediated electrophilic activation, followed by stereospecific 1,2-migration and elimination to deliver the target products (Scheme 1a).^16b^ However, this method requires isolation of the cyclopropanol intermediate prior to its subsequent transformation into the ring-expanded target molecule. More recently, our group developed a one-pot strategy that eliminates the need for intermediate isolation by employing nucleophilic partners capable of directly engaging the electrophilic carbonyl.^17^

**Scheme 1:**
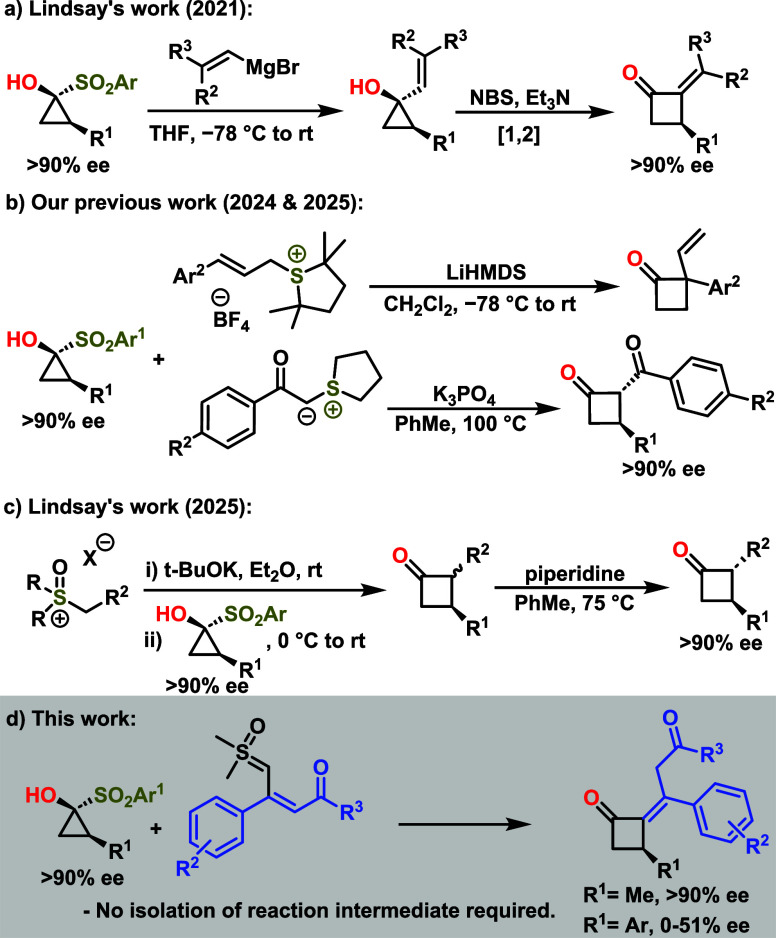
Overview of Previous Work and This Work

This protocol utilizes sulfonium ylides to access cyclobutanones by the ring-expansion of 1-sulfonylcyclopropanols (SCPs), in a one pot fashion (Scheme 1b). In parallel, Lindsay has also disclosed an alternative approach employing nonstabilized sulfoxonium ylides to furnish enantioenriched 2,3-disubstituted cyclobutanones (Scheme 1c).^16h^ Building on this strategy, we present a one-step, metal-free, and stereoselective approach to access alkylidenecyclobutanones from SCPs and stabilized sulfoxonium ylides (Scheme 1d).

## Results and Discussion

We initiated our investigations by examining the reaction of literature-known sulfoxonium ylide **2** ^18^ with SCP **1** ^16a^ as model substrate (Table 1). Following a thorough optimization of the reaction conditions (see Supporting Information for detailed information), we found that strong bases such as LiHMDS, KHMDS, and KOH (entries 1–3) led to either traces or only poor yields of product **3aa**. Major product in these cases arises from the nucleophilic substitution of sulfoxide moiety by the sulfinate anion generated in the cyclopropanone formation. However, when the reaction mixture was treated with NEt_3_ and DIPEA at 0 °C to room temperature, the desired product was observed in 64% and 80% NMR yield, respectively, (entries 4–5). By switching DCM to other solvents, the yield decreased (entries 6–9). Increasing the amount of base had an important effect on the reaction; the desired product was observed in 95% NMR yield, by using 3.4 equiv of DIPEA (entry 10). Variations in temperature and concentration led to the formation of cyclobutanone in lower yield (entries 11–12). Under the standard conditions, the desired product **3aa** was obtained in 84% isolated yield with high diastereoselectivity, predominantly as the *E* isomer (10:1 *E*:*Z* ratio), as confirmed by NMR spectroscopy and single-crystal X-ray diffraction (Scheme 2). Notably, the partial isomerization of the minor isomer (*Z*) occurred during chromatographic purification. Also, a scale-up reaction of SCP **1a** and sulfoxonium ylide **2a** to 1.0 mmol was performed, furnishing cyclobutanone **3aa** in 80% yield in a 10:1 *E:Z* ratio.

**Table 1:**
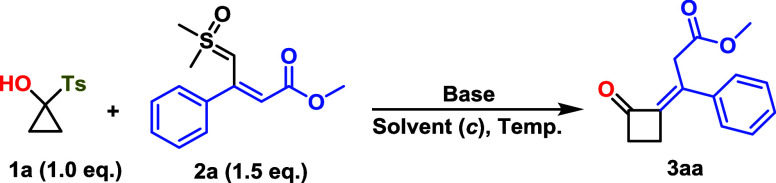
Optimization of Reaction Conditions^a^

Entry^b^	Solvent (*c*/M)^c^	Base (eq )	*T* [°C]	Yield^d^
**1**	CH_2_Cl_2_ (0.05)	LiHMDS (1.5)	–78 to rt	traces
**2**	CH_2_Cl_2_ (0.05)	KHMDS (1.5)	–78 to rt	traces
**3**	CH_2_Cl_2_ (0.05)	KOH (1.5)	0 to rt	37
**4**	CH_2_Cl_2_ (0.05)	NEt_3_(1.5)	0 to rt	64
**5**	CH_2_Cl_2_ (0.05)	DIPEA (1.5)	0 to rt	80
**6**	CHCl_3_ (0.05)	DIPEA (1.5)	0 to rt	79
**7**	DCE (0.05)	DIPEA (1.5)	0 to rt	77
**8**	THF (0.05)	DIPEA (1.5)	0 to rt	46
**9**	PhMe (0.05)	DIPEA (1.5)	0 to rt	61
**10**	CH_2_Cl_2_ (0.05)	DIPEA (3.4)	0 to rt	95^e^
**11**	CH_2_Cl_2_ (0.05)	DIPEA (3.4)	rt	83
**12**	CH_2_Cl_2_ (0.03)	DIPEA (3.4)	0 to rt	80

a See SI for detailed information.

b Reactions were carried out on a 0.1 mmol scale with respect to SCP **1**.

c Concentration with respect to SCP **1**.

d Yields refer to ^1^H NMR yields against a 1,3,5-trimethoxybenzene as standard.

e Yield of isolated and purified product is 84%.

**Scheme 2:**
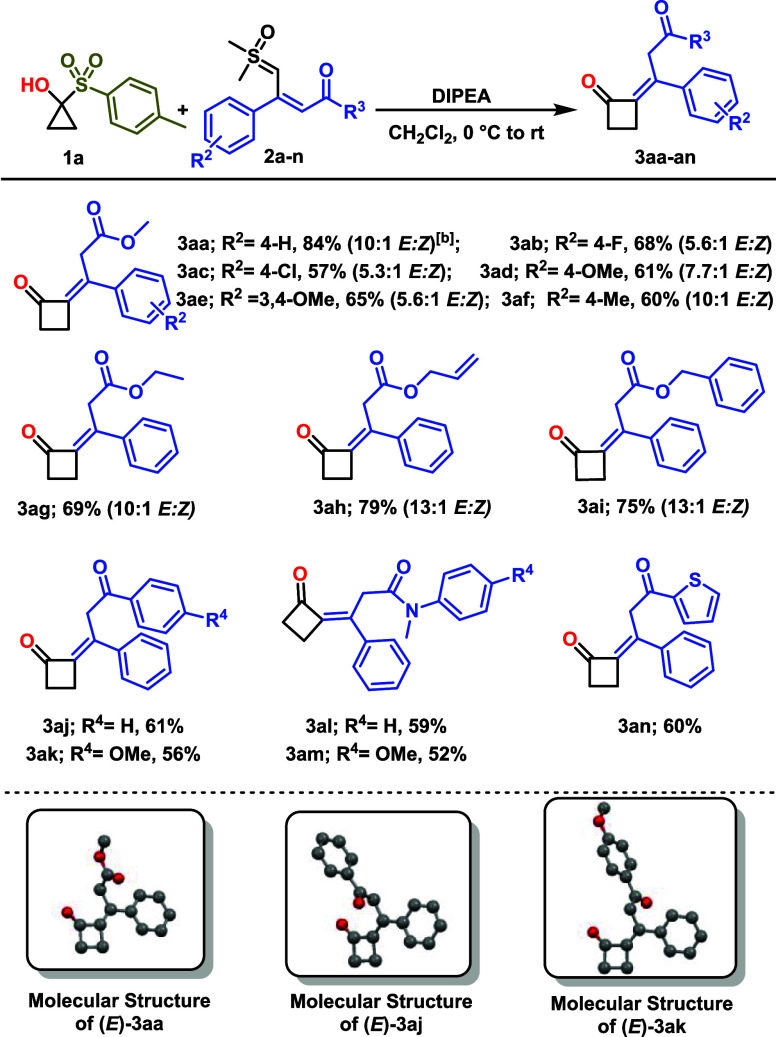
Cyclobutanone Substrate Scope with Respect to Ylides^a^ ^b^ ^c^

a Isolated yields on a 0.1 mmol scale if not stated otherwise.
b 1.0 mmol scale (**3aa** 80% yield; 10:1 *E:Z*).
c Molecular structures shown were obtained from single crystal X-ray diffraction data.

With the optimized reaction conditions in hand, we proceeded to investigate the substrate scope of the reaction. Substrate variation revealed that SCP **1a** reacted smoothly with sulfoxonium ylides^18^ **2b**–**2f** bearing electron-withdrawing and electron-donating substituents on aryl units, including F, Cl, OMe, and Me. The corresponding cyclobutanones **3ab**–**3af** were isolated in good yields (57–68%) with high diastereoselectivity. The ethyl ester-derived sulfoxonium ylide **2g** is also compatible under the optimized condition, affording **3ag** in 69% yield with a 10:1 *E*:*Z* ratio. Furthermore, allylic (**2h**) and benzylic ester-derived (**2i**) ylides exhibited high diastereoselectivity, furnishing **3ah** and **3ai** in 79% and 75% yield, respectively, with up to 13:1 *E*:*Z* selectivity. Interestingly, with introduction of keto-substituted ylides, **3aj** and **3ak** were obtained in good yields as a single diastereomer without detectable stereoisomeric mixtures. The reasons for this higher selectivity are not obvious. The structure of cyclobutanones **3aj** and **3ak** were confirmed via single-crystal X-ray diffraction (see Supporting Information for further information). Likewise, the use of amide-derived ylides **2l** and **2m** resulted in corresponding cyclobutanones **3al** and **3am** in 59% and 52% yield, respectively, both as single diastereomers. When thiophene-containing keto sulfoxonium ylide was subjected to the reaction conditions, the formation of single diastereomer **3an** was observed in 60% yield.

Next, we investigated the reactivity of chiral enantiomerically pure alkyl- and aryl-substituted SCPs **1b**–**1g** (Scheme 3). Reaction of the methyl-substituted SCP **1b** with sulfoxonium ylide **2a** afforded cyclobutanone **3ba** in 59% yield with complete retention of configuration (vide infra). The stereochemistry at the methyl-substituted carbon was proved by single-crystal X-ray crystallography of single crystals of **3ba** (see Supporting Information for further information). Phenyl-substituted SCPs **1c**–**1g** were also well tolerated, giving the corresponding cyclobutanones **3ca**–**3ga** in moderate to good yields. In addition, fused SCP **1h** exhibited smooth reactivity with **2a**, affording **3ha** in 41% yield. In all cases, the products were obtained as mixtures of *E* and *Z* isomers, with the *E* isomer as the predominant one.

**Scheme 3:**
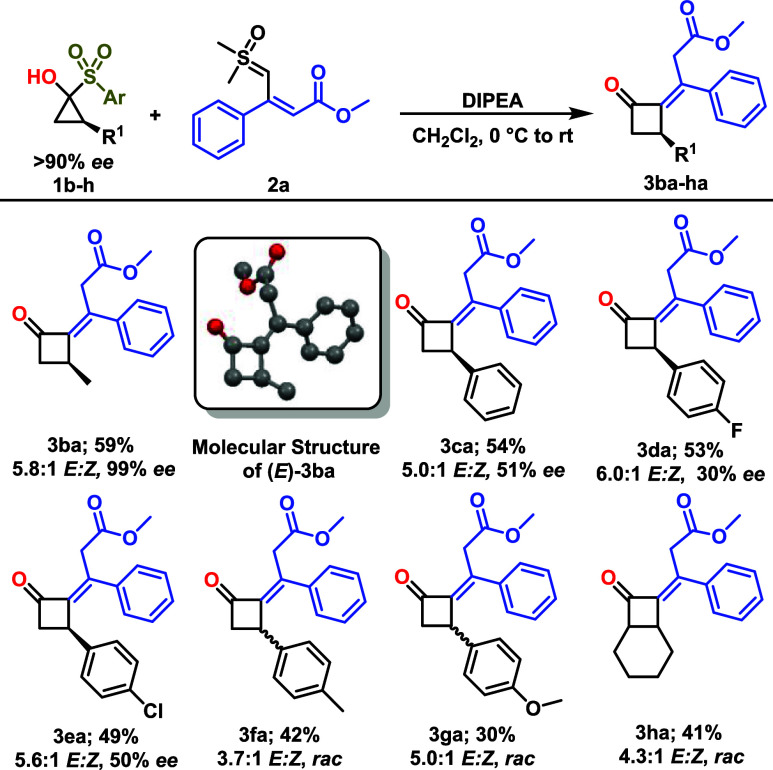
Cyclobutanone Substrate Scope with Respect to Cyclopropanones^a^ ^b^

a Isolated yields on a 0.1 mmol scale.
b Molecular structures shown were obtained from single crystal X-ray diffraction data.

HPLC analysis revealed that the reaction of Me-substituted SCP **1b** (>99% *ee*) with sulfoxonium ylide **2a**, gave **3ba** maintaining complete enantiomer purity (>99% *ee*). In contrast, enantioenriched phenyl-substituted SCP **1c** (>95% *ee*) afforded **3ca** in 51% *ee*, indicating only partial retention of enantiomer purity. These results highlight the distinct influence of different substituents on the stereospecificity of the transformation. On the basis of these observations, a plausible reaction mechanism is proposed (Scheme 4).

**Scheme 4:**
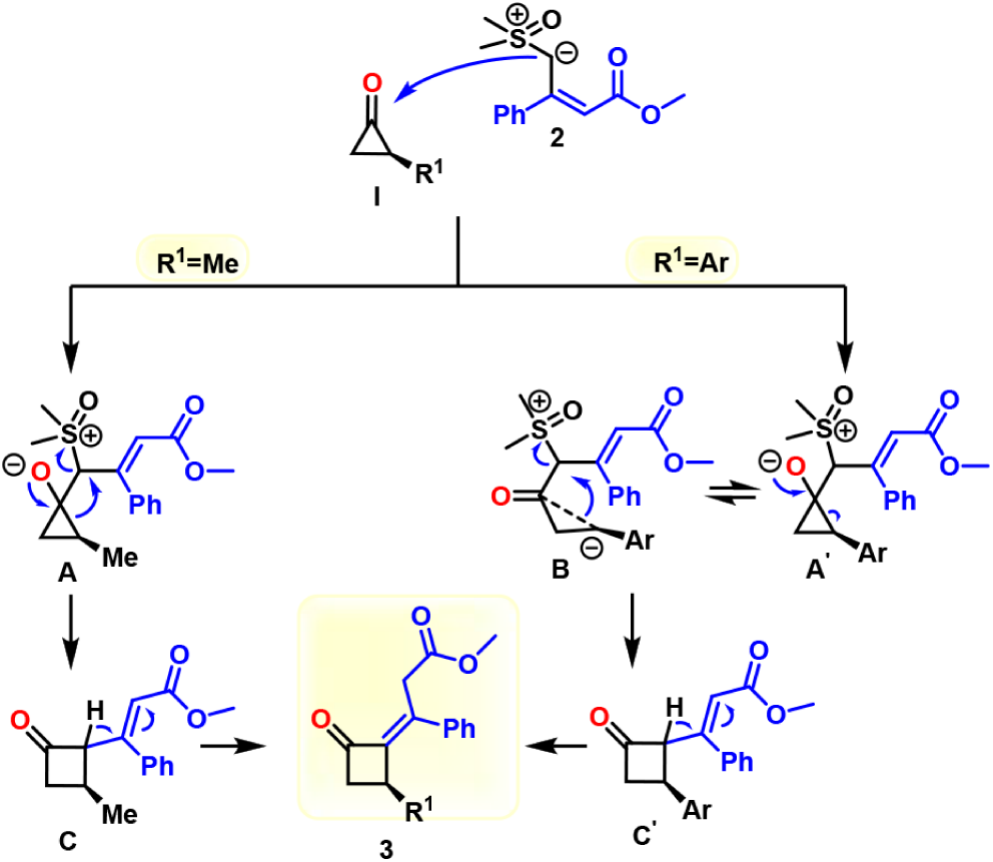
Proposed Mechanism

Under basic conditions, SCP **1** generates cyclopropanone **I**, which undergoes nucleophilic attack by sulfoxonium ylide **2** to form cyclopropoxide intermediates **A** or **A** ^′^, respectively. For aryl-substituted substrates, **A** ^′^ preferentially undergoes ring-opening to form intermediate **B**. Subsequent elimination of the sulfoxide moiety promotes ring-expansion, furnishing intermediate **C** or **C**′. Then, deprotonation followed by protonation delivers the alkylidene cyclobutanone **3**.

The greater tendency of aryl-substituted intermediates to undergo ring-opening likely accounts for the observed erosion of enantiomer purity, whereas the alkyl-substituted analogs retain their configuration through a more concerted transformation pathway. Although the mechanism only requires one equivalent of base, an excess has shown to be beneficial. We ascribe this fact either to the high sensitivity of the sulfoxonium ylide toward protonation and/or the fact that also catalytic amount of base is required for the isomerization from C/C′ to **3**.

Finally, we showed some follow-up reactions of these cyclobutanone derivatives. Alkylidene cyclobutanone **3aa** was subjected to reduction with Luche’s reagent, which selectively reduced the α,β-unsaturated carbonyl functionality to furnish corresponding allylic alcohol **4** in 52% isolated yield. Furthermore, treatment of **3aa** with methyl magnesium bromide, resulted in nucleophilic addition of the methyl group to both the cyclobutanone and ester carbonyl functionalities, affording the corresponding tertiary alcohol **5** in 49% yield. Interestingly, under excess *m*-CPBA, the reaction proceeded by epoxidation followed by ring enlargement leading to spiro-γ-lactone **6** in 50% yield as a single diastereomer, as confirmed by NOESY analysis (Scheme 5).

**Scheme 5:**
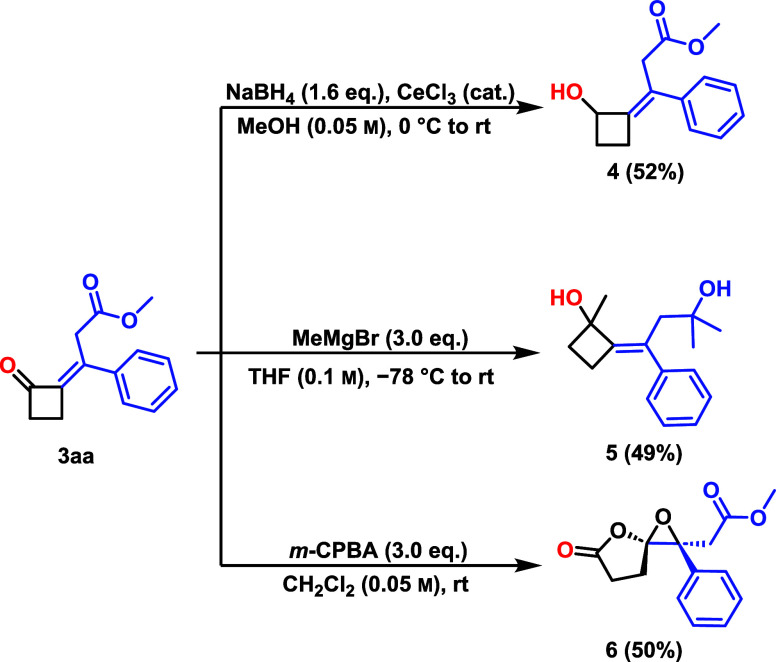
Follow-up Reactions^a^

a All reactions were performed on a 0.1 mmol scale with a single diastereomer of **3aa**.

## Conclusions

In conclusion, we have developed a one-pot method for the synthesis of diverse alkylidene cyclobutanones. This protocol employs unsubstituted, alkyl- or aryl-substituted 1-sulfonylcyclopropanols and stable sulfoxonium ylides as starting materials. A broad functional group tolerance including ester, keto, amide, and thio substituents is demonstrated. The tetrasubstituted C–C double bond is obtained in high diastereoselectivity. Depending on the type of substituent the ring enlargement from the three-to the four-membered ring either takes place with complete retention of configuration (alkyl substituent) or erosion of enantiopurity is observed (aryl substituent).

## Experimental Section

### General Procedures (GP1) for the Synthesis of Sulfoxonium Ylides **2**

Sulfoxonium ylides (**2a**–**2n**) were synthesized using a modified procedure reported by Vaitla and coworkers.^18^ An oven-dried, N_2_-purged 25 mL round-bottomed flask equipped with a magnetic stir bar was charged with NaH (60% in mineral oil, 144 mg, 3.60 mmol, 1.80 equiv) and DMSO (4.0 mL, 0.5 M). Trimethylsulfoxonium iodide (660 mg, 3.00 mmol, 1.50 eq.; recrystallized from water and dried at 70 °C under vacuum overnight) was added to the turbid solution at rt. The resulting suspension is stirred for 1 h at rt to generate the methylide solution.

Separately, oven-dried, N_2_-purged 50 mL round-bottom flask equipped with a stir bar was charged with alkyne (2.0 mmol, 1.0 equiv) dissolved in THF (5.0 mL, 0.4 M) and cooled to 0 °C. The premade methylide solution is added via cannula or syringe dropwise over 5 min. The resulting mixture was stirred 2 h at rt, then poured into crushed ice with vigorous stirring. The precipitate was collected by filtration and washed with cold ethanol/*n*-hexane (1:1) to give almost pure vinyl sulfoxonium ylides.

All analytical data for **2a**–**2k** were consistent with those reported in the literature.^18^

#### (*Z*)-4-(Dimethyl­(oxo)-λ^6^-sulfaneylidene)-*N*-methyl-*N*,3-diphenylbut-2-enamide (**2l**)

Following the **GP1**, reaction of *N*-methyl-*N*,3-diphenylprop-2-ynamide (471 mg, 2.00 mmol, 1.00 equiv) in THF (5.0 mL, 0.4 M) at 0 °C with dimethylsulfoxonium methylide generated from trimethylsulfoxonium iodide (660 mg, 3.00 mmol, 1.50 equiv) and NaH (60% in mineral oil, 144 mg, 3.60 mmol, 1.80 equiv) in DMSO (4.0 mL, 0.5 M) yielded sulfoxonium ylide **2l** as a yellow sticky solid (428 mg, 1.30 mmol, 65%). FTIR (ATR): *ṽ* [cm^–1^] = 2912, 1588, 1512, 1493, 1488, 1450, 1378, 1337, 1321, 1299, 1151, 1127, 1103, 1057, 1023, 941. ^1^H NMR (400 MHz, CDCl_3_): δ_H_ (ppm) = 7.41–7.36 (m, 2H), 7.32–7.27 (m, 5H), 7.22–7.14 (m, 3H), 6.60 (s, 1H), 4.63 (s, 1H), 3.31 (s, 3H), 2.93 (s, 6H). ^13^C­{^1^H}-NMR (101 MHz, CDCl_3_): δ_C_ (ppm) = 168.9, 154.5, 145.5, 142.0, 129.3, 129.3, 128.4, 128.2, 127.5, 126.5, 98.5, 68.6, 44.6, 37.2. HRMS (ESI, Orbitrap): calc. for C_19_H_22_O_2_NS [M + H]^+^: 328.1366, found: 328.1366.

#### (*Z*)-4-(Dimethyl­(oxo)-λ^6^-sulfaneylidene)-*N*-(4-methoxyphenyl)-*N*-methyl-3-phenylbut-2-enamide (**2m**)

Following the **GP1**, reaction of *N*-(4-methoxyphenyl)-*N*-methyl-3-phenylprop-2-ynamide (531 mg, 2.00 mmol, 1.00 equiv) in THF (5.0 mL, 0.4 M) at 0 °C with dimethylsulfoxonium methylide generated from trimethylsulfoxonium iodide (660 mg, 3.00 mmol, 1.50 equiv) and NaH (60% in mineral oil, 144 mg, 3.60 mmol, 1.80 equiv) in DMSO (4.0 mL, 0.5 M) yielded sulfoxonium ylide **2m** as a yellow solid (669 mg, 1.90 mmol, 94%). M.P. = 111–112 °C. FTIR (ATR): *ṽ* [cm^–1^] = 2914, 1599, 1508, 1488, 1461, 1433, 1338, 1325, 1302, 1245, 1149, 1130, 1104, 1058, 1024, 968. ^1^H NMR (300 MHz, CDCl_3_): δ_H_ (ppm) = 7.42–7.35 (m, 2H), 7.29 (q, *J* = 3.7 Hz, 3H), 7.14–7.06 (m, 2H), 6.85–6.79 (m, 2H), 6.60 (s, 1H), 4.61 (s, 1H), 3.76 (s, 3H), 3.27 (s, 3H), 2.93 (s, 6H). ^13^C­{^1^H}-NMR (176 MHz, CDCl_3_): δ_C_ (ppm) = 169.2, 158.1, 154.3, 142.1, 138.3, 129.4, 128.7, 128.3, 128.2, 114.6, 98.6, 68.3, 55.5, 44.7, 37.3. HRMS (ESI, Orbitrap): calc. for C_20_H_24_O_3_NS [M + H]^+^: 358.1471, found: 358.1472.

#### (*Z*)-4-(Dimethyl­(oxo)-λ^6^-sulfaneylidene)-3-phenyl-1-(thiophen-2-yl)­but-2-en-1-one (**2n**)

Following the **GP1**, reaction of 3-phenyl-1-thiophen-2-ylprop-2-yn-1-one (425 mg, 2.00 mmol, 1.00 equiv) in THF (5.0 mL, 0.4 M) at 0 °C with dimethylsulfoxonium methylide generated from trimethylsulfoxonium iodide (660 mg, 3.00 mmol, 1.50 equiv) and NaH (60% in mineral oil, 144 mg, 3.60 mmol, 1.80 equiv) in DMSO (4.0 mL, 0.5 M) yielded sulfoxonium ylide **2n** as a yellow solid (395 mg, 1.30 mmol, 65%). M.P. = 149–150 °C. FTIR (ATR): *ṽ* [cm^–1^] = 2987, 1545, 1515, 1504, 1469, 1450, 1429, 1349, 1300, 1229, 1157, 1116, 1022, 989. ^1^H NMR (300 MHz, CDCl_3_): δ_H_ (ppm) = 7.64–7.33 (m, 8H), 7.02 (dd, *J* = 4.9, 3.7 Hz, 1H), 5.81 (s, 1H), 3.04 (s, 6H). ^13^C­{^1^H}-NMR (176 MHz, CDCl_3_): δ_C_ (ppm) = 180.1, 159.2, 149.4, 141.2, 129.9, 129.3, 129.2, 128.6, 127.7, 127.4, 102.4, 77.7, 44.3. HRMS (ESI, Orbitrap): calc. for C_16_H_17_O_2_S_2_ [M + H]^+^: 305.0664, found: 305.0663.

### General Procedures (GP2) for the Synthesis of Alkylidene Cyclobutanones **3**

A flame-dried microwave vial was charged with SCP **1** (0.1 mmol, 1.0 equiv) and sulfoxonium ylide **2** (0.15 mmol, 1.5 equiv). The vial was evacuated and backfilled with argon (2 ×) before anhydrous CH_2_Cl_2_ (2 mL, 0.05 M) was added. The reaction mixture was cooled to 0 °C and DIPEA (0.06 mL, 3.4 equiv) was added. The solution was stirred overnight while gradually warming to room temperature. The mixture was extracted with EtOAc (3 × 8 mL). The combined organic phases were dried over MgSO_4_, filtered and concentrated under vacuum. Purification by flash column chromatography (SiO_2_, *n*-pentane/EtOAc) yielded the respective alkylidene cyclobutanones **3**. Structural assignments were made with additional information from COSY, HSQC, and HMBC experiments.

Note: The reported *E*:*Z* ratio was determined from the crude mixture prior to flash chromatography, as indicated by ^1^H NMR analysis. The NMR data correspond to the major diastereomer; only traces of the minor isomer were recovered after purification, suggesting that partial isomerization occurred during this process.

#### Methyl (*E*)-3-(2-Oxo­cyclo­butylidene)-3-phenyl-propanoate (**3aa**)

Prepared according to **GP2** from SCP **1a** (21 mg, 0.10 mmol, 1.0 equiv) and sulfoxonium ylide **2a** (38 mg, 0.15 mmol, 1.5 equiv). Purification by flash column chromatography (SiO_2_, *n*-pentane/EtOAc 19:1) afforded the cyclobutanone **3aa** (19 mg, 0.08 mmol, 84% yield, 10:1 *E*:*Z*) as a colorless solid. On a 1.0 mmol scale: Prepared according to **GP2**. A flame-dried 50 mL Schlenk flask was charged with **1a** (212 mg, 1.0 mmol, 1.0 equiv) and sulfoxonium ylide **2a** (379 mg, 1.5 mmol, 1.5 equiv). The vial was evacuated and backfilled with argon (2 ×) before anhydrous CH_2_Cl_2_ (20 mL, 0.05 M) was added. The reaction mixture was cooled to 0 °C and DIPEA (0.6 mL, 3.4 equiv) was added. The solution was stirred overnight while gradually warming to room temperature. The mixture was extracted with EtOAc (3 × 80 mL). The combined organic phases were dried over MgSO_4_, filtered and concentrated under vacuum. Purification by flash column chromatography (SiO_2_, *n*-pentane/EtOAc 19:1) afforded the cyclobutanone **3aa** (184 mg, 0.8 mmol, 80% yield, 10:1 *E*:*Z*). Colorless, block-shaped crystals of **3aa** were obtained at room temperature by slow solvent evaporation from a solution of the compound dissolved in a mixture of dichloromethane and pentane. R_f_ = 0.08 (*n*-pentane/EtOAc 19:1). M.P. = 119–120 °C. FTIR (ATR): *ṽ* [cm^–1^] = 2914, 1721, 1624, 1432, 1419, 1389, 1333, 1233, 1210, 1190, 1162, 1127, 1105, 1086, 1066, 1044, 985. ^1^H NMR (700 MHz, CDCl_3_): δ_H_ (ppm) = 7.53–7.48 (m, 2H), 7.43–7.34 (m, 3H), 4.12 (t, *J* = 1.4 Hz, 2H), 3.64 (s, 3H), 3.07–3.02 (m, 2H), 2.92 (ddt, *J* = 9.8, 7.4, 1.3 Hz, 2H). ^13^C­{^1^H}-NMR (176 MHz, CDCl_3_): δ_C_ (ppm) = 199.6, 170.8, 145.7, 137.1, 134.9, 129.4, 128.7, 127.5, 52.1, 44.7, 36.3, 23.8. HRMS (GC-APCI, QTOF): calc. for C_14_H_15_O_3_ [M + H]^+^: 231.1016, found: 231.1014.

#### Methyl (*E*)-3-(4-Fluoro­phenyl)-3-(2-oxo­cyclo­butylidene)­pro­panoate (**3ab**)

Prepared according to **GP2** from SCP **1a** (21 mg, 0.10 mmol, 1.0 equiv) and sulfoxonium ylide **2b** (41 mg, 0.15 mmol, 1.5 equiv). Purification by flash column chromatography (SiO_2_, *n*-pentane/EtOAc 19:1) afforded the cyclobutanone **3ab** (17 mg, 0.07 mmol, 68% yield, 5.6:1 *E*:*Z*) as a colorless solid. R_f_ = 0.28 (*n*-pentane/EtOAc 9:1). M.P. = 99–100 °C. FTIR (ATR): *ṽ* [cm^–1^] = 2935, 1736, 1721, 1626, 1597, 1586, 1507, 1433, 1422, 1392, 1329, 1304, 1242,1217, 1185, 1158, 1124, 1105, 1067, 1041, 1006, 982. ^1^H NMR (400 MHz, CDCl_3_): δ_H_ (ppm) = 7.54–7.47 (m, 2H), 7.13–7.06 (m, 2H), 4.10 (t, *J* = 1.3 Hz, 2H), 3.64 (s, 3H), 3.08–3.01 (m, 2H), 2.93–2.84 (m, 2H). ^13^C­{^1^H}-NMR (101 MHz, CDCl_3_): δ_C_ (ppm) = 199.5, 170.9, 163.3 (d, *J* = 251.0 Hz), 145.7, 133.9, 133.3 (d, *J* = 3.5 Hz), 129.7 (d, *J* = 8.4 Hz), 116.0 (d, *J* = 21.7 Hz), 52.3, 44.9, 36.5, 23.9. ^19^F-NMR (377 MHz, CDCl_3_) δ (ppm) = −110.49 (m). HRMS (GC-APCI, QTOF): calc. for C_14_H_14_FO_3_ [M + H]^+^: 249.0921, found: 249.0922.

#### Methyl (*E*)-3-(4-Chlorophenyl)-3-(2-oxo­cyclo­butylidene)­pro­panoate (**3ac**)

Prepared according to **GP2** from SCP **1a** (21 mg, 0.10 mmol, 1.0 equiv) and sulfoxonium ylide **2c** (43 mg, 0.15 mmol, 1.5 equiv). Purification by flash column chromatography (SiO_2_, *n*-pentane/EtOAc 19:1) afforded the cyclobutanone **3ac** (15 mg, 0.06 mmol, 57% yield, 5.3:1 *E*:*Z*) colorless solid. R_f_ = 0.29 (*n*-pentane/EtOAc 9:1). M.P. = 106–107 °C. FTIR (ATR): *ṽ* [cm^–1^] = 2954, 1724, 1624, 1587, 1493, 1400, 1389, 1341, 1318, 1251, 1189, 1170, 1124, 1097, 1063, 10049, 1036, 1008, 982. ^1^H NMR (400 MHz, CDCl_3_): δ_H_ (ppm) = 7.48–7.41 (m, 2H), 7.41–7.35 (m, 2H), 4.09 (t, *J* = 1.3 Hz, 2H), 3.64 (s, 3H), 3.09–3.00 (m, 2H), 2.89 (ddt, *J* = 10.2, 7.5, 1.4 Hz, 2H). ^13^C­{^1^H}-NMR (101 MHz, CDCl_3_): δ_C_ (ppm) = 199.5, 170.8, 146.3, 135.7, 135.6, 133.8, 129.2, 129.0, 52.3, 45.0, 36.3, 24.0. HRMS (GC-APCI, QTOF): calc. for C_14_H_14_ClO_3_ [M + H]^+^: 265.0626, found: 265.0626.

#### Methyl (*E*)-3-(4-Methoxyphenyl)-3-(2-oxocyclobutyli-dene)­propanoate (**3ad**)

Prepared according to **GP2** from SCP **1a** (21 mg, 0.10 mmol, 1.0 equiv) and sulfoxonium ylide **2d** (42 mg, 0.15 mmol, 1.5 equiv). Purification by flash column chromatography (SiO_2_, *n*-pentane/EtOAc 9:1) afforded the cyclobutanone **3ad** (16 mg, 0.06 mmol, 61% yield, 7.7:1 *E*:*Z*) as a colorless solid. R_f_ = 0.14 (*n*-pentane/EtOAc 9:1). M.P. = 98–99 °C. FTIR (ATR): *ṽ* [cm^–1^] = 2935, 1724, 1715, 1621, 1593, 1514, 1438, 1416, 1311, 1251, 1230, 1188, 1180, 1138, 1121, 1063, 1041, 1018, 1004, 848. ^1^H NMR (300 MHz, CDCl_3_): δ_H_ (ppm) = 7.54–7.47 (m, 2H), 6.96–6.88 (m, 2H), 4.13 (t, *J* = 1.2 Hz, 2H), 3.84 (s, 3H), 3.64 (s, 3H), 3.03 (ddd, *J* = 8.7, 6.4, 1.7 Hz, 2H), 2.95–2.84 (m, 2H). ^13^C­{^1^H}-NMR (101 MHz, CDCl_3_): δ_C_ (ppm) = 199.4, 170.9, 160.5, 143.6, 134.3, 129.1, 129.1, 114.0, 55.2, 51.9, 44.5, 35.9, 23.9. HRMS (ESI, Orbitrap): calc. for C_15_H_16_O_4_Na [M + Na]^+^: 283.0941, found: 283.0944.

#### Methyl (*E*)-3-(3,4-Dimethoxyphenyl)-3-(2-oxocyclobutylidene)­propanoate (**3ae**)

Prepared according to **GP2** from SCP **1a** (21 mg, 0.10 mmol, 1.0 equiv) and sulfoxonium ylide **2e** (47 mg, 0.15 mmol, 1.5 equiv). Purification by flash column chromatography (SiO_2_, *n*-pentane/EtOAc 9:1 to 4:1) afforded the cyclobutanone **3ae** (19 mg, 0.07 mmol, 65% yield, 5.6:1 *E*:*Z*) as a pale yellow solid. R_f_ = 0.14 (*n*-pentane/EtOAc 4:1). M.P. = 89–90 °C. FTIR (ATR): *ṽ* [cm^–1^] = 2942, 2841, 1723, 1620, 1593, 1573, 1518, 1465, 1433, 1333, 1251, 1231, 1189, 1172, 1122, 1063, 1003, 873. ^1^H NMR (400 MHz, CDCl_3_): δ_H_ (ppm) = 7.18–7.07 (m, 2H), 6.89 (d, *J* = 8.4 Hz, 1H), 4.16–4.12 (m, 2H), 3.91 (s, 3H), 3.90 (s, 3H), 3.64 (s, 3H), 3.08–3.02 (m, 2H), 2.96 (ddt, *J* = 8.1, 6.0, 1.6 Hz, 2H).^13^C­{^1^H}-NMR (101 MHz, CDCl_3_): δ_C_ (ppm) = 199.6, 171.2, 150.5, 149.0, 144.1, 134.8, 129.8, 121.5, 111.2, 110.7, 56.1, 56.0, 52.3, 44.9, 36.3, 24.3. HRMS (GC-APCI, QTOF): calc. for C_16_H_19_O_5_ [M + H]^+^: 291.1227, found: 291.1229.

#### Methyl (*E*)-3-(2-Oxocyclobutylidene)-3-(p-tolyl)­propanoate (**3af**)

Prepared according to **GP2** from SCP **1a** (21 mg, 0.10 mmol, 1.0 equiv) and sulfoxonium ylide **2f** (40 mg, 0.15 mmol, 1.5 equiv). Purification by flash column chromatography (SiO_2_, *n*-pentane/EtOAc 19:1) afforded the cyclobutanone **3af** (15 mg, 0.06 mmol, 60% yield, 10:1 *E*:*Z*) as a colorless solid. R_f_ = 0.14 (*n*-pentane/EtOAc 19:1). M.P. = 90–91 °C. FTIR (ATR): *ṽ* [cm^–1^] = 2956, 1722, 1624, 1563, 1513, 1426, 1390, 1313, 1246, 1189, 1122, 1052, 1010, 924. ^1^H NMR (400 MHz, CDCl_3_): δ_H_ (ppm) = 7.45–7.38 (m, 2H), 7.23–7.16 (m, 2H), 4.12 (t, *J* = 1.3 Hz, 2H), 3.63 (s, 3H), 3.08–3.00 (m, 2H), 2.92 (ddt, *J* = 9.8, 7.6, 1.4 Hz, 2H), 2.37 (s, 3H).^13^C­{^1^H}-NMR (101 MHz, CDCl_3_): δ_C_ (ppm) = 199.5, 170.8, 144.8, 139.7, 134.7, 134.0, 129.3, 127.4, 51.9, 44.5, 36.0, 23.8, 21.2. HRMS (GC-APCI, QTOF): calc. for C_15_H_17_O_3_ [M + H]^+^: 245.1172, found: 245.1171.

#### Ethyl (*E*)-3-(2-Oxocyclobutylidene)-3-phenylpropanoate (**3ag**)

Prepared according to **GP2** from SCP **1a** (21 mg, 0.10 mmol, 1.0 equiv) and sulfoxonium ylide **2g** (40 mg, 0.15 mmol, 1.5 equiv). Purification by flash column chromatography (SiO_2_, *n*-pentane/EtOAc 19:1) afforded the cyclobutanone **3ag** (17 mg, 0.07 mmol, 69% yield, 10:1 *E*:*Z*) as a pale yellow oil. R_f_ = 0.11 (*n*-pentane/EtOAc 19:1). FTIR (ATR): *ṽ* [cm^–1^] = 2928, 1729, 1632, 1574, 1495, 1445, 1420, 1392, 1367, 1321, 1234, 1165, 1125, 1059, 1041, 1024, 922. ^1^H NMR (700 MHz, CDCl_3_): δ_H_ (ppm) = 7.52–7.49 (m, 2H), 7.42–7.35 (m, 3H), 4.11–4.06 (m, 4H), 3.06–3.01 (m, 2H), 2.92 (ddt, *J* = 8.6, 6.2, 1.3 Hz, 2H), 1.16 (t, *J* = 7.1 Hz, 3H). ^13^C­{^1^H}-NMR (176 MHz, CDCl_3_): δ_C_ (ppm) = 200.0, 170.7, 146.0, 137.6, 135.5, 129.7, 129.0, 127.9, 61.3, 45.1, 37.0, 24.1, 14.4. HRMS (GC-APCI, QTOF): calc. for C_15_H_17_O_3_ [M + H]^+^: 245.1172, found: 245.1177.

#### Allyl (*E*)-3-(2-Oxocyclobutylidene)-3-phenylpropanoate (**3ah**)

Prepared according to **GP2** from SCP **1a** (21 mg, 0.10 mmol, 1.0 equiv) and sulfoxonium ylide **2h** (42 mg, 0.15 mmol, 1.5 equiv). Purification by flash column chromatography (SiO_2_, *n*-pentane/EtOAc 29:1) afforded the cyclobutanone **3ah** (20 mg, 0.08 mmol, 79% yield, 13:1 *E*:*Z*) as a colorless solid. R_f_ = 0.21 (*n*-pentane/EtOAc 10:1). M.P. = 151–152 °C. FTIR (ATR): *ṽ* [cm^–1^] = 2937, 1785, 1730, 1632, 1573, 1495, 1445, 1419, 1393, 1364, 1325, 1231, 1158, 1040, 985.^1^H NMR (400 MHz, CDCl_3_): δ_H_ (ppm) = 7.55–7.45 (m, 2H), 7.45–7.32 (m, 3H), 5.81 (ddt, *J* = 17.2, 10.5, 5.6 Hz, 1H), 5.27–5.12 (m, 2H), 4.54 (dt, *J* = 5.6, 1.5 Hz, 2H), 4.15 (t, *J* = 1.3 Hz, 2H), 3.09–2.99 (m, 2H), 2.92 (ddt, *J* = 10.0, 7.5, 1.4 Hz, 2H). ^13^C­{^1^H}-NMR (101 MHz, CDCl_3_): δ_C_ (ppm) = 199.8, 170.2, 145.9, 137.3, 135.0, 132.0, 129.6, 128.9, 127.7, 118.3, 65.7, 44.9, 36.6, 23.9. HRMS (GC-APCI, QTOF): calc. for C_16_H_17_O_3_ [M + H]^+^: 257.1172, found: 257.1177.

#### Benzyl (*E*)-3-(2-Oxocyclobutylidene)-3-phenylpropanoate (**3ai**)

Prepared according to **GP2** from SCP **1a** (21 mg, 0.10 mmol, 1.0 equiv) and sulfoxonium ylide **2i** (49 mg, 0.15 mmol, 1.5 equiv). Purification by flash column chromatography (SiO_2_, *n*-pentane/EtOAc 20:1 to 10:1) afforded the cyclobutanone **3ai** (23 mg, 0.08 mmol, 75% yield, 13:1 *E*:*Z*) as a colorless oil. R_f_ = 0.24 (*n*-pentane/EtOAc 10:1). FTIR (ATR): *ṽ* [cm^–1^] = 2935, 1728, 1630, 1573, 1496, 1455, 1445, 1418, 1392, 1377, 1325, 1231, 1153, 1125, 1082, 1059, 1041, 989. ^1^H NMR (400 MHz, CDCl_3_): δ_H_ (ppm) = 7.52–7.44 (m, 2H), 7.41–7.35 (m, 3H), 7.34–7.26 (m, 3H), 7.23–7.16 (m, 2H), 5.07 (s, 2H), 4.18 (t, *J* = 1.3 Hz, 2H), 3.09–2.99 (m, 2H), 2.92 (ddt, *J* = 10.4, 6.1, 1.3 Hz, 2H). ^13^C­{^1^H}-NMR (101 MHz, CDCl_3_): δ_C_ (ppm) = 199.8, 170.3, 145.9, 137.2, 135.8, 135.0, 129.6, 128.9, 128.6, 128.2, 128.1, 127.7, 66.8, 44.9, 36.7, 23.9. HRMS (GC-APCI, QTOF): calc. for C_20_H_19_O_3_ [M + H]^+^: 307.1329, found: 307.1336.

#### (*E*)-2-(3-Oxo-1,3-diphenylpropylidene)­cyclobutan-1-one (**3aj**)

Prepared according to **GP2** from SCP **1a** (21 mg, 0.10 mmol, 1.0 equiv) and sulfoxonium ylide **2j** (45 mg, 0.15 mmol, 1.5 equiv). Purification by flash column chromatography (SiO_2_, *n*-pentane/EtOAc 20:1 to 10:1) afforded the cyclobutanone **3aj** (17 mg, 0.06 mmol, 61% yield) as a yellow solid. Colorless, block-shaped crystals of **3aj** were obtained at room temperature by slow diffusion of pentane into a solution of the compound dissolved in dichloromethane by the aid of layering. R_f_ = 0.20 (*n*-pentane/EtOAc 10:1). M.P. = 149–150 °C. FTIR (ATR): *ṽ* [cm^–1^] = 2938, 1980, 1747, 1726, 1672, 1642, 1615, 1594, 1571, 1494, 1446, 1420, 1396, 1388, 1331, 1302, 1236, 1215, 1199, 1183, 1125, 1103, 1079. ^1^H NMR (500 MHz, CDCl_3_): δ_H_ (ppm) = 8.07–7.95 (m, 2H), 7.62–7.53 (m, 1H), 7.50–7.43 (m, 4H), 7.39–7.31 (m, 3H), 4.78 (t, *J* = 1.2 Hz, 2H), 3.09–3.02 (m, 2H), 2.98–2.91 (m, 2H). ^13^C­{^1^H}-NMR (126 MHz, CDCl_3_): δ_C_ (ppm) = 200.0, 196.7, 145.7, 137.7, 137.0, 136.9, 133.4, 129.4, 128.8, 128.8, 128.4, 127.9, 44.8, 41.6, 24.0. HRMS (GC-APCI, QTOF): calc. for C_19_H_17_O_2_ [M + H]^+^: 277.1223, found: 277.1226.

#### (*E*)-2-(3-(4-Meth­oxyphenyl)-3-oxo-1-phenyl­propylidene)­cyclo­butan-1-one (**3ak**)

Prepared according to **GP2** from SCP **1a** (21 mg, 0.10 mmol, 1.0 equiv) and sulfoxonium ylide **2k** (49 mg, 0.15 mmol, 1.5 equiv). Purification by flash column chromatography (SiO_2_, *n*-pentane/EtOAc 20:1 to 4:1) afforded the cyclobutanone **3ak** (17 mg, 0.06 mmol, 56% yield) as a colorless solid. Colorless, block-shaped crystals of **3ak** were obtained at room temperature by slow diffusion of pentane into a solution of the compound dissolved in dichloromethane by the aid of layering. R_f_ = 0.30 (*n*-pentane/EtOAc 4:1). M.P. = 159–160 °C. FTIR (ATR): *ṽ* [cm^–1^] = 2961, 2931, 2841, 2076, 1984, 1902, 1758, 1722, 1666. 1594, 1571, 1509, 1456, 1441, 1386, 1331, 1306, 1262, 1224, 1191, 1168, 1126, 1026, 999. ^1^H NMR (700 MHz, CDCl_3_): δ_H_ (ppm) = 8.01–7.97 (m, 2H), 7.49–7.45 (m, 2H), 7.38–7.31 (m, 3H), 6.96–6.92 (m, 2H), 4.73 (t, *J* = 1.3 Hz, 2H), 3.87 (s, 3H), 3.06–3.01 (m, 2H), 2.94 (ddt, *J* = 9.6, 7.2, 1.3 Hz, 2H). ^13^C­{^1^H}-NMR (176 MHz, CDCl_3_): δ_C_ (ppm) = 200.0, 195.1, 164.0, 145.6, 137.7, 137.4, 130.8, 130.0, 129.4, 128.7, 127.9, 113.9, 55.6, 44.8, 41.3, 23.9. HRMS (ESI, Orbitrap): calc. for C_20_H_19_O_3_ [M + H]^+^: 307.1329, found: 307.1323.

#### (*E*)-*N*-Methyl-3-(2-oxocyclobutylidene)-*N*,3-diphenylpropanamide (**3al**)

Prepared according to **GP2** from SCP **1a** (21 mg, 0.10 mmol, 1.0 equiv) and sulfoxonium ylide **2l** (49 mg, 0.15 mmol, 1.5 equiv). Purification by flash column chromatography (SiO_2_, *n*-pentane/EtOAc 10:1 to 2:1) afforded the cyclobutanone **3al** (18 mg, 0.06 mmol, 59% yield) as a yellow oil. R_f_ = 0.40 (*n*-pentane/EtOAc 2:1). FTIR (ATR): *ṽ* [cm^–1^] = 3054, 2930, 1793, 1649, 1594, 1573, 1494, 1444, 1418, 1373, 1303, 1260, 1227, 1186, 1118, 1088, 1071, 1044, 1023, 1002, 918. ^1^H NMR (700 MHz, CDCl_3_): δ_H_ (ppm) = 7.46–7.40 (m, 4H), 7.39–7.30 (m, 4H), 7.15–7.12 (m, 2H), 3.32 (s, 2H), 3.22 (s, 3H), 2.83 (dd, *J* = 8.8, 7.0 Hz, 2H), 2.55 (t, *J* = 7.9 Hz, 2H). ^13^C­{^1^H}-NMR (176 MHz, CDCl_3_): δ_C_ (ppm) = 196.1, 168.7, 146.7, 143.8, 138.8, 136.7, 130.1, 129.3, 128.3, 128.2, 128.1, 127.5, 42.0, 40.1, 37.7, 21.3. HRMS (GC-APCI, QTOF): calc. for C_20_H_20_NO_2_ [M + H]^+^: 306.1483, found: 306.1489.

#### (*E*)-*N*-(4-Methoxyphenyl)-*N*-methyl-3-(2-oxocyclobutylidene)-3-phenylpropanamide (**3am**)

Prepared according to **GP2** from SCP **1a** (21 mg, 0.10 mmol, 1.0 equiv) and sulfoxonium ylide **2m** (54 mg, 0.15 mmol, 1.5 equiv). Purification by flash column chromatography (SiO_2_, *n*-pentane/EtOAc 4:1 to 1:1) afforded the cyclobutanone **3am** (18 mg, 0.05 mmol, 52% yield) as a yellow oil. R_f_ = 0.38 (*n*-pentane/EtOAc 1:1). FTIR (ATR): *ṽ* [cm^–1^] = 2933, 2838, 1732,1645, 1573, 1508, 1463, 1443, 1421, 1375, 1292, 1245, 1181, 1170, 1105, 1088, 1071, 1044, 1027, 918. ^1^H NMR (400 MHz, CDCl_3_): δ_H_ (ppm) = 7.44–7.37 (m, 2H), 7.37–7.29 (m, 3H), 7.06–7.01 (m, 2H), 6.95–6.88 (m, 2H), 3.83 (s, 3H), 3.31 (d, *J* = 1.2 Hz, 2H), 3.18 (s, 3H), 2.87–2.77 (m, 2H), 2.63–2.52 (m, 2H). ^13^C­{^1^H}-NMR (101 MHz, CDCl_3_): δ_C_ (ppm) = 196.1, 169.1, 159.3, 146.6, 138.9, 136.8, 136.5, 129.2, 128.5, 128.2, 128.1, 115.2, 55.7, 42.0, 40.0, 37.8, 21.3. HRMS (ESI, Orbitrap): calc. for C_21_H_21_O_3_NNa [M + Na]^+^: 358.1414, found: 358.1409.

#### (*E*)-2-(3-Oxo-1-phenyl-3-(thiophen-2-yl)­propylidene)­cyclobutan-1-one (**3an**)

Prepared according to **GP2** from SCP **1a** (21 mg, 0.10 mmol, 1.0 equiv) and sulfoxonium ylide **2n** (46 mg, 0.15 mmol, 1.5 equiv). Purification by flash column chromatography (SiO_2_, *n*-pentane/EtOAc 8:1 to 6:1) afforded the cyclobutanone **3an** (17 mg, 0.06 mmol, 60% yield) as a yellow solid. R_f_ = 0.18 (*n*-pentane/EtOAc 8:1). M.P. = 119–120 °C. FTIR (ATR): *ṽ* [cm^–1^] = 3103, 2927, 1723, 1654, 1614, 1592, 1571, 1513, 1496, 1442, 1410, 1386, 1354, 1322, 1300, 1232, 1223, 1191, 1127, 1980, 1067, 1051,1039, 998. ^1^H NMR (400 MHz, CDCl_3_): δ_H_ (ppm) = 7.90 (dd, *J* = 3.8, 1.1 Hz, 1H), 7.63 (dd, *J* = 5.0, 1.1 Hz, 1H), 7.53–7.48 (m, 2H), 7.42–7.29 (m, 3H), 7.14 (dd, *J* = 5.0, 3.8 Hz, 1H), 4.71 (t, *J* = 1.3 Hz, 2H), 3.14–3.01 (m, 2H), 2.98–2.88 (m, 2H). ^13^C­{^1^H}-NMR (101 MHz, CDCl_3_): δ_C_ (ppm) = 200.0, 189.1, 145.9, 143.8, 137.4, 136.4, 134.1, 132.7, 129.5, 128.8, 128.3, 128.0, 44.8, 42.0, 24.0. HRMS (GC-APCI, QTOF): calc. for C_17_H_15_O_2_S [M + H]^+^: 283.0787, found: 283.0791.

#### Methyl (*S*,*E*)-3-(2-Methyl-4-oxocyclobutylidene)-3-phenylpropanoate (**3ba**)

Prepared according to **GP2** from SCP **1b** (21 mg, 0.10 mmol, 1.0 equiv) and sulfoxonium ylide **2a** (38 mg, 0.15 mmol, 1.5 equiv). Purification by flash column chromatography (SiO_2_, *n*-pentane/EtOAc 19:1) afforded the cyclobutanone **3ba** (14 mg, 0.06 mmol, 59% yield, 5.8:1 *E*:*Z*, 99% *ee*) as a colorless solid. Colorless, block-shaped crystals of **3ba** were obtained at room temperature by slow solvent evaporation from a solution of the compound dissolved in a mixture of pentane and dichloromethane. R_f_ = 0.11 (*n-*pentane/EtOAc 19:1). [α]_D_^20^ =–75 (*c* 0.4, CH_2_Cl_2_). M.P. = 78–79 °C. FTIR (ATR): *ṽ* [cm^–1^] = 2869, 1728, 1629, 1574, 1495, 1444, 1433, 1393, 1317, 1298, 1256, 1234, 1174, 1157, 1130, 1083, 1057, 1130, 1083, 1057, 1130, 1083, 1057, 1011, 982. ^1^H NMR (700 MHz, CDCl_3_): δ_H_ (ppm) = 7.45–7.34 (m, 5H), 4.26 (dd, *J* = 16.4, 0.7 Hz, 1H), 3.87 (dd, *J* = 16.4, 1.5 Hz, 1H), 3.61 (s, 3H), 3.49–3.39 (m, 1H), 3.24 (dd, *J* = 17.4, 8.8 Hz, 1H), 2.57 (dd, *J* = 17.4, 5.3 Hz, 1H), 1.08 (d, *J* = 6.8 Hz, 3H). ^13^C­{^1^H}-NMR (176 MHz, CDCl_3_): δ_C_ (ppm) = 199.9, 170.6, 150.2, 136.8, 136.2, 129.1, 128.6, 127.7, 52.5, 52.0, 37.3, 30.3, 17.9. HRMS (GC-APCI, QTOF): calc. for C_15_H_17_O_3_ [M + H]^+^: 245.1172, found: 245.1174.

#### Methyl (*R*,*E*)-3-(2-Oxo-4-phenylcyclobutylidene)-3-phenylpropanoate (**3ca**)

Prepared according to **GP2** from SCP **1c** (27 mg, 0.10 mmol, 1.0 equiv) and sulfoxonium ylide **2a** (38 mg, 0.15 mmol, 1.5 equiv). Purification by flash column chromatography (SiO_2_, *n*-pentane/EtOAc 19:1) afforded the cyclobutanone **3ca** (17 mg, 0.05 mmol, 54% yield, 5.0:1 *E*:*Z*, 51% *ee*) as a pale yellow oil. R_f_ = 0.11 (*n*-pentane/EtOAc 19:1). [α]_D_^20^ = +57 (*c* 0.4, CH_2_Cl_2_). FTIR (ATR): *ṽ* [cm^–1^] = 2953, 1732, 1625, 1494, 1444, 1434, 1326, 1250, 1219, 1193, 1165, 1144, 1077, 1049, 1028, 1013, 959. ^1^H NMR (700 MHz, CDCl_3_): δ_H_ (ppm) = 7.51–7.44 (m, 1H), 7.41–7.34 (m, 3H), 7.31–7.26 (m, 4H), 7.26–7.24 (m, 1H), 7.22–7.18 (m, 1H), 4.58 (ddt, *J* = 9.3, 5.5, 1.2 Hz, 1H), 4.28 (dd, *J* = 4.4, 1.1 Hz, 2H), 3.78 (s, 3H), 3.68 (dd, *J* = 17.5, 9.2 Hz, 1H), 3.07 (dd, *J* = 17.5, 5.5 Hz, 1H). ^13^C­{^1^H}-NMR (176 MHz, CDCl_3_): δ_C_ (ppm) = 199.3, 170.8, 148.2, 142.2, 137.7, 136.3, 129.5, 128.7, 128.4, 128.1, 127.2, 126.7, 55.6, 52.3, 41.2, 37.3. HRMS (ESI, Orbitrap): calc. for C_20_H_18_O_3_Na [M + Na]^+^: 329.1148, found: 329.1151.

#### Methyl (*R*,*E*)-3-(2-(4-Fluorophenyl)-4-oxocyclobutylidene)-3-phenylpropanoate (**3da**)

Prepared according to **GP2** from SCP **1d** (29 mg, 0.10 mmol, 1.0 equiv) and sulfoxonium ylide **2a** (38 mg, 0.15 mmol, 1.5 equiv). Purification by flash column chromatography (SiO_2_, *n*-pentane/EtOAc 19:1 to 9:1) afforded the cyclobutanone **3da** (17 mg, 0.05 mmol, 53% yield, 6.0:1 *E*:*Z*, 30% *ee*) as a pale yellow solid. R_f_ = 0.14 (*n*-pentane/EtOAc 19:1). [α]_D_^20^ = +15 (*c* 0.3, CH_2_Cl_2_). M.P. = 97–98 °C. FTIR (ATR): *ṽ* [cm^–1^] = 2926, 1733, 1627, 1604, 1508, 1444, 1435, 1418, 1330, 1221, 1193, 1160, 1097, 1056, 1045, 1014, 841. ^1^H NMR (300 MHz, CDCl_3_): δ_H_ (ppm) = 7.49–7.37 (m, 1H), 7.36–7.28 (m, 2H), 7.26–7.07 (m, 4H), 6.96–6.82 (m, 2H), 4.54 (dd, *J* = 9.2, 5.5 Hz, 1H), 4.30–4.13 (m, 2H), 3.75 (s, 3H), 3.68–3.60 (m, 1H), 2.98 (dd, *J* = 17.5, 5.5 Hz, 1H). ^13^C­{^1^H}-NMR (176 MHz, CDCl_3_): δ_C_ (ppm) = 199.1, 171.0, 161.8 (d, *J* = 244.9 Hz), 148.4, 138.2, 137.9 (d, *J* = 3.3 Hz), 136.4, 129.8, 128.9 (d, *J* = 8.0 Hz), 128.7, 128.2, 115.7 (d, *J* = 21.4 Hz), 55.9, 52.6, 40.6, 37.6. ^19^F NMR (659 MHz, CDCl_3_) δ­(ppm) =–116.2. HRMS (ESI, Orbitrap): calc. for C_20_H_17_O_3_FNa [M + Na]^+^: 347.1054, found: 347.1048.

#### Methyl (*R*,*E*)-3-(2-(4-Chlorophenyl)-4-oxocyclobutylidene)-3-phenylpropanoate (**3ea**)

Prepared according to **GP2** from SCP **1e** (31 mg, 0.10 mmol, 1.0 equiv) and sulfoxonium ylide **2a** (38 mg, 0.15 mmol, 1.5 equiv). Purification by flash column chromatography (SiO_2_, *n*-pentane/EtOAc 19:1 to 9:1) afforded the cyclobutanone **3ea** (17 mg, 0.05 mmol, 49% yield, 5.6:1 *E*:*Z*, 50% *ee*) as a colorless oil. R_f_ = 0.37 (*n*-pentane/EtOAc 9:1). [α]_D_^20^ = +59 (*c* 0.2, CH_2_Cl_2_). FTIR (ATR): *ṽ* [cm^–1^] = 2919, 2851, 1739, 1628, 1490, 1434, 1417, 1370, 1243, 1172, 1092, 824. ^1^H NMR (400 MHz, CDCl_3_): δ_H_ (ppm) = 7.34–7.27 (m, 1H), 7.26–7.17 (m, 4H), 7.15–7.06 (m, 4H), 4.47 (dd, *J* = 9.2, 5.4 Hz, 1H), 4.22–4.08 (m, 2H), 3.69 (s, 3H), 3.58 (dd, *J* = 17.5, 9.2 Hz, 1H), 2.92 (dd, *J* = 17.5, 5.5 Hz, 1H). ^13^C­{^1^H}-NMR (101 MHz, CDCl_3_): δ_C_ (ppm) = 198.7, 170.8, 147.9, 140.6, 138.2, 136.2, 132.4, 129.7, 129.3, 128.8, 128.6, 128.0, 55.6, 52.4, 40.5, 37.4. HRMS (GC-APCI, QTOF): calc. for C_20_H_18_O_3_Cl [M + H]^+^: 341.0939, found: 341.0937.

#### Methyl (*E*)-3-(2-Oxo-4-(p-tolyl)­cyclobutylidene)-3-phenylpropanoate (**3fa**)

Prepared according to **GP2** from SCP **1f** (29 mg, 0.10 mmol, 1.0 equiv) and sulfoxonium ylide **2a** (38 mg, 0.15 mmol, 1.5 equiv). Purification by flash column chromatography (SiO_2_, *n*-pentane/EtOAc 19:1) afforded the cyclobutanone **3fa** (13 mg, 0.04 mmol, 42% yield, 3.7:1 *E*:*Z*, *rac*.) as a pale yellow oil. R_f_ = 0.42 (*n*-pentane/EtOAc 9:1). FTIR (ATR): *ṽ* [cm^–1^] = 2920, 2853, 1740, 1629, 1458, 1417, 1376, 1242, 1220, 1167, 966, 724. ^1^H NMR (700 MHz, CDCl_3_): δ_H_ (ppm) = 7.31–7.29 (m, 2H), 7.23–7.16 (m, 3H), 7.06–7.03 (m, 2H), 6.99–6.96 (m, 2H), 4.45 (dd, *J* = 9.2, 5.4 Hz, 1H), 4.18 (dd, *J* = 2.5, 1.1 Hz, 2H), 3.67 (s, 3H), 3.57 (dd, *J* = 17.5, 9.2 Hz, 1H), 2.93 (dd, *J* = 17.5, 5.3 Hz, 1H), 2.24 (s, 3H). ^13^C­{^1^H}-NMR (176 MHz, CDCl_3_): δ_C_ (ppm) = 199.6, 170.9, 148.3, 139.3, 137.5, 136.3, 136.2, 129.5, 129.4, 128.5, 128.2, 127.1, 55.8, 52.3, 40.9, 37.3, 21.1. HRMS (GC-APCI, QTOF): calc. for C_21_H_21_O_3_ [M + H]^+^: 321.1485, found: 321.1479.

#### Methyl (*E*)-3-(2-(4-Methoxyphenyl)-4-oxocyclobutylidene)-3-phenylpropanoate (**3ga**)

Prepared according to **GP2** from SCP **1g** (30 mg, 0.10 mmol, 1.0 equiv) and sulfoxonium ylide **2a** (38 mg, 0.15 mmol, 1.5 equiv). Purification by flash column chromatography (SiO_2_, *n*-pentane/EtOAc 9:1 to 4:1) afforded the cyclobutanone **3ga** (10 mg, 0.03 mmol, 30% yield, 5.0:1 *E*:*Z*, *rac*.) as a colorless solid. M.P. = 115–116 °C. FTIR (ATR): *ṽ* [cm^–1^] = 2960, 2834, 1735, 1723, 1641, 1613, 1513, 1414, 1323, 1244, 1214, 1178, 1168, 1029, 842. ^1^H NMR (300 MHz, CDCl_3_): δ_H_ (ppm) = 7.34–7.26 (m, 2H), 7.25–7.14 (m, 3H), 7.10–7.05 (m, 2H), 6.75–6.66 (m, 2H), 4.44 (dd, *J* = 9.2, 5.4 Hz, 1H), 4.27–4.09 (m, 2H), 3.72 (s, 3H), 3.68 (s, 3H), 3.62–3.51 (m, 1H), 2.92 (dd, *J* = 17.5, 5.4 Hz, 1H). ^13^C­{^1^H}-NMR (176 MHz, CDCl_3_): δ_C_ (ppm) = 199.7, 170.9, 158.3, 148.5, 137.5, 136.3, 134.4, 129.5, 128.5, 128.2, 128.2, 114.1, 55.8, 55.3, 52.4, 40.5, 37.3. HRMS (GC-APCI, QTOF): calc. for C_21_H_21_O_4_ [M + H]^+^: 337.1434, found: 337.1437.

#### Methyl (*E*)-3-(8-Oxobicyclo­[4.2.0]­octan-7-ylidene)-3-phenylpropanoate (**3ha**)

Prepared according to **GP2** from SCP **1h** (25 mg, 0.10 mmol, 1.0 equiv) and sulfoxonium ylide **2a** (38 mg, 0.15 mmol, 1.5 equiv). Purification by flash column chromatography (SiO_2_, *n*-pentane/EtOAc 19:1) afforded the cyclobutanone **3ha** (12 mg, 0.04 mmol, 41% yield, 4.3:1 *E*:*Z*) as a colorless solid. R_f_ = 0.19 (*n*-pentane/EtOAc 19:1). M.P. = 69–70 °C. FTIR (ATR): *ṽ* [cm^–1^] = 2930, 1740, 1740, 1714, 1621, 1597, 1570, 1432, 1418, 1335, 1290, 1219, 1191, 1152, 1112, 1076, 1061, 1048, 1033, 1022, 999. ^1^H NMR (700 MHz, CDCl_3_): δ_H_ (ppm) = 7.47–7.43 (m, 2H), 7.40–7.33 (m, 3H), 4.12 (dd, *J* = 16.4, 0.8 Hz, 1H), 3.97 (dd, *J* = 16.4, 1.2 Hz, 1H), 3.63 (s, 3H), 3.41–3.35 (m, 1H), 3.27 (ddd, *J* = 9.5, 7.9, 4.4 Hz, 1H), 1.95–1.89 (m, 2H), 1.65–1.60 (m, 1H), 1.51–1.46 (m, 2H), 1.42–1.35 (m, 2H), 1.27–1.22 (m, 1H).^13^C­{^1^H}-NMR (176 MHz, CDCl_3_): δ_C_ (ppm) = 204.0, 170.9, 149.5, 137.3, 134.5, 129.0, 128.6, 127.5, 54.9, 52.0, 37.2, 34.3, 24.8, 20.8, 20.8, 20.5. HRMS (GC-APCI, QTOF): calc. for C_18_H_21_O_3_ [M + H]^+^: 285.1485, found: 285.1487.

#### Methyl (*E*)-3-(2-Hydroxycyclobutylidene)-3-phenylpropanoate (**4**)

A flame-dried microwave vial was charged with alkylidene cyclobutanone **3aa** (23 mg, 0.10 mmol, 1.0 equiv) and evacuated/backfilled with argon (2 ×). Anhydrous MeOH (2.0 mL, 0.05 M) was added, and the solution was cooled in an ice bath. CeCl_3_ (7 mg, 30 mol %) was introduced, and the mixture was stirred for 15 min before NaBH_4_ (6 mg, 0.16 mmol, 1.6 equiv) was added at 0 °C. The reaction was stirred overnight while warming to room temperature, then quenched with aqueous NH_4_Cl (0.3 mL). The solvent was concentrated, and the residue was diluted with H_2_O (3.0 mL). The aqueous layer was extracted with EtOAc (3 × 5 mL), and the combined organic extracts were dried over MgSO_4_ and concentrated under reduced pressure. Purification by flash column chromatography (SiO_2_, *n*-pentane/EtOAc 9:1 to 4:1) to give the corresponding product **4** (12 mg, 0.05 mmol, 52%) as a colorless oil. R_f_ = 0.62 (*n*-pentane/EtOAc 4:1). FTIR (ATR): *ṽ* [cm^–1^] = 3410, 2942, 1724, 1598, 1575, 1495, 1435, 1334, 1231, 1161, 1131, 1086, 1012, 968. ^1^H NMR (500 MHz, CDCl_3_): δ_H_ (ppm) = 7.33 (ddt, *J* = 9.2, 6.9, 1.3 Hz, 2H), 7.25–7.19 (m, 3H), 4.92 (td, *J* = 7.7, 2.9 Hz, 1H), 3.75 (s, 3H), 3.74–3.67 (m, 1H), 3.44 (dq, *J* = 15.3, 1.2 Hz, 1H), 2.63–2.55 (m, 2H), 2.45 (dtd, *J* = 11.1, 8.0, 5.0 Hz, 1H), 1.92 (dddd, *J* = 11.1, 10.2, 9.0, 6.8 Hz, 1H).^13^C­{^1^H}-NMR (126 MHz, CDCl_3_): δ_C_ (ppm) = 174.7, 147.2, 139.4, 128.4, 127.1, 126.9, 125.3, 72.0, 52.7, 37.3, 31.2, 25.3. HRMS (ESI, Orbitrap): calc. for C_14_H_16_O_3_Na [M + Na]^+^: 255.0992, found: 255.0993.

#### (*E*)-2-(3-Hydroxy-3-methyl-1-phenylbutylidene)-1-methylcyclobutan-1-ol (**5**)

A flame-dried microwave vial was charged with **3aa** (23 mg, 0.10 mmol, 1.0 equiv) in anhydrous THF (1.0 mL, 0.1 M) then premade methylmagnesium bromide solution (0.3 mmol, 3.0 eq., 0.1 mL, 3.0 M in THF) was added slowly at–78 °C. The reaction was stirred overnight while warming to room temperature, then quenched with aqueous NH_4_Cl. The mixture was extracted with EtOAc (3 × 5 mL), and the combined organic extracts were dried over MgSO_4_ and concentrated under reduced pressure. Purification by flash column chromatography (SiO_2_, *n*-pentane/EtOAc 5:1 to 4:1) and then prep-TLC (*n*-pentane/EtOAc 4:1) afforded the corresponding product **5** as a colorless oil (12 mg, 0.05 mmol, 49%). R_f_ = 0.25 (*n*-pentane/EtOAc 4:1). FTIR (ATR): *ṽ* [cm^–1^] = 3312, 2969, 2967, 2957, 1599, 1443, 1367, 1248, 1180, 1144, 1072, 963. ^1^H NMR (700 MHz, CDCl_3_): δ_H_ (ppm) = 7.34–7.28 (m, 2H), 7.28–7.26 (m, 2H), 7.22–7.18 (m, 1H), 2.90 (dq, *J* = 14.0, 0.8 Hz, 1H), 2.74 (ddd, *J* = 13.9, 1.7, 0.9 Hz, 1H), 2.69 (dddt, *J* = 16.3, 10.3, 4.3, 0.9 Hz, 1H), 2.30 (ddddd, *J* = 16.1, 9.6, 8.5, 1.6, 1.0 Hz, 1H), 2.09–2.04 (m, 1H), 2.04–1.99 (m, 1H), 1.54 (d, *J* = 0.7 Hz, 3H), 1.22 (d, *J* = 0.5 Hz, 3H), 0.94 (s, 3H). ^13^C­{^1^H}-NMR (176 MHz, CDCl_3_): δ_C_ (ppm) = 147.5, 142.0, 130.2, 128.5, 127.8, 126.6, 77.4, 71.2, 44.0, 35.6, 31.8, 29.6, 26.6, 23.5. HRMS (GC-APCI, QTOF): calc. for C_16_H_22_O_2_Cl [M+Cl]^−^: 281.1314, found: 281.1313.

#### Methyl 2-(5-Oxo-2-phenyl-1,4-dioxaspiro[2.4]­heptan-2-yl)­acetate (**6**)

A flame-dried microwave vial was charged with alkylidene cyclobutanone **3aa** (23 mg, 0.10 mmol, 1.0 equiv), evacuated/backfilled with argon (2 ×), and dissolved in anhydrous CH_2_Cl_2_ (2.0 mL, 0.05 M). *m*-CPBA (52 mg, 77% purity, 3.0 equiv) was added in portions, and the solution was stirred at room temperature for 18 h. The reaction was quenched with 10% w/v aqueous Na_2_SO_3_ (0.5 mL) and stirred for 1 min, followed by extraction with saturated aq. NaHCO_3_ (2 × 1 mL). The organic phase was diluted with H_2_O (5 mL). The mixture was extracted with EtOAc (3 × 5 mL), and the combined organic extracts were dried over MgSO_4_ and concentrated under reduced pressure. Purification by flash column chromatography (SiO_2_, *n*-pentane/EtOAc 9:1 to 4:1) afforded product **6** (13 mg, 0.05 mmol, 50%) as a colorless oil. R_f_ = 0.25 (*n*-pentane/EtOAc 4:1). FTIR (ATR): *ṽ* [cm^–1^] = 1799, 1737, 1498, 1438, 1416, 1350, 1284, 1249, 1167, 1107, 1081, 1049, 982. ^1^H NMR (500 MHz, CDCl_3_): δ_H_ (ppm) = 7.41–7.30 (m, 5H), 3.67 (s, 3H), 3.22 (d, *J* = 2.0 Hz, 2H), 2.86–2.75 (m, 1H), 2.73–2.60 (m, 1H), 2.27–2.10 (m, 2H). ^13^C­{^1^H}-NMR (126 MHz, CDCl_3_): δ_C_ (ppm) = 173.4, 170.1, 135.9, 128.8, 128.6, 126.2, 94.3, 65.1, 52.2, 39.4, 28.3, 24.4. HRMS (GC-APCI, QTOF): calc. for C_14_H_15_O_5_ [M + H]^+^: 263.0914, found: 263.0912.

## Supplementary Material



## Data Availability

The data underlying this study are available in the published article and its Supporting Information.

## Notes

Detailed experimental procedures, analytical data, single-crystal X-ray data, and NMR spectra for all new compounds (PDF)
