# Supplementary material for: Ring Enlargements of in Situ-Formed Cyclopropanones by Sulfoxonium Ylides: One-Pot Synthesis of Alkylidene Cyclobutanones
Source: J Org Chem. 2026 Feb 16;91(8):3246–55. doi: 10.1021/acs.joc.5c03024 (PMC12954751; doi:10.1021/acs.joc.5c03024)
Supplement: Supplementary file 1 [file jo5c03024_si_001.pdf]

Supporting Information

Ring-Enlargements of in situ Formed Cyclopropanones by Sulfoxonium Ylides: One-Pot Synthesis of Alkylidene Cyclobutanones

Ishika Agrawal, Pedram Kalvani, Daniel B. Werz*

Albert-Ludwigs-Universität Freiburg

Institute of Organic Chemistry

Alberstraße 21, 79104 Freiburg, Germany

(*corresponding author: daniel.werz@chemie.uni-freiburg.de)

Table of Contents

General Experimental S2

Synthesis of Starting Materials S3

NMR Spectra of Sulfoxonium Ylides 2l-n S5

NMR Spectra of Alkylidene Cyclobutanones 3 .... S9

Follow-up Reactions: NMR spectra S34

Stereoselectivity Experiments S38

Optimization of Reaction Conditions S46

Single-Crystal X-Ray Diffraction S48

References S51

General Experimental

Air- and moisture-sensitive reactions were conducted in oven-dried or flame-dried glassware, septum-capped under atmospheric pressure of argon. Solvents used for column chromatography were distilled prior to use. Anhydrous solvents were obtained either from a solvent purification system (CH_2Cl_2, Et_2O, THF, MeCN, Toluene) and stored in flame-dried flasks over activated (4 Å) molecular sieves under argon or were purchased (Thermo Scientific) anhydrous over molecular sieves (MeOH, EtOH, acetone, DMF, $CHCl_3$, CH_2Cl_2, EtOAc, pyridine, 1,4-dioxane). Commercially available compounds were used without further purification unless specified otherwise.

For all purifications by flash column chromatography, Silica 60 (40 – 63 µm pore size) from Macherey-Nagel was used. Thin-layer chromatography was carried out on Silica gel 60 F_{254} aluminum sheets from Merck. For the detection of spots, UV light at 254 nm was used.

NMR spectra for proton (^1H), carbon (^{13}C), and fluorine (^{19}F) were recorded on Bruker instruments at Bruker Ascend 700 MHz, Prodigy, Avance Neo or Bruker 500 MHz Avance III HD or Bruker Ascend 400 MHz Avance Neo or Bruker 300 MHz Avance III HD Nano using the residual signals from $CDCl_3$, δ = 7.26 ppm and δ = 77.16 ppm as internal reference for ^1H and ^{13}C chemical shifts, respectively. Additionally, tetramethylsilane (TMS; δ = 0.00 ppm; 0.03%) was added to NMR samples. The following abbreviations denote multiplicities in ^1H, ^{13}C, and ^{19}F NMR chemical shifts: s = singlet, d = doublet, t = triplet, q = quartet, m = multiplet. The chemical shifts δ were reported in parts per million (ppm).

High-resolution mass spectroscopy (ESI-HRMS and APCI-HRMS) was carried out on a Thermo Scientific Exactive Orbitrap instrument. ATR-FTIR spectroscopy was carried out on a Spectrum Two FT-IR Spectrometer from Perkin Elmer, with samples measured neat on a diamond ATR crystal. Transmission bands were reported in cm^{-1}. Optical rotations were determined with a PERKIN ELMER 241 polarimeter at 598 nm. Data are reported as follows $[\alpha]_D^{temp}$, concentration (c in g/100 mL), and solvent. Melting points of solid products were determined using the open capillary method on a Schorpp MPM-HV2 apparatus. Specific reaction conditions are given in the following procedures.

Enantiomeric excess was determined by HPLC on chiral stationary phases using Chiralcel OD-3 or Chiralpak AD-3 columns (3 µm; 150 × 4.6 mm). Eluent systems included *n*-heptane/*i*-PrOH (95:5) with 0.1% AcOH, *n*-heptane/i-PrOH (98:2), *n*-heptane/EtOH (85:15), and *n*-heptane/EtOH (80:20) mixtures. Detection was performed at 218–288 nm using Agilent 1260/1290 Infinity II systems equipped with DAD or VWD detectors.

Synthesis of Starting Materials

Synthesis of 1-Tosylcyclopropan-1-ol (SCP)(1a)

1-Tosylcyclopropan-1-ol (**1a**) was prepared according to a slightly modified procedure previously reported by Lindsay and co-workers.[1]

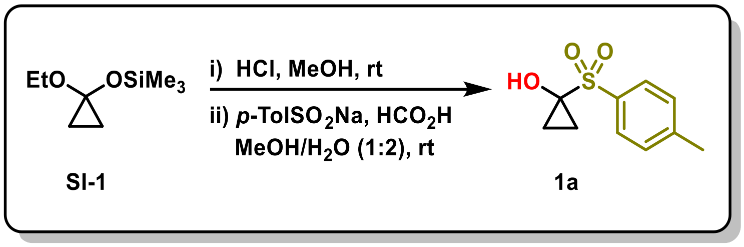

(1-Ethoxycycloproxy)trimethylsilane (**SI-1**, 2.00 g, 11.5 mmol, 1.00 eq.) was dissolved in anhydrous MeOH (6.0 mL, 2.0 M), aq. HCl (37%, 2 drops) was added and the reaction mixture was stirred at room temperature for 1.5 h. Subsequently, H_2O (12 mL) sodium *p*-toluenesulfinate (4.10 g, 23.0 mmol, 2.00 eq.) and formic acid (5.30 g, 114 mmol, 4.00 mL, 9.90 eq.) were added and the mixture was stirred for 48 h at room temperature. EtOAc (100 mL) was added and the org. phase was washed with H_2O (3 x 100 mL). Sat. aq. $NaHCO_3$ solution (100 mL) was added to the combined aq. phases which were then extracted with EtOAc (100 mL). The combined org. phases were dried by stirring over anhydrous $MgSO_4$, filtered through a plug of cotton wool and the solvent was removed under reduced pressure to yield 1-tosylcyclopropan-1-ol (**1a**, 2.20 g, 10.5 mmol, 91%) as a colorless solid without further purification.

All analytical data were consistent with those reported in the literature.[1]

Synthesis of Substituted Sulphonyl Cyclopropanol (SCPs) (1b-h)

The reactions to synthesize substituted SCPs (**1b-h**) were carried out following literature procedures reported by Lindsay and co-workers.[1] All analytical data were consistent with those reported in the literature.[1]

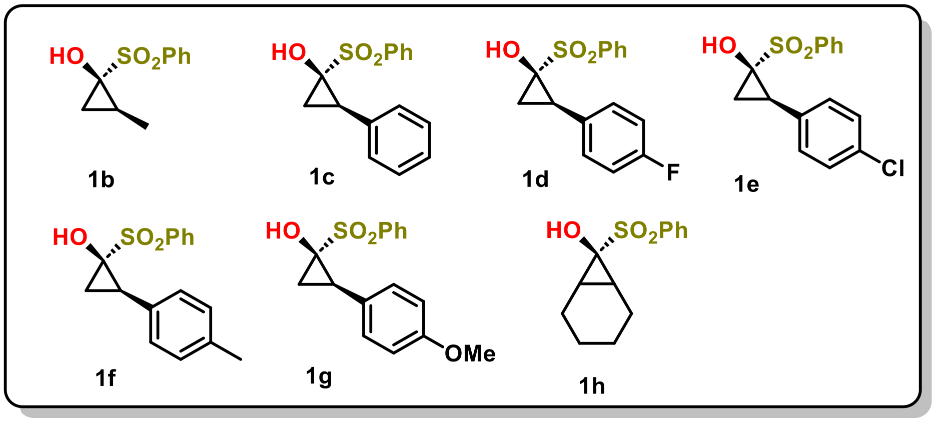

NMR Spectra of Sulfoxonium Ylides 2l-n

All analytical data for **2a-2k** were consistent with those reported in the literature.[2]

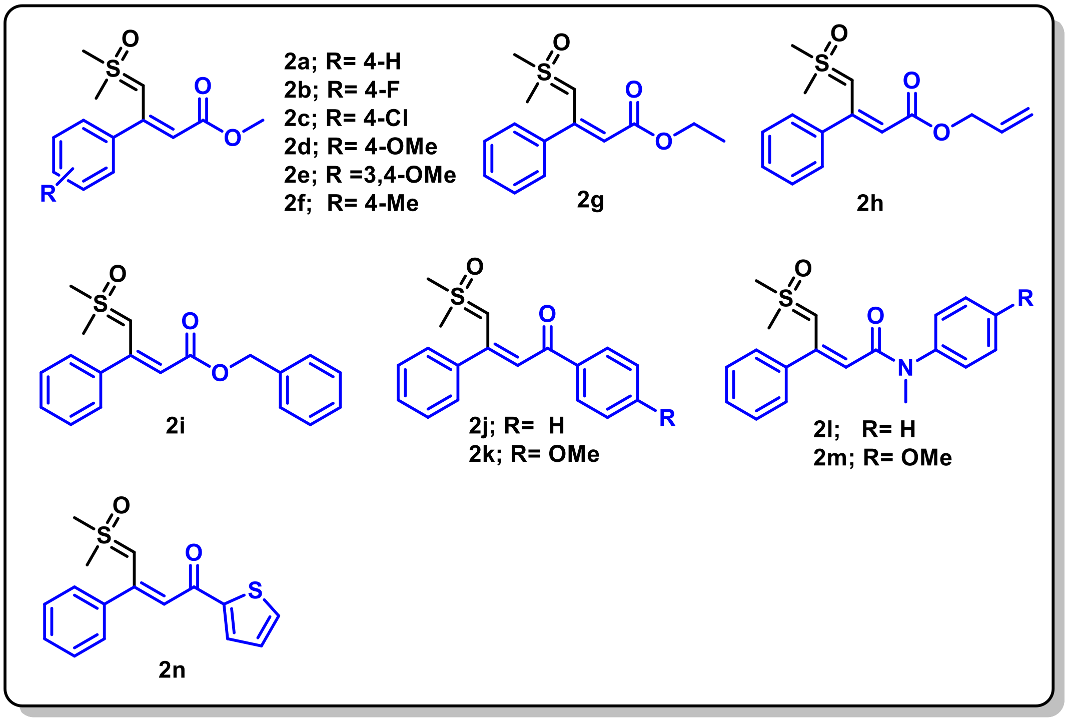

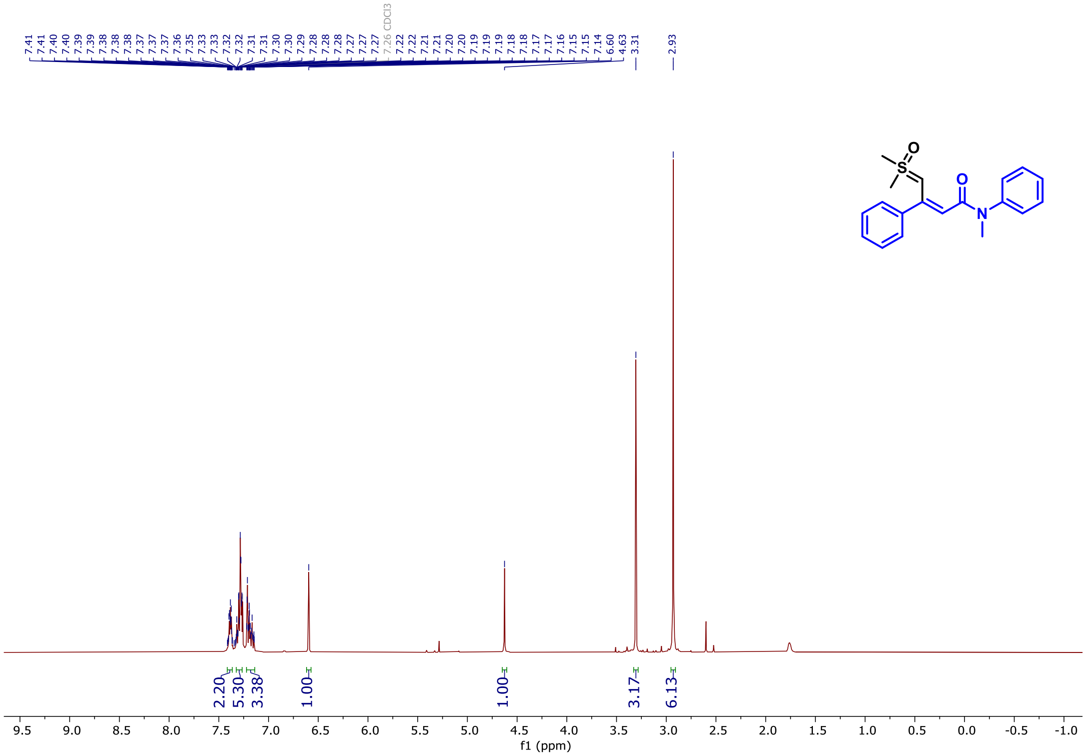

^{1}H-NMR (400 MHz, $CDCl_3$) of **2l**

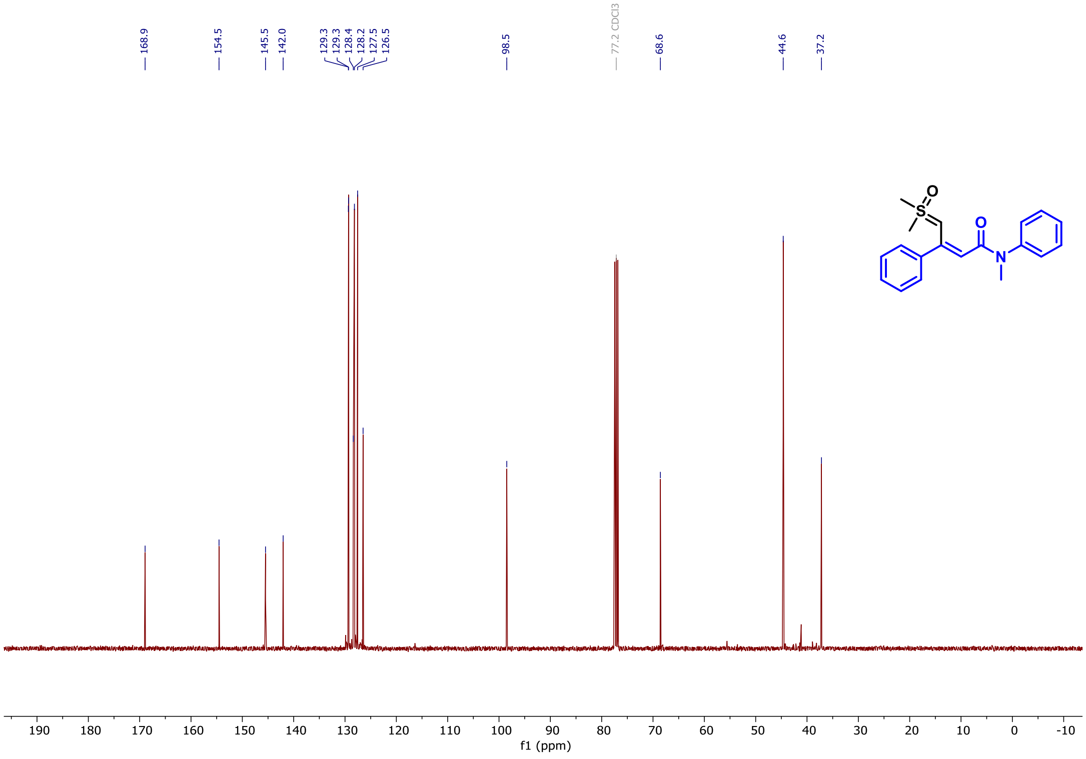

$^{13}C\{^1H\}$-NMR (101 MHz, $CDCl_3$) of **2l**

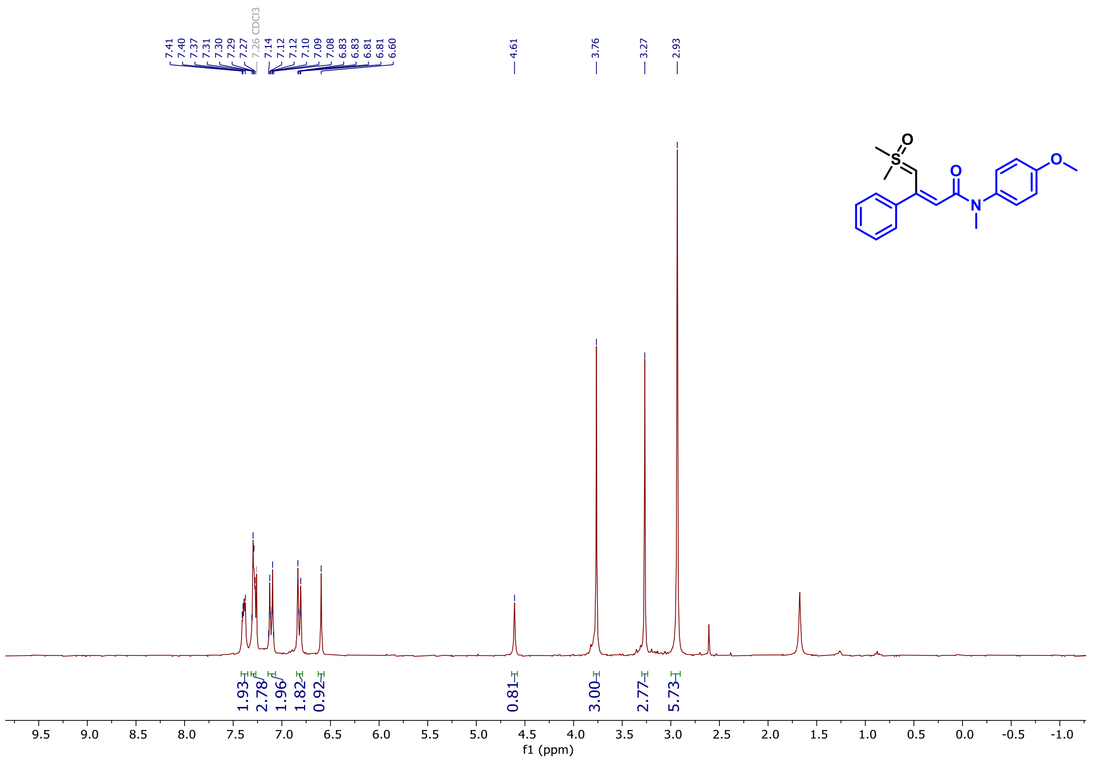

^{1}H-NMR (300 MHz, $CDCl_3$) of **2m**

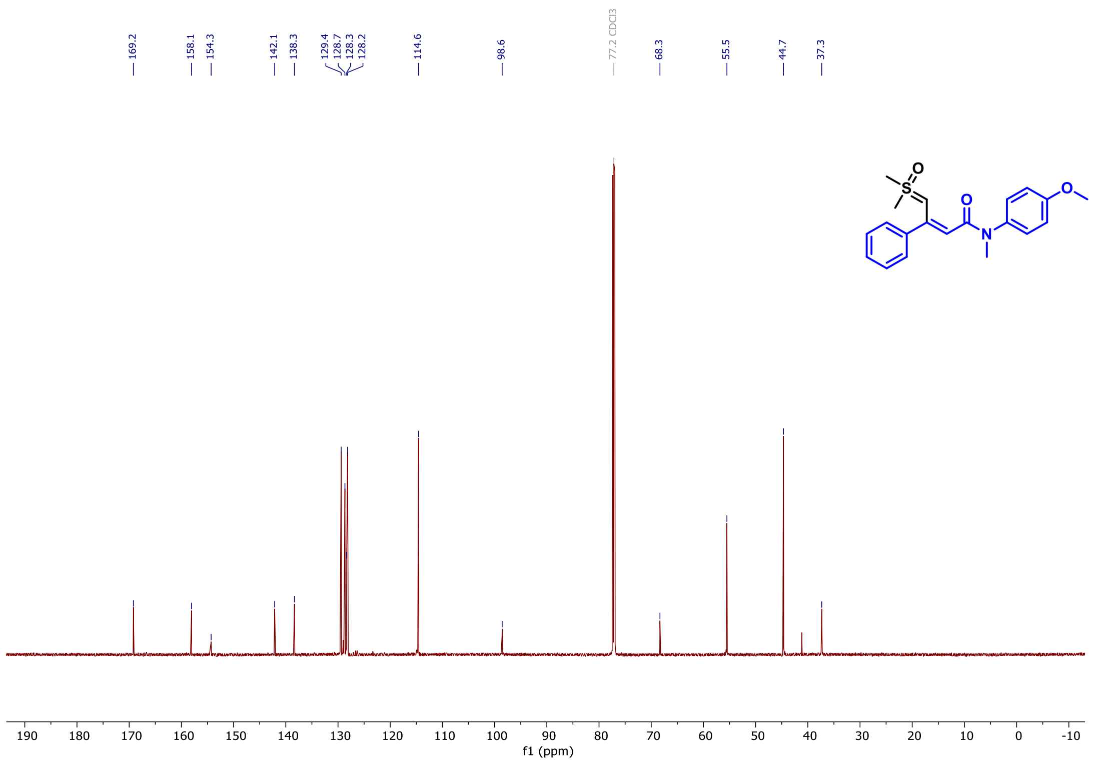

^{13}C{^{1}H}-NMR (176 MHz, $CDCl_3$) of **2m**

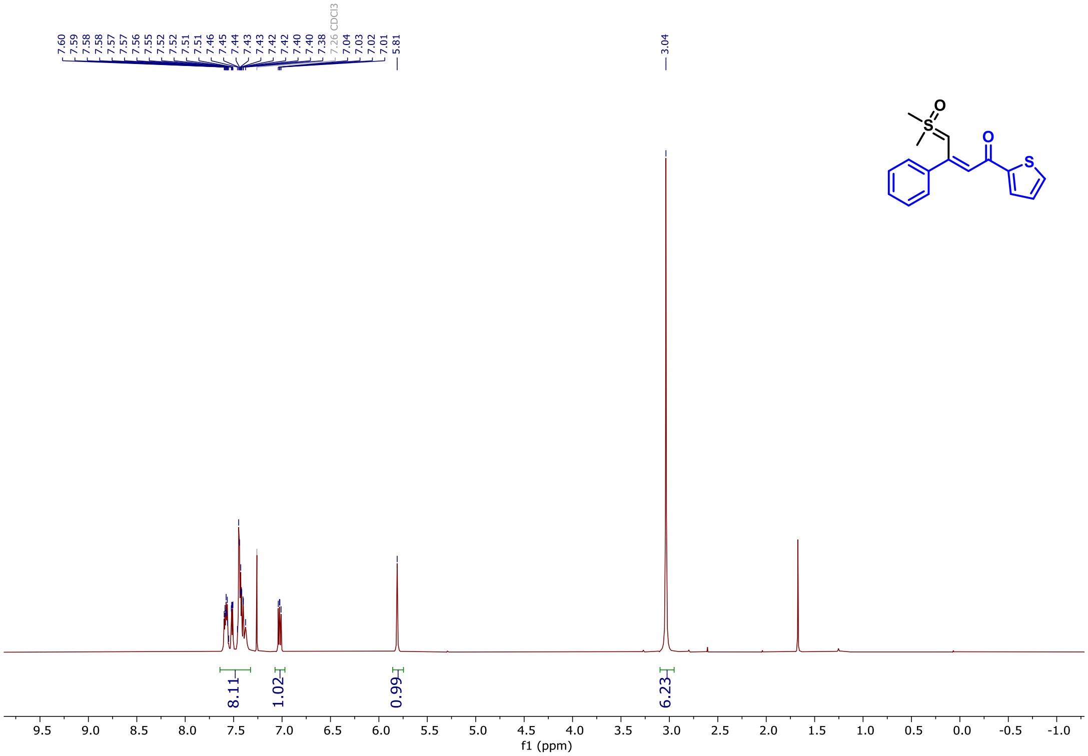

^{1}H-NMR (300 MHz, $CDCl_3$) of **2n**

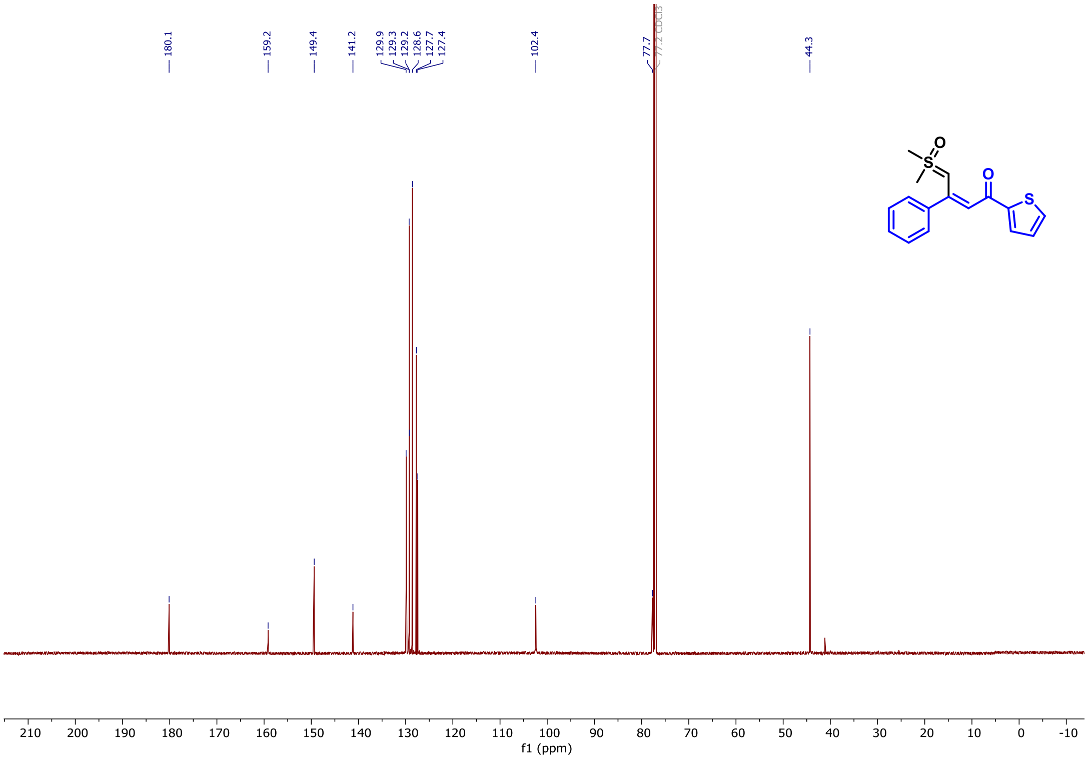

^{13}C{^{1}H}-NMR (176 MHz, $CDCl_3$) of **2n**

NMR Spectra of Alkylidene Cyclobutanones 3

Note: The reported *E*:*Z* ratio was determined from the crude mixture prior to flash chromatography, as indicated by ^{1}H NMR analysis. The NMR data correspond to the major diastereomer; only traces of the minor isomer were recovered after purification, suggesting that partial isomerization occurred during this process.

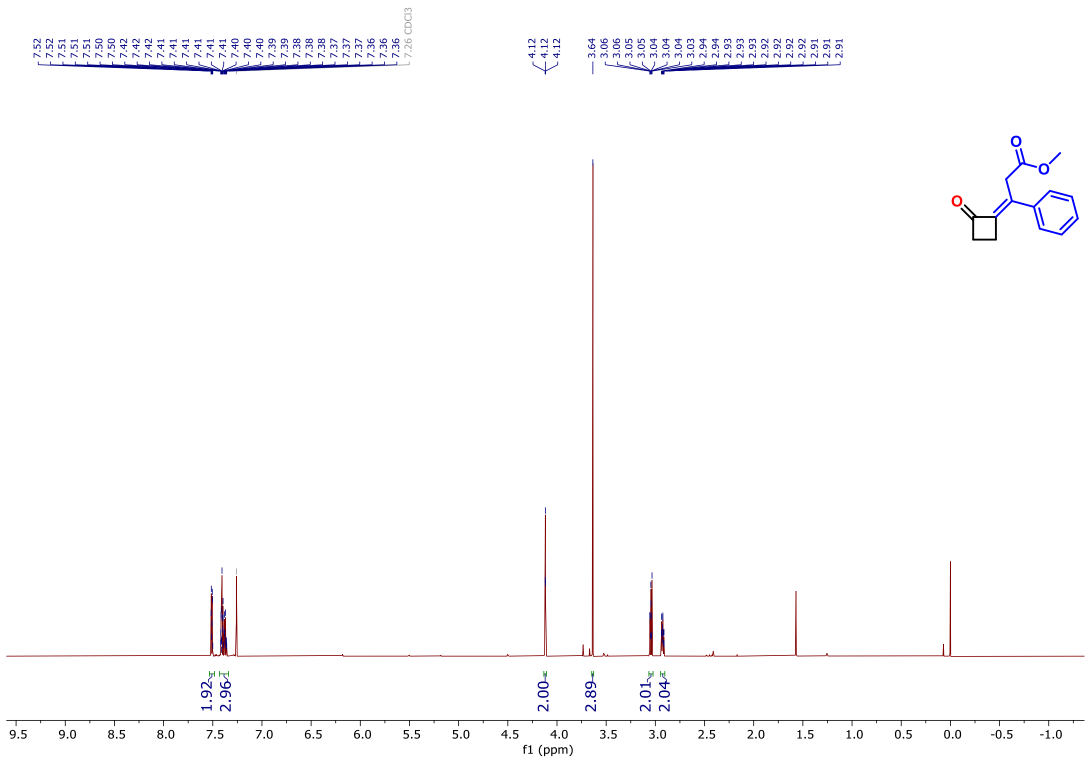

^{1}H-NMR (700 MHz, $CDCl_3$) of **3aa**

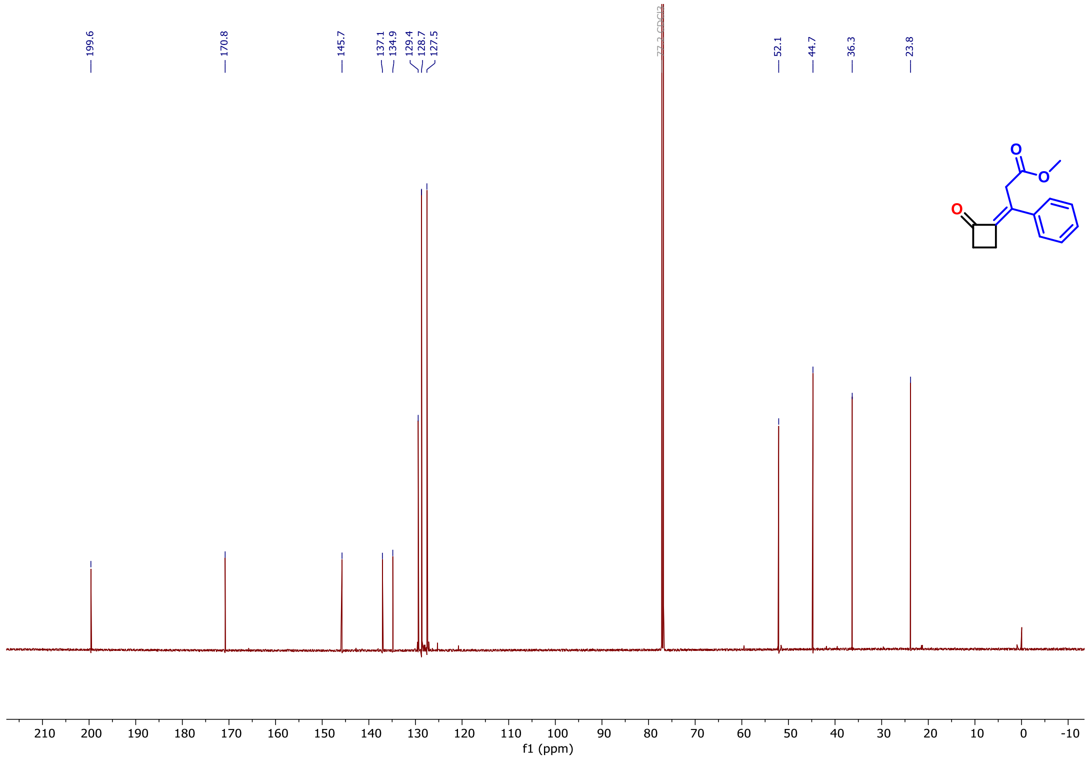

^{13}C{^{1}H}-NMR (176 MHz, $CDCl_3$) of **3aa**

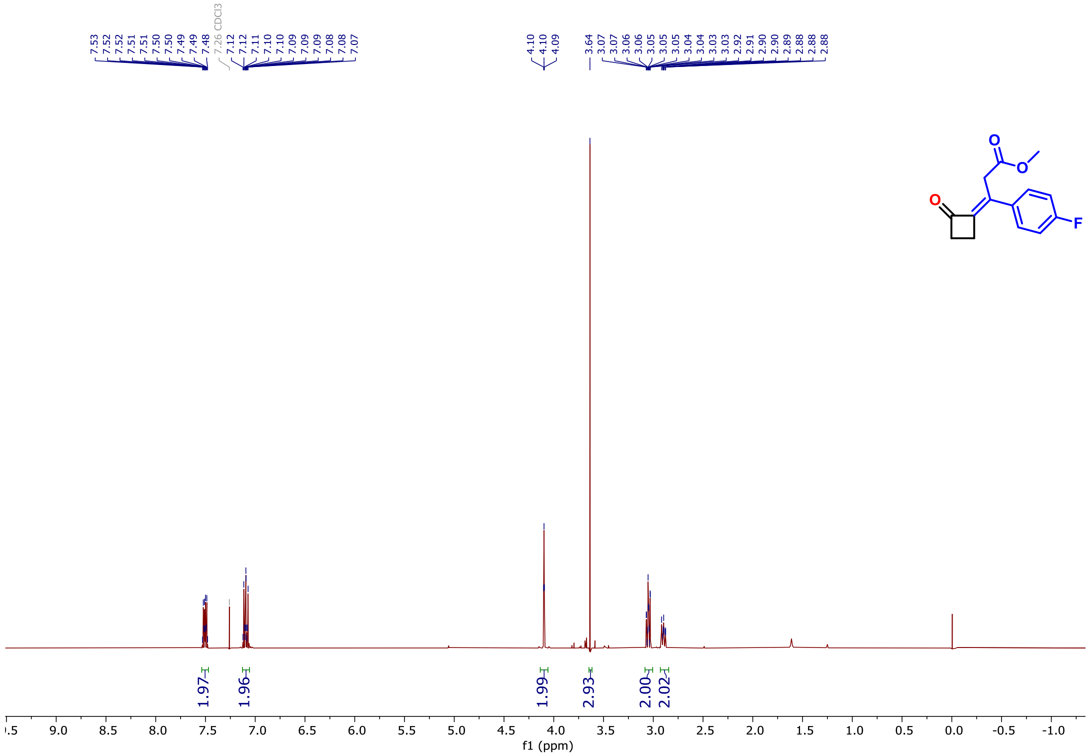

^{1}H-NMR (400 MHz, $CDCl_3$) of **3ab**

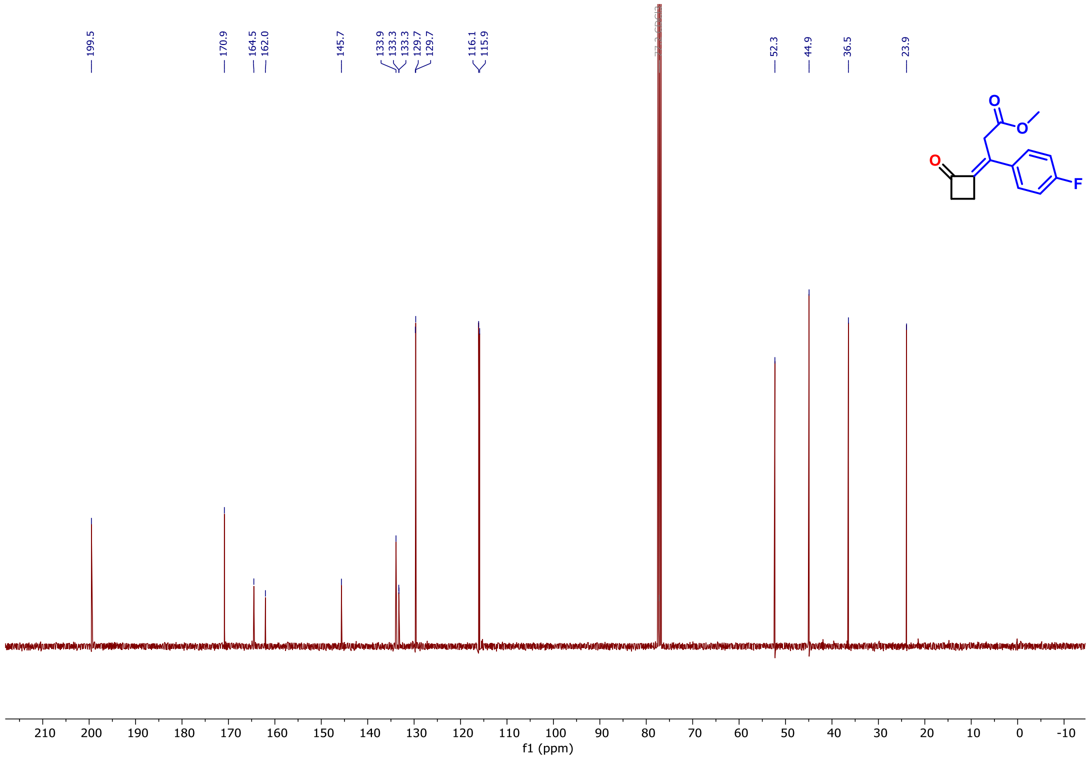

^{13}C{^{1}H}-NMR (101 MHz, $CDCl_3$) of **3ab**

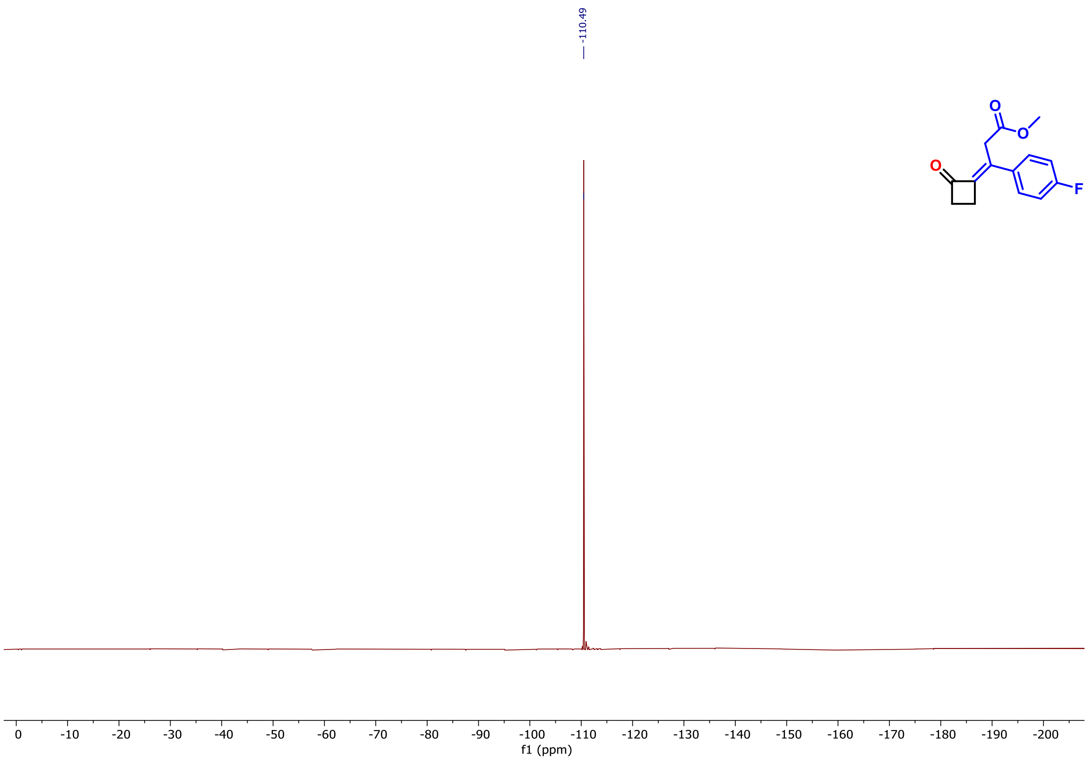

^{19}F-NMR (377 MHz, $CDCl_3$) of **3ab**

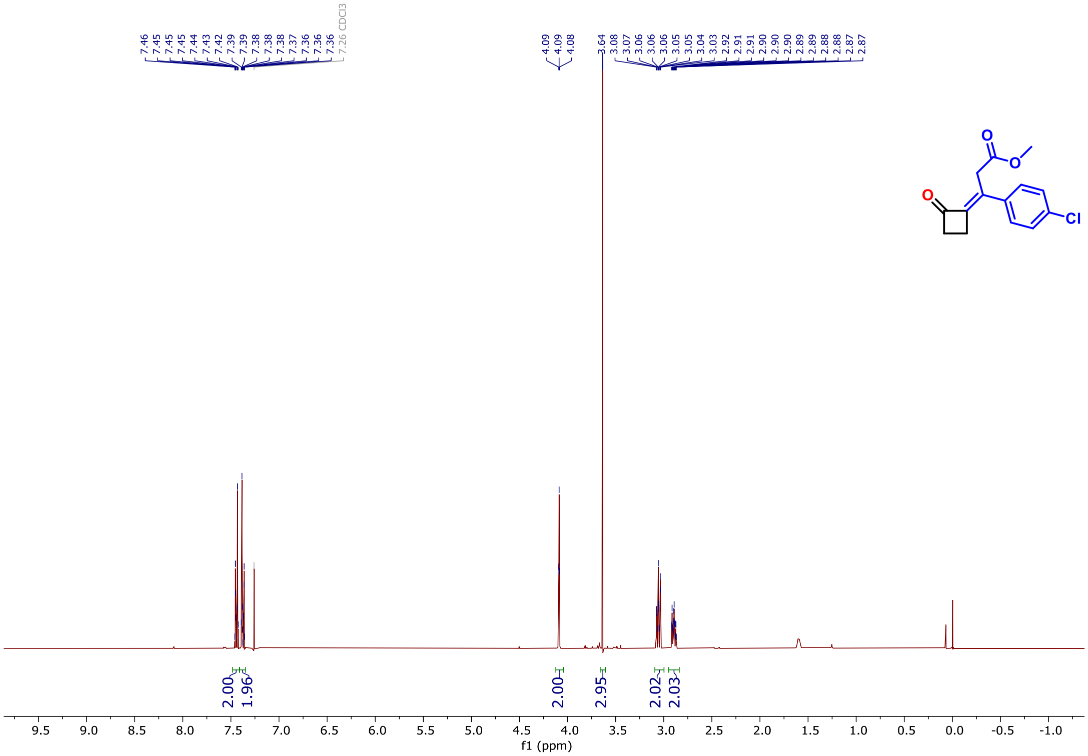

^{1}H-NMR (400 MHz, $CDCl_3$) of **3ac**

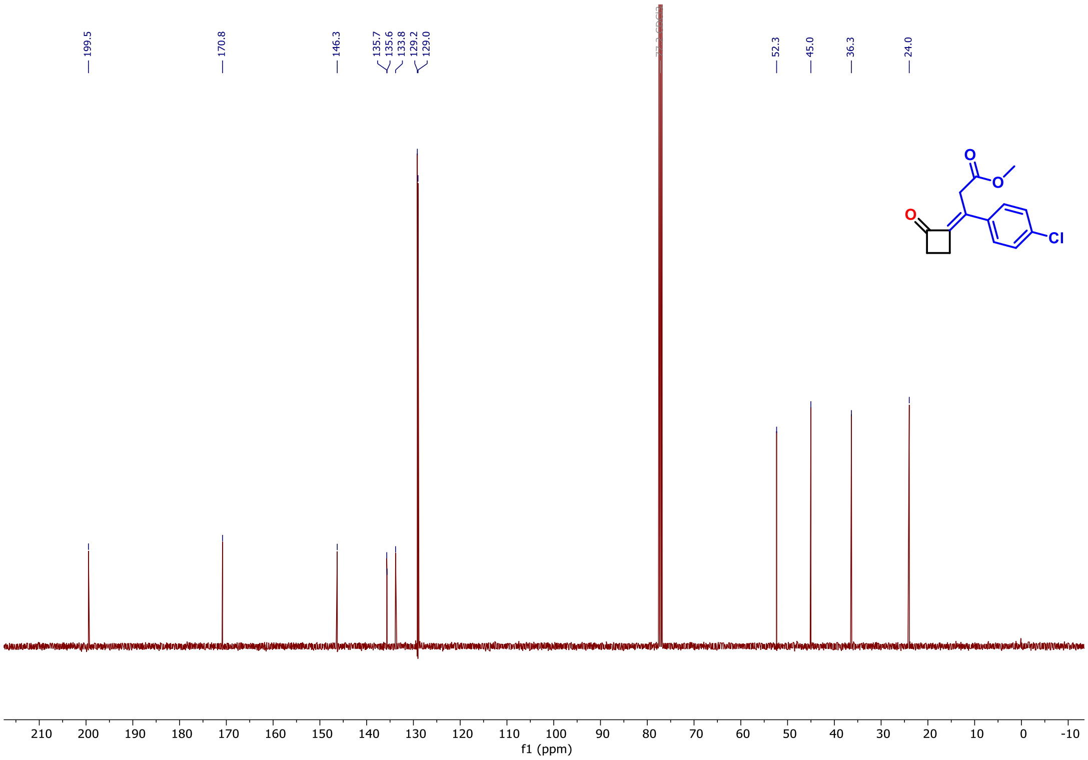

^{13}C{^{1}H}-NMR (101 MHz, $CDCl_3$) of **3ac**

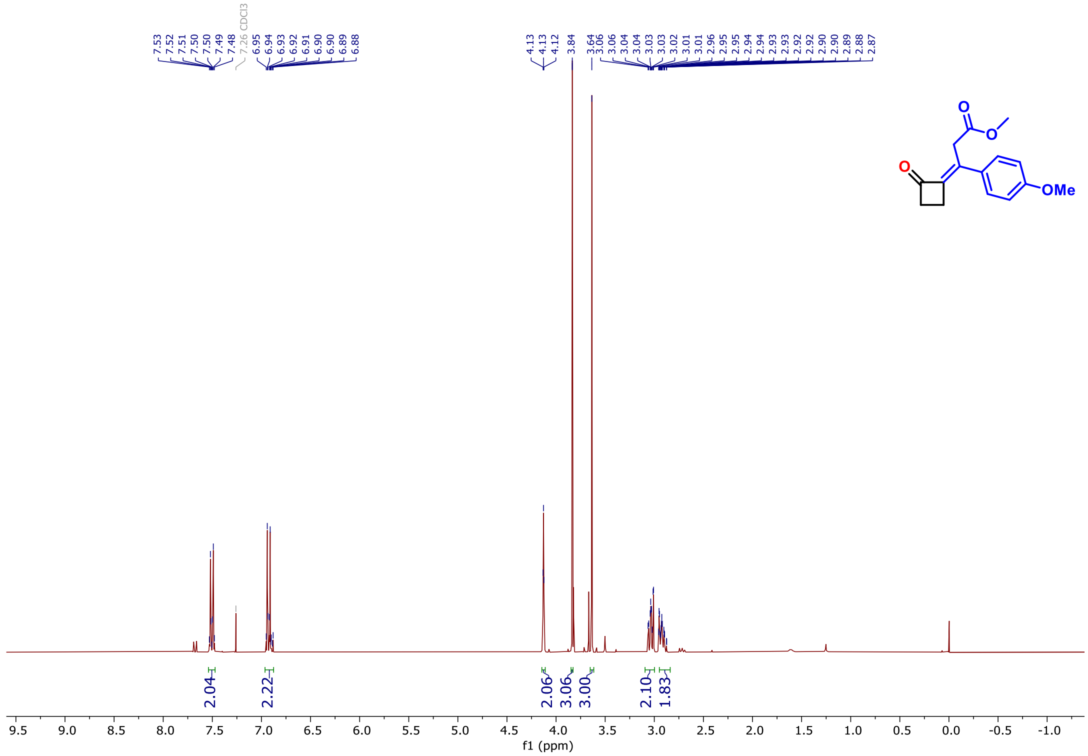

¹H-NMR (300 MHz, $CDCl_3$) of **3ad**

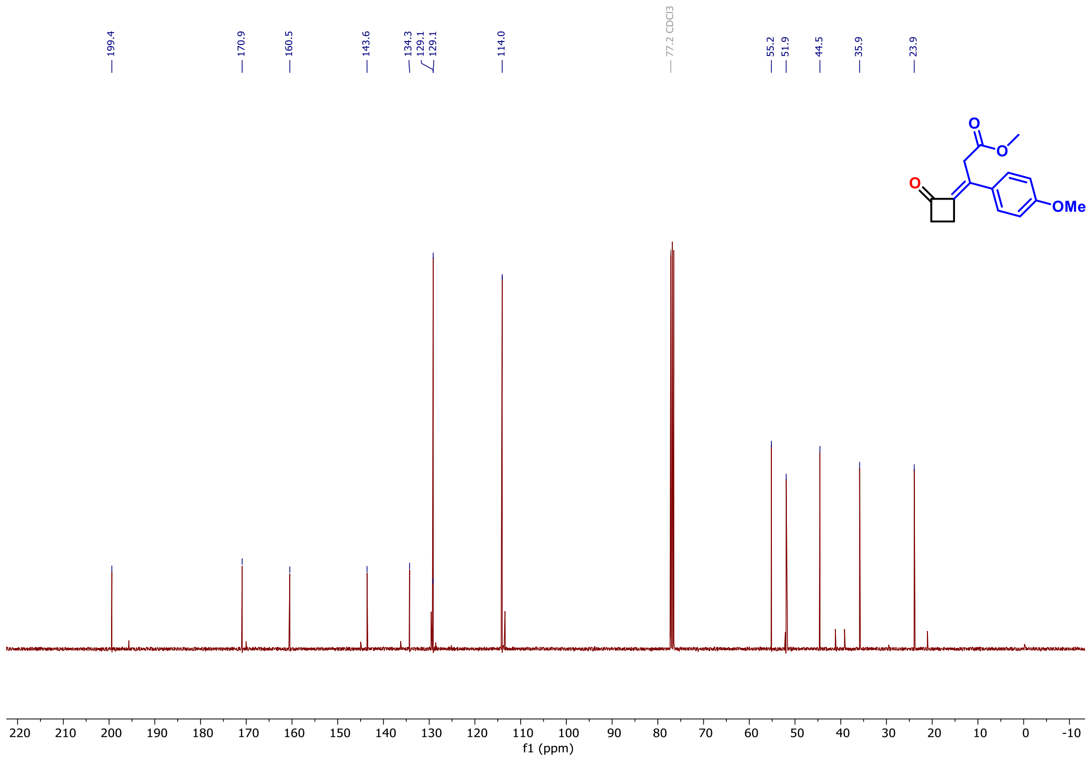

¹³C{¹H}-NMR (101 MHz, $CDCl_3$) of **3ad**

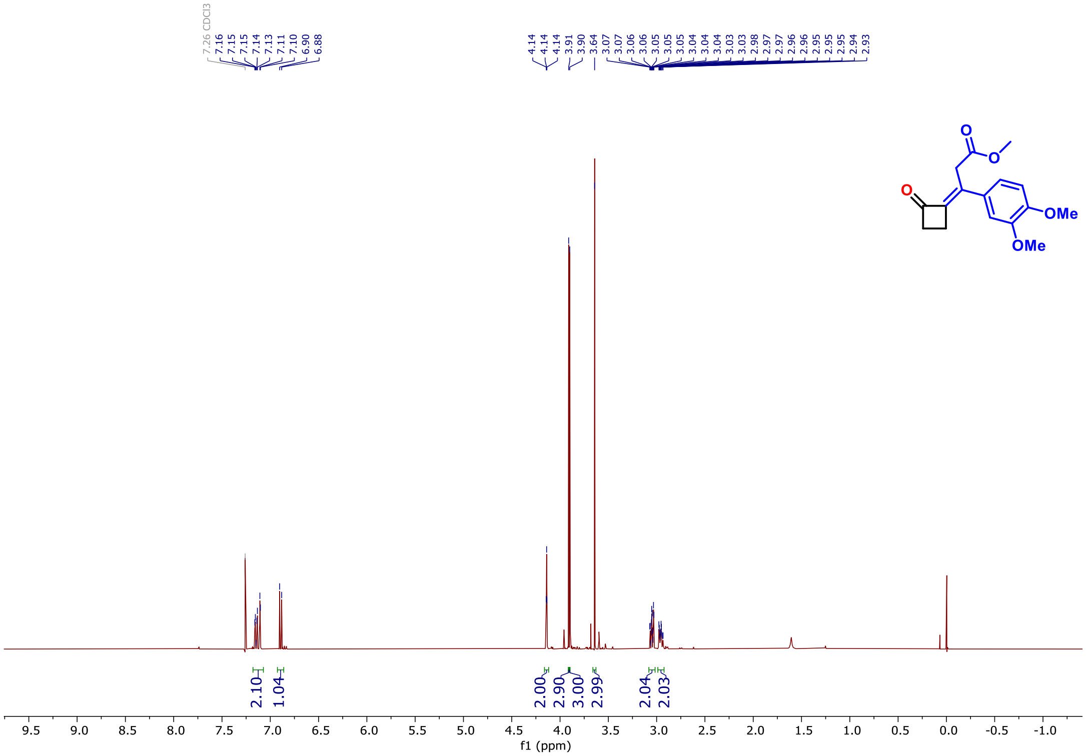

^{1}H-NMR (400 MHz, $CDCl_3$) of **3ae**

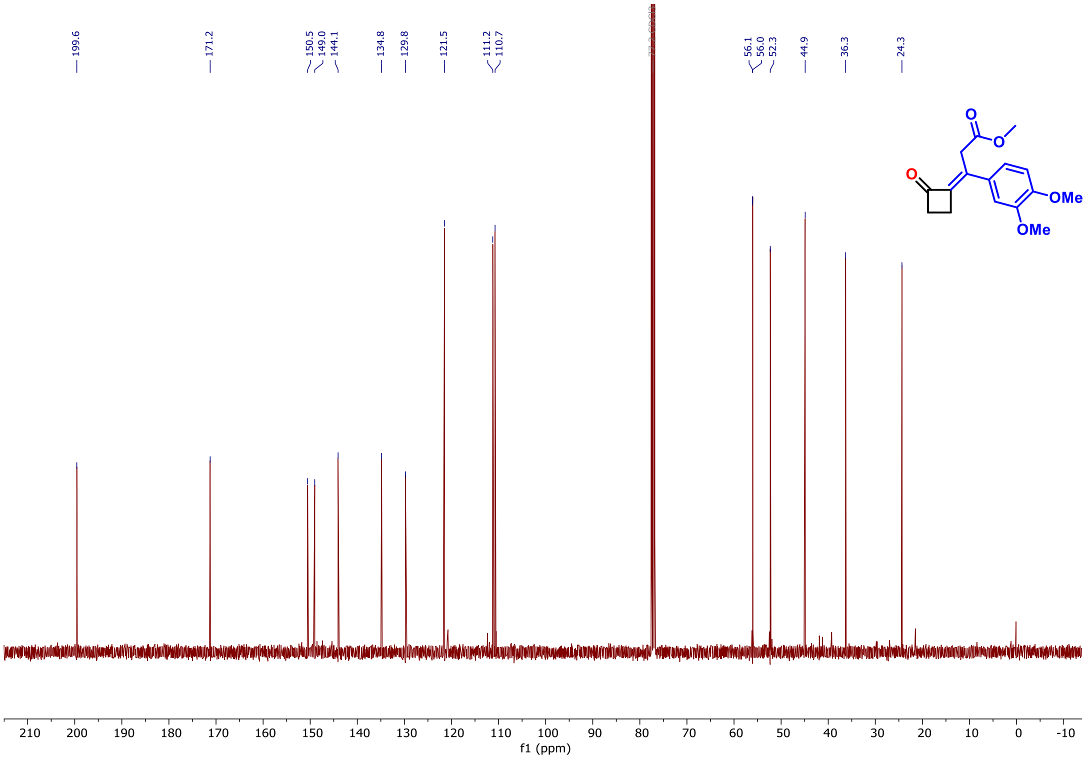

$^{13}C\{^{1}H\}$-NMR (101 MHz, $CDCl_3$) of **3ae**

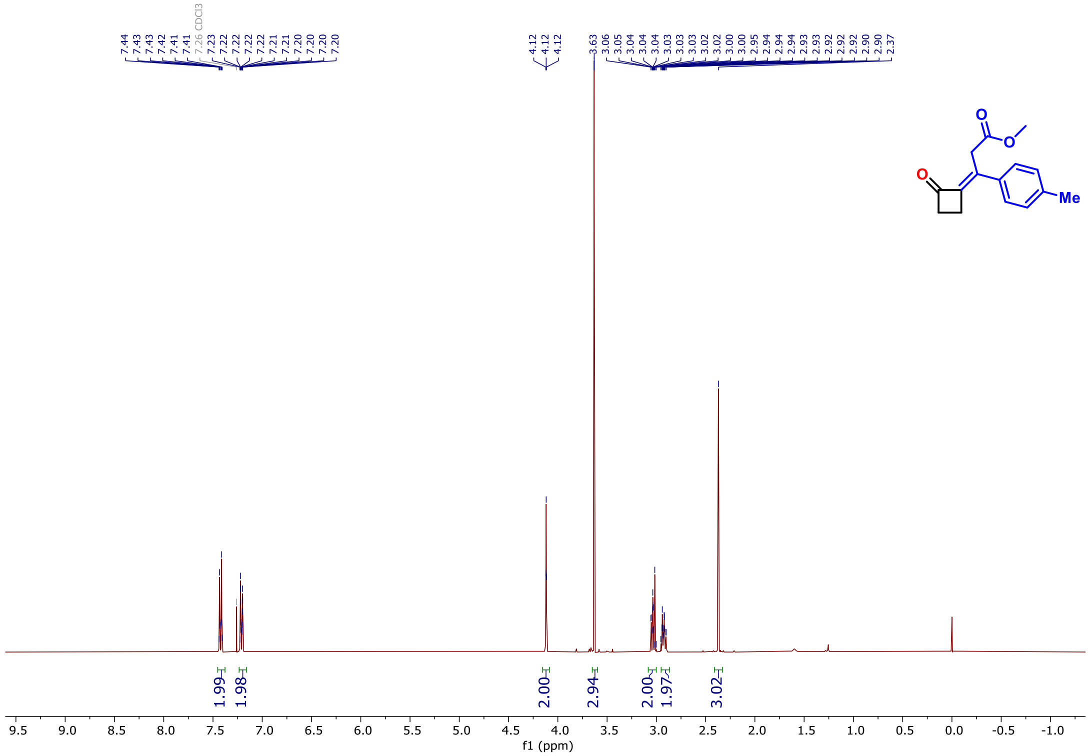

¹H-NMR (400 MHz, $CDCl_3$) of **3af**

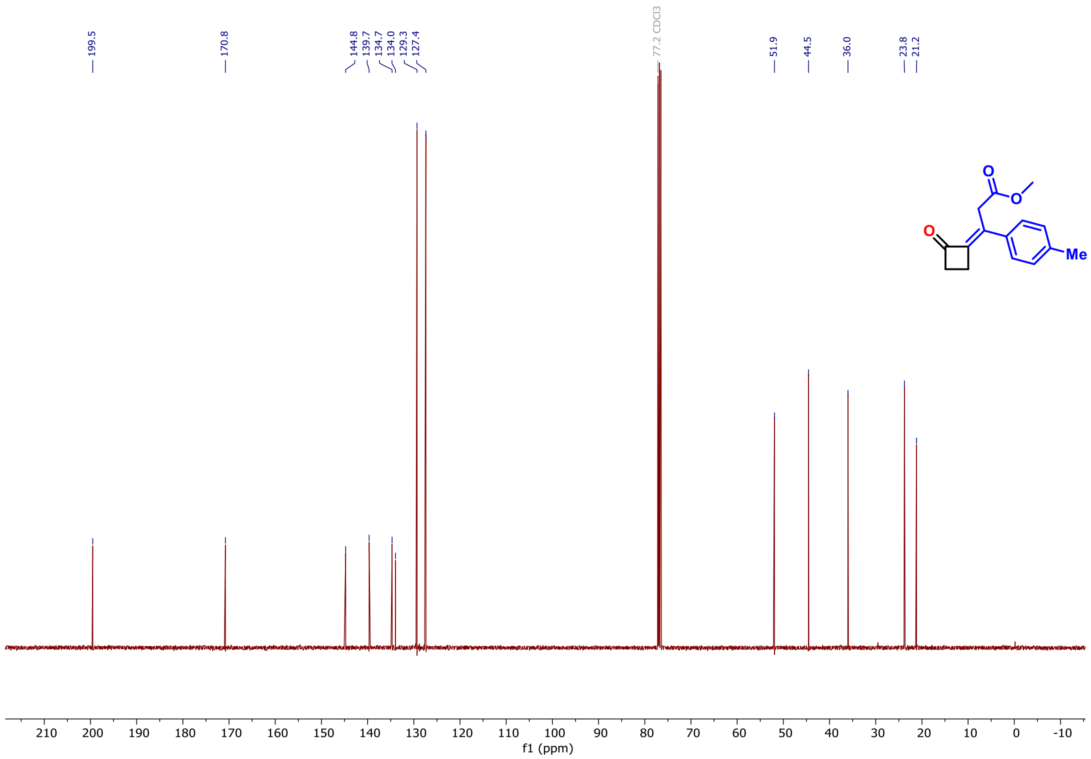

¹³C{¹H}-NMR (101 MHz, $CDCl_3$) of **3af**

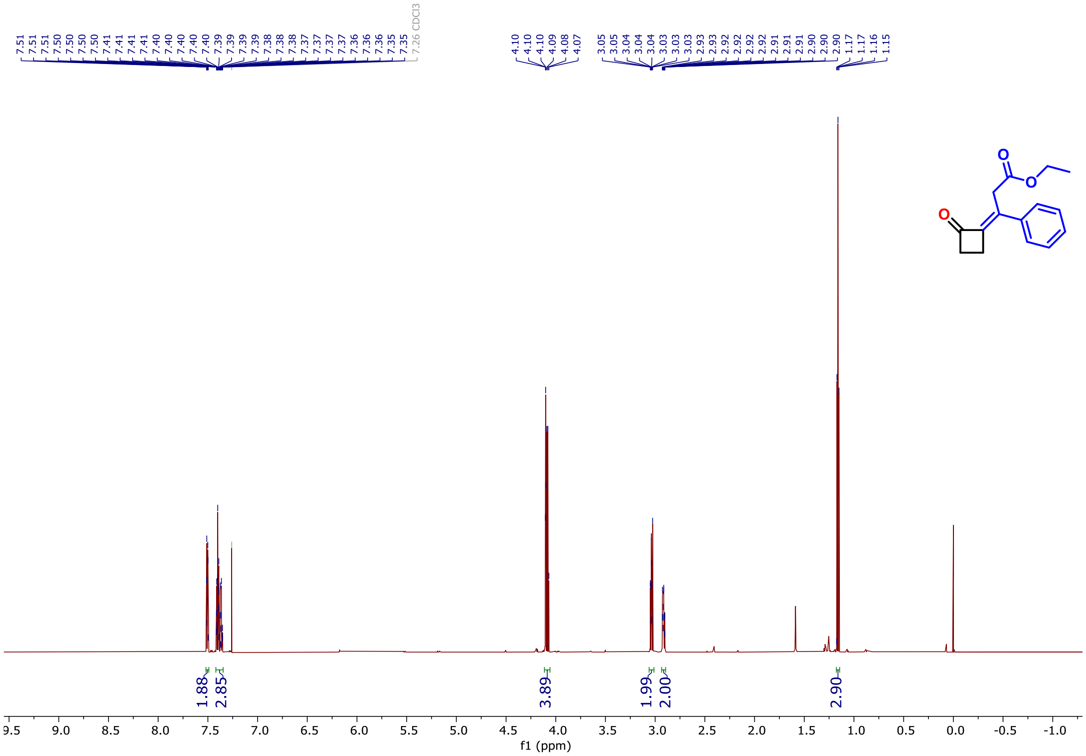

¹H-NMR (700 MHz, $CDCl_3$) of **3ag**

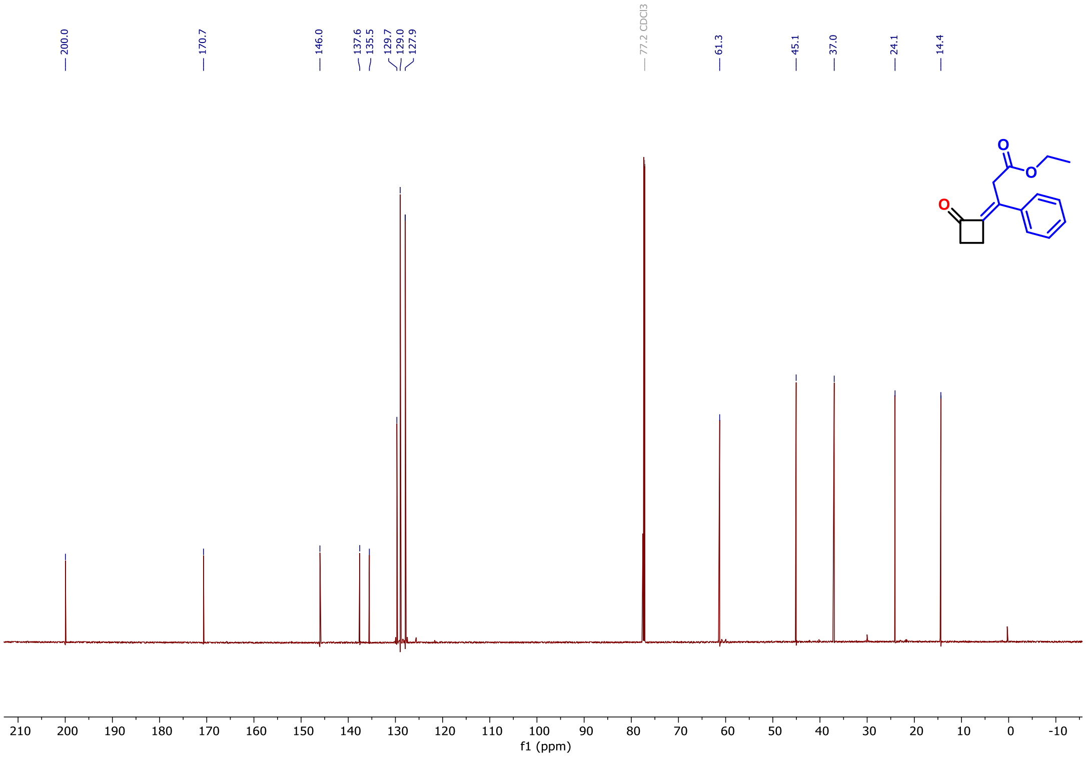

$^{13}C\{^1H\}$-NMR (176 MHz, $CDCl_3$) of **3ag**

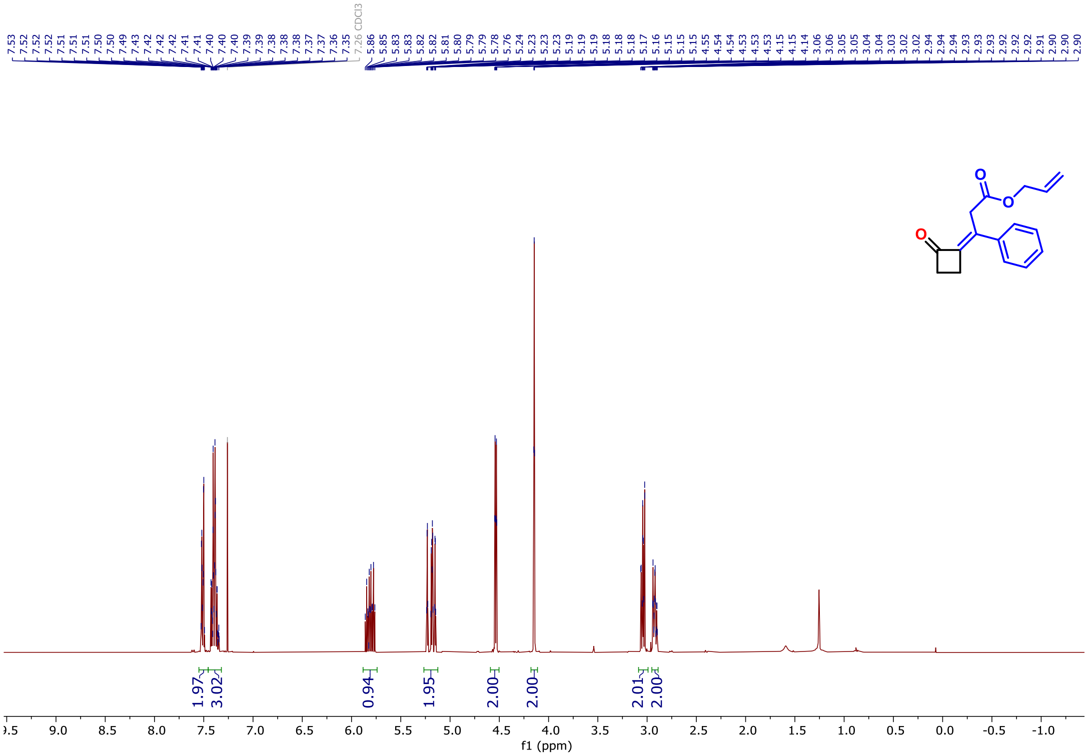

^{1}H-NMR (400 MHz, $CDCl_3$) of **3ah**

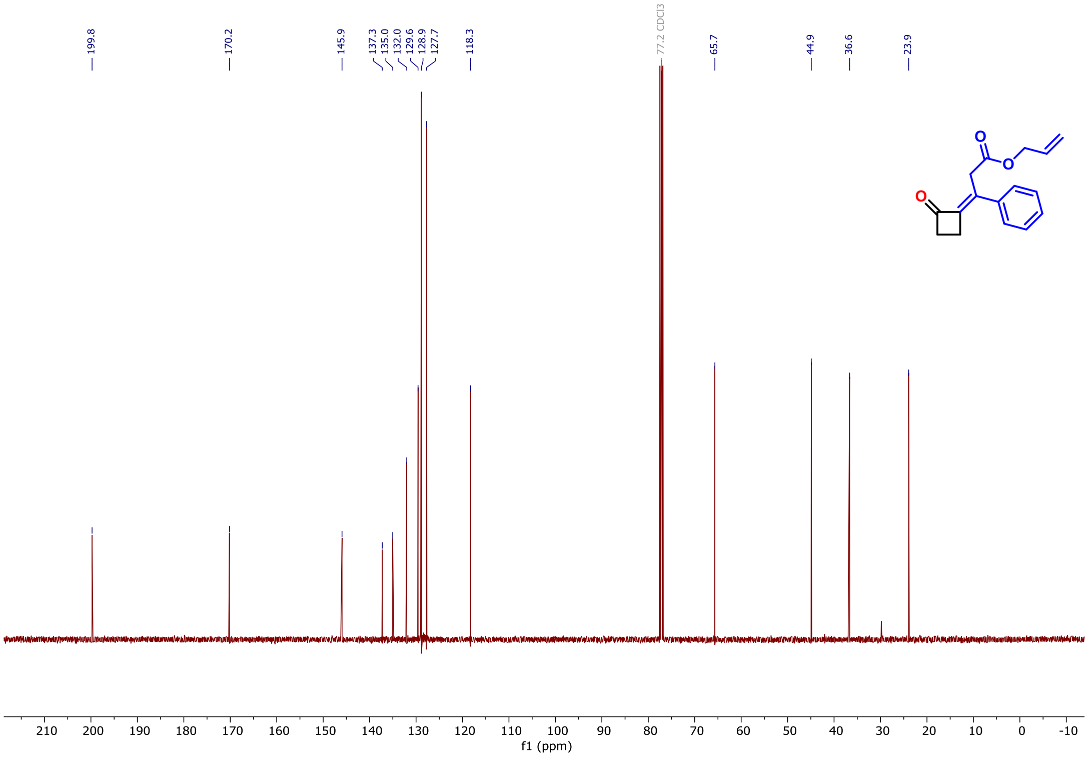

^{13}C{^{1}H}-NMR (101 MHz, $CDCl_3$) of **3ah**

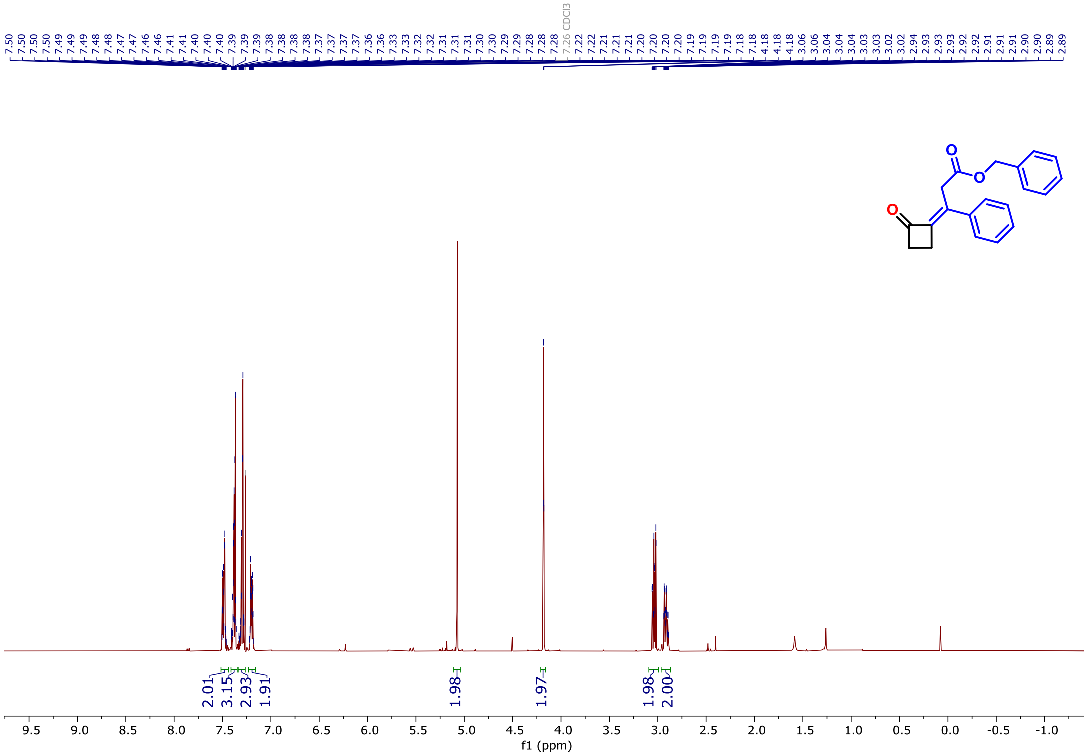

^{1}H-NMR (400 MHz, $CDCl_3$) of **3ai**

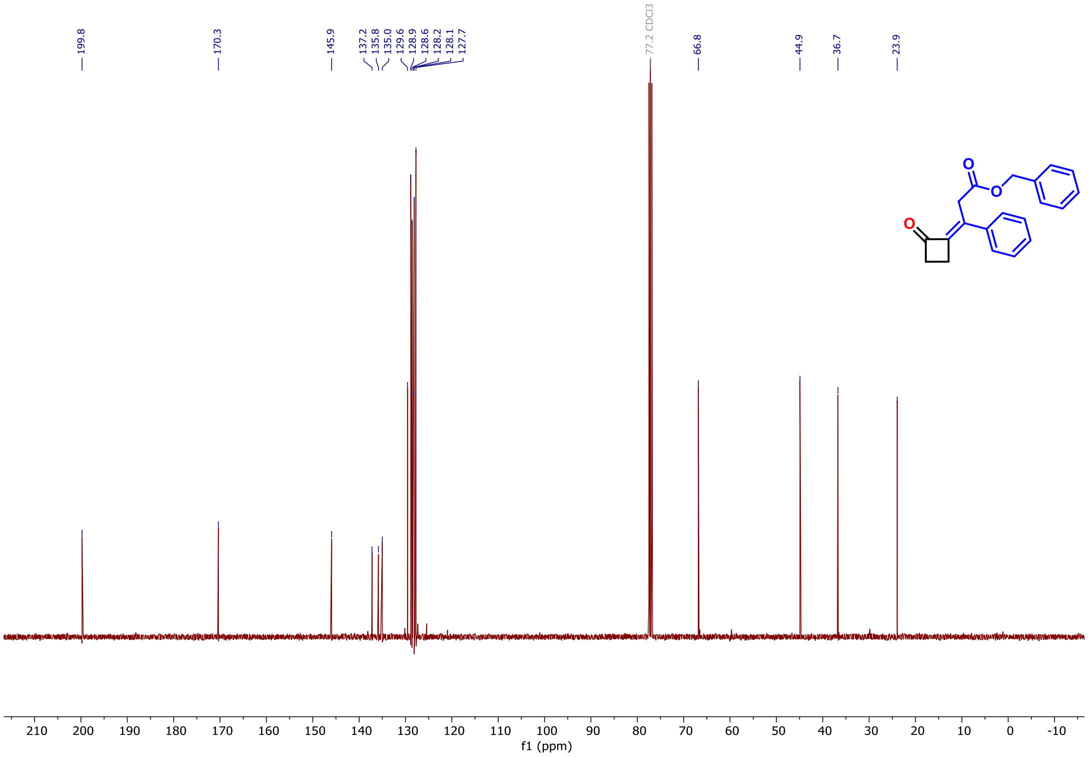

^{13}C{^{1}H}-NMR (101 MHz, $CDCl_3$) of **3ai**

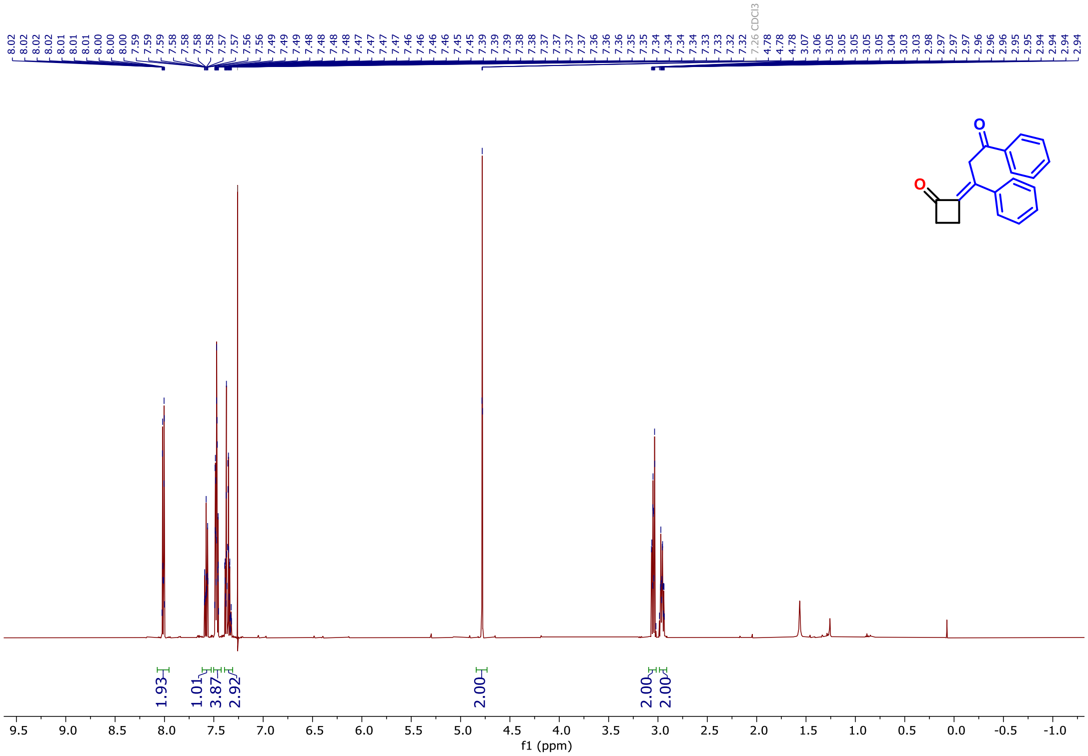

^{1}H-NMR (500 MHz, $CDCl_3$) of **3aj**

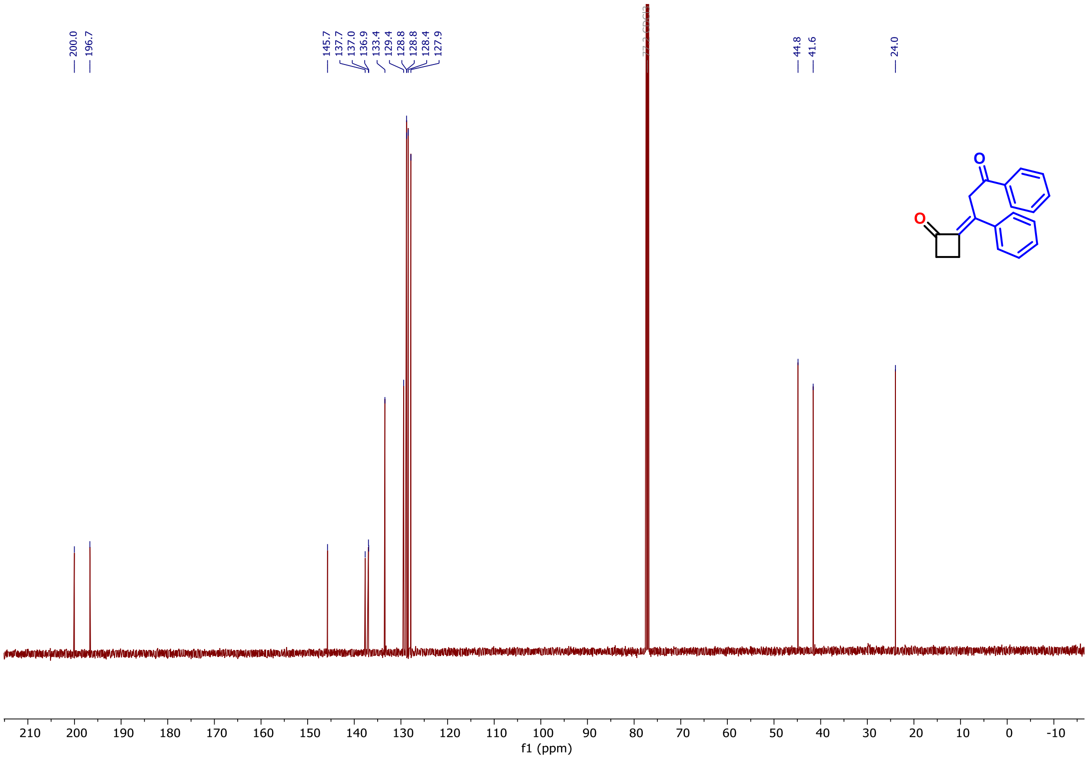

^{13}C{^{1}H}-NMR (126 MHz, $CDCl_3$) of **3aj**

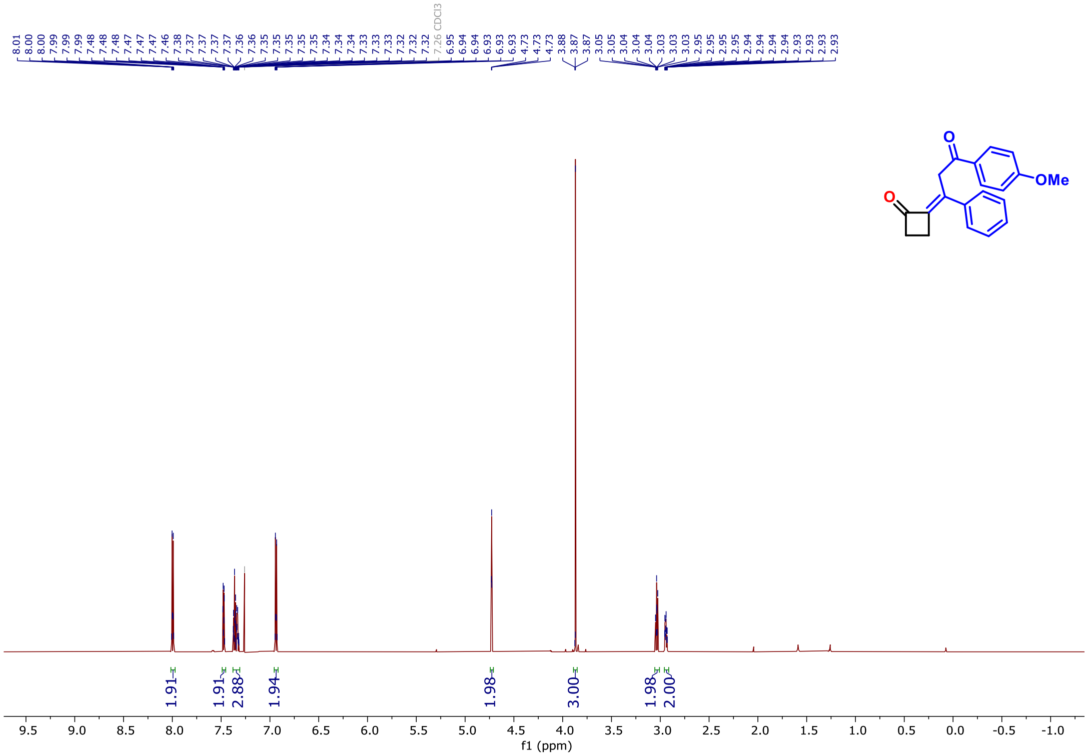

¹H-NMR (700 MHz, $CDCl_3$) of **3ak**

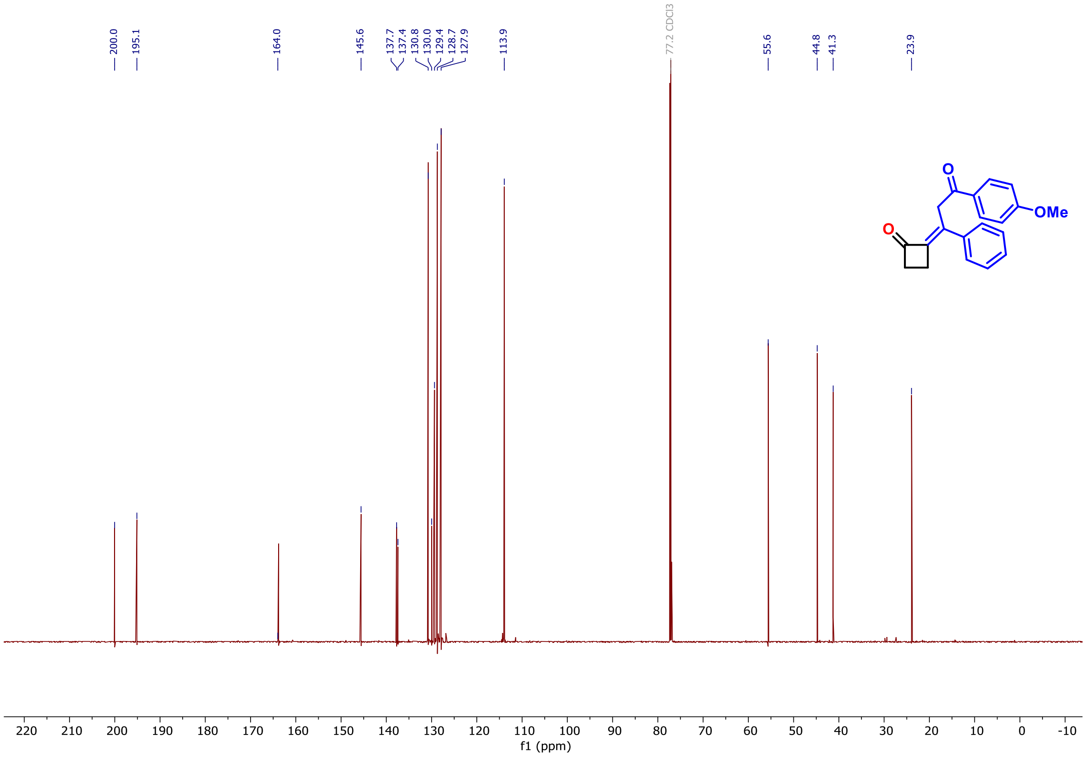

$^{13}C\{^1H\}$-NMR (176 MHz, $CDCl_3$) of **3ak**

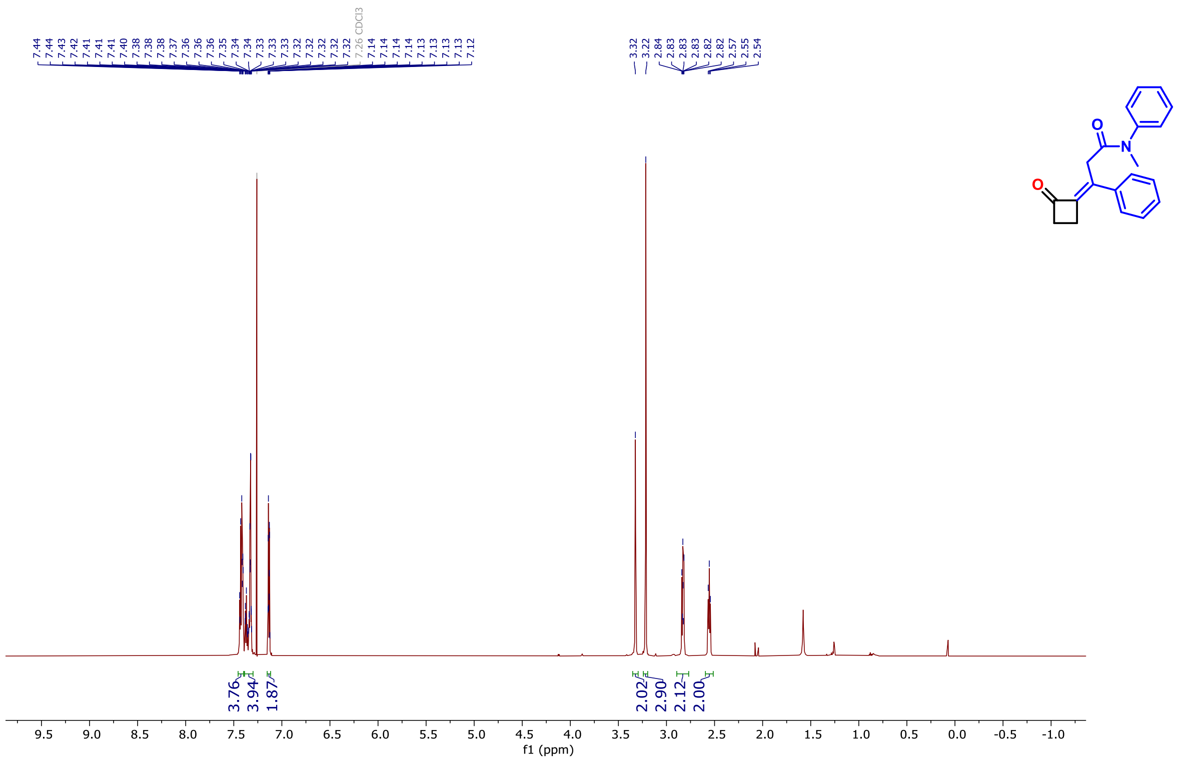

¹H-NMR (700 MHz, $CDCl_3$) of **3al**

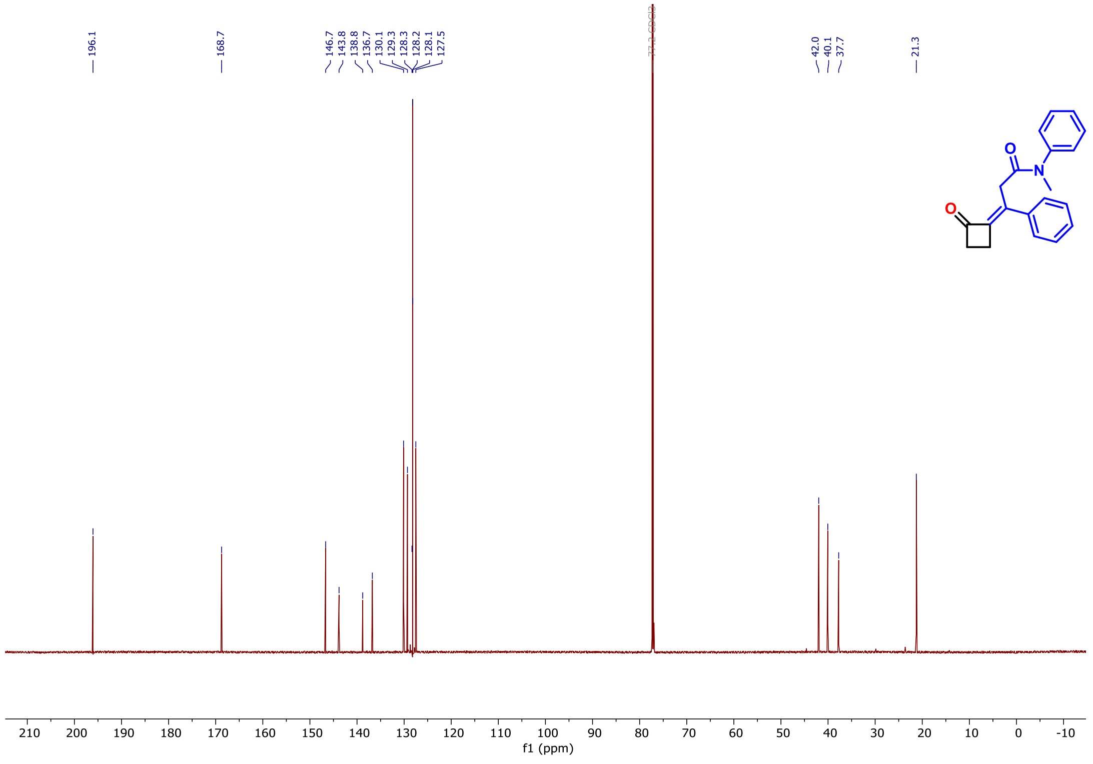

$^{13}C\{^1H\}$-NMR (176 MHz, $CDCl_3$) of **3al**

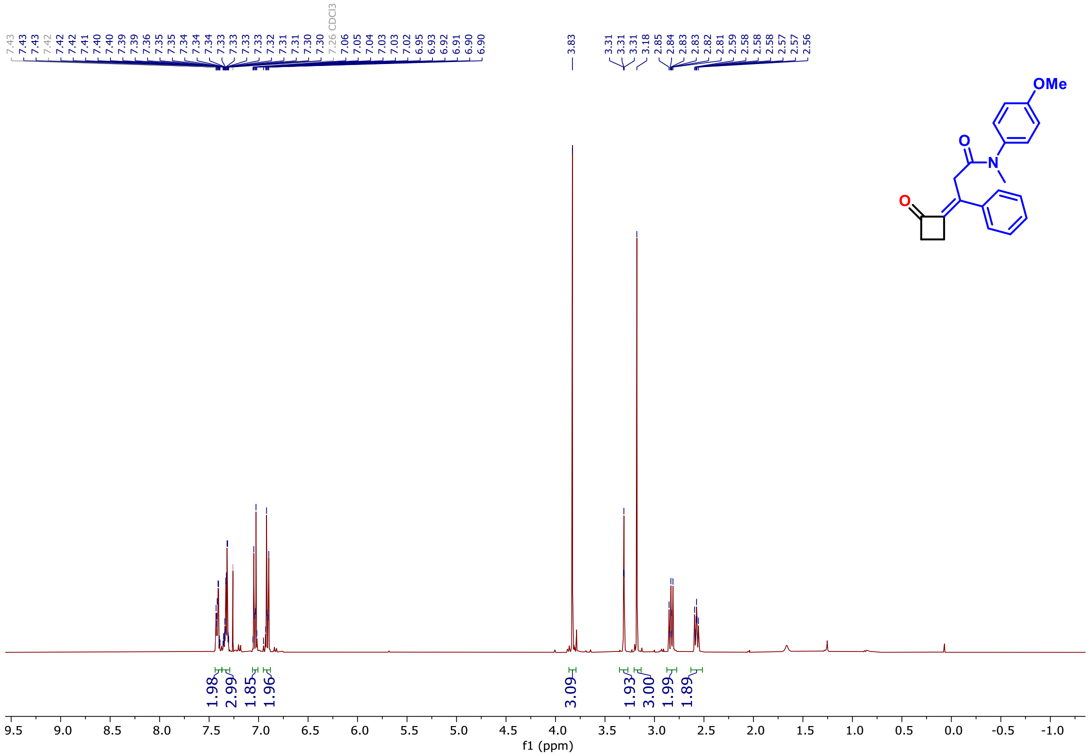

^{1}H-NMR (400 MHz, $CDCl_3$) of **3am**

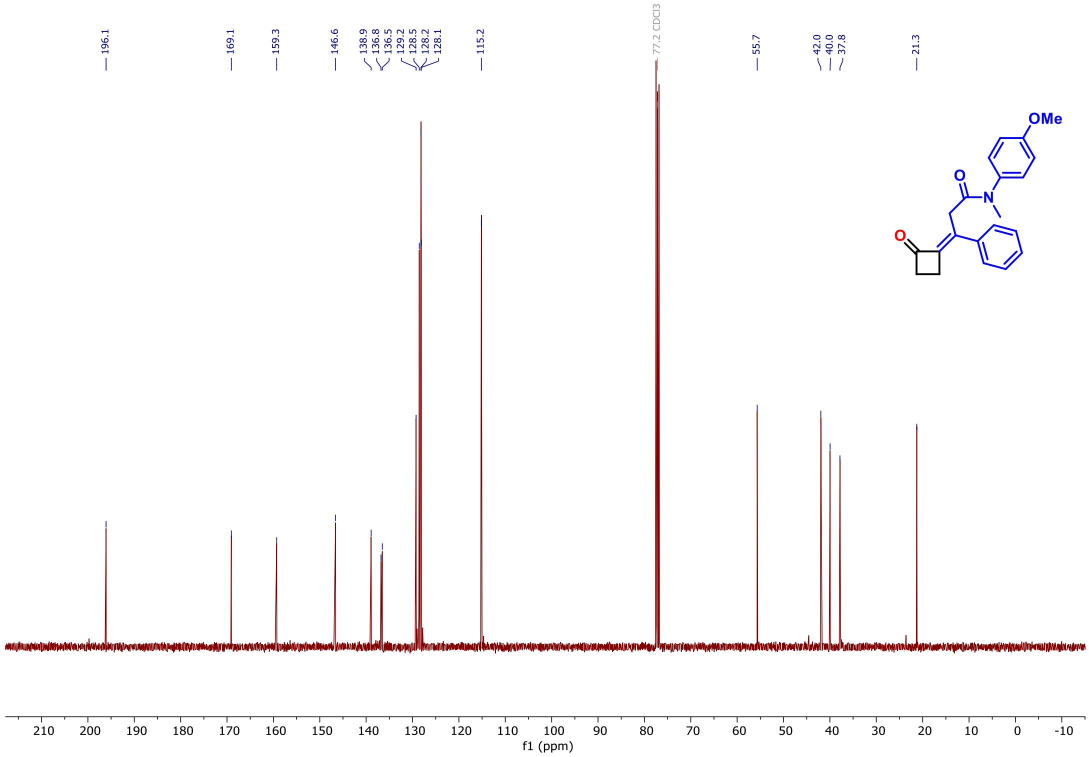

^{13}C{^{1}H}-NMR (101 MHz, $CDCl_3$) of **3am**

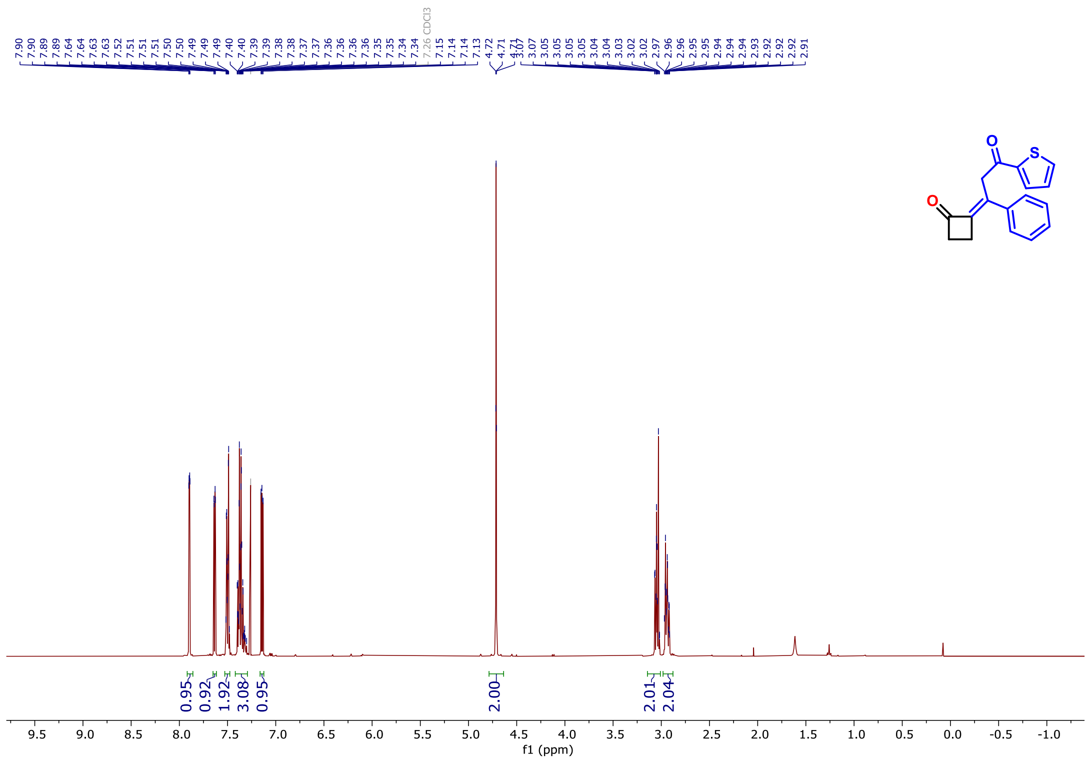

^{1}H-NMR (400 MHz, $CDCl_3$) of **3an**

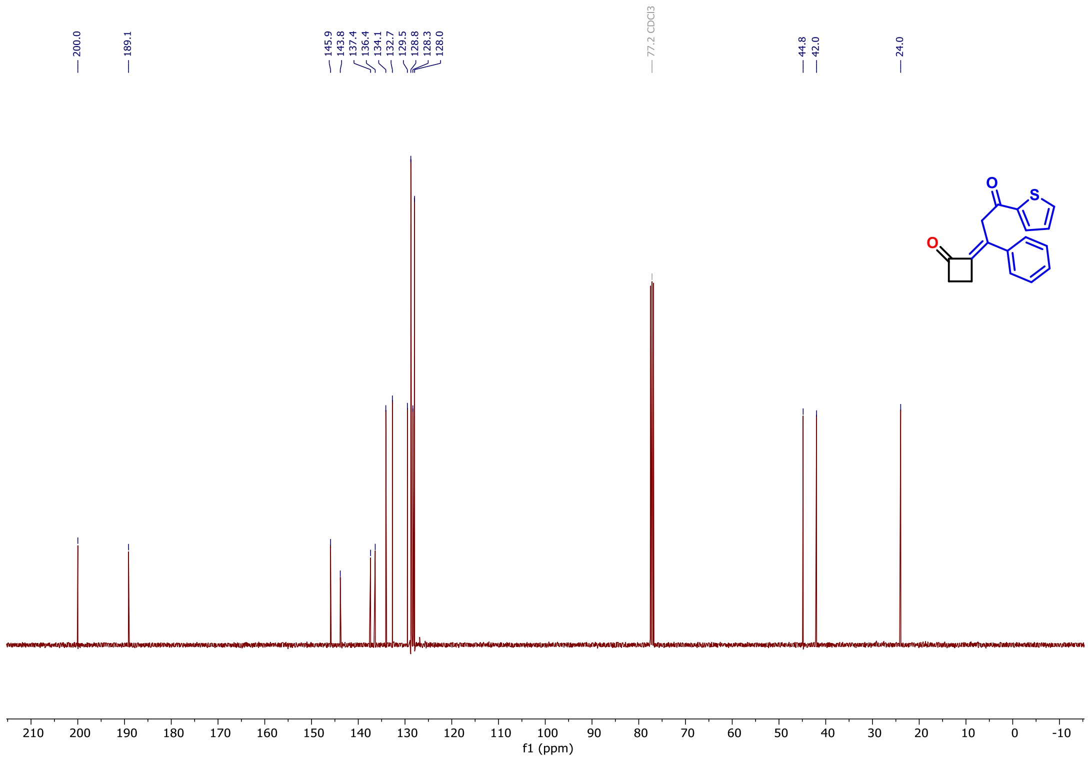

$^{13}C\{^1H\}$-NMR (101 MHz, $CDCl_3$) of **3an**

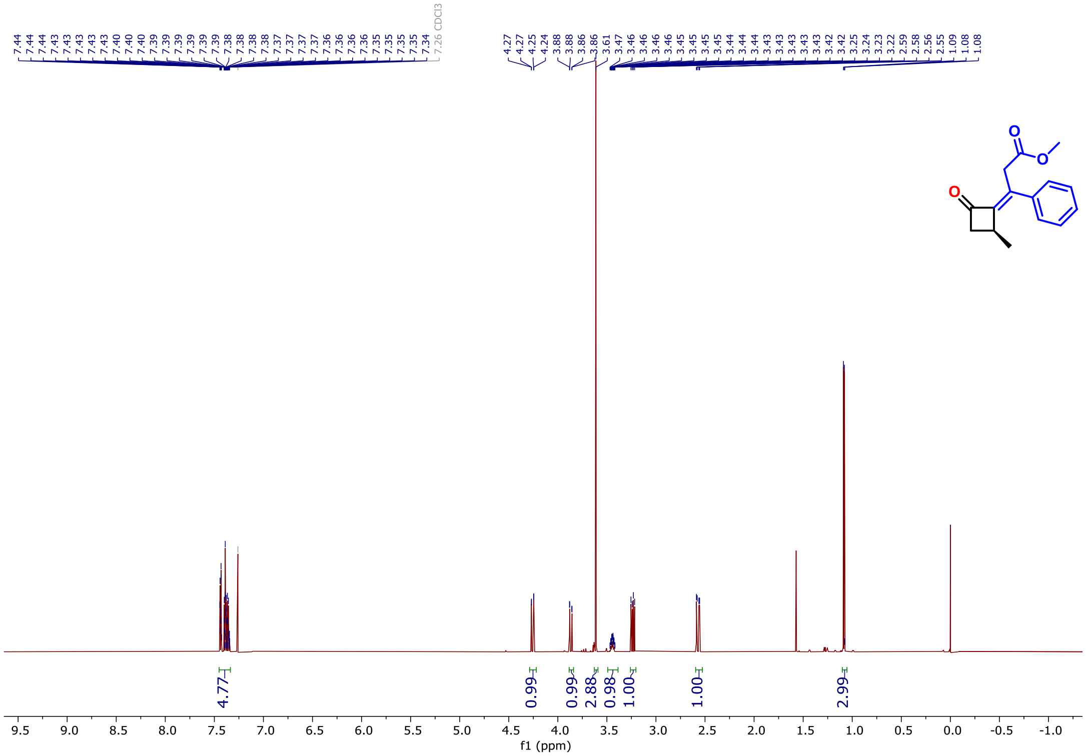

¹H-NMR (700 MHz, CDCl₃) of **3ba**

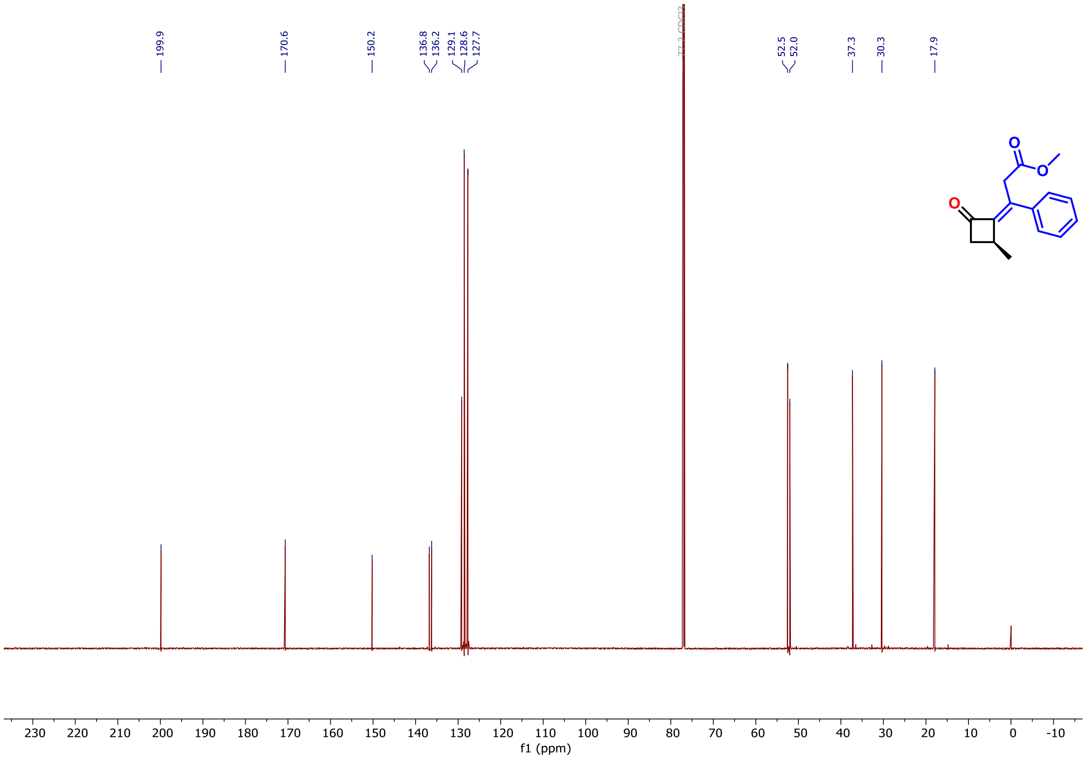

¹³C{¹H}-NMR (176 MHz, CDCl₃) of **3ba**

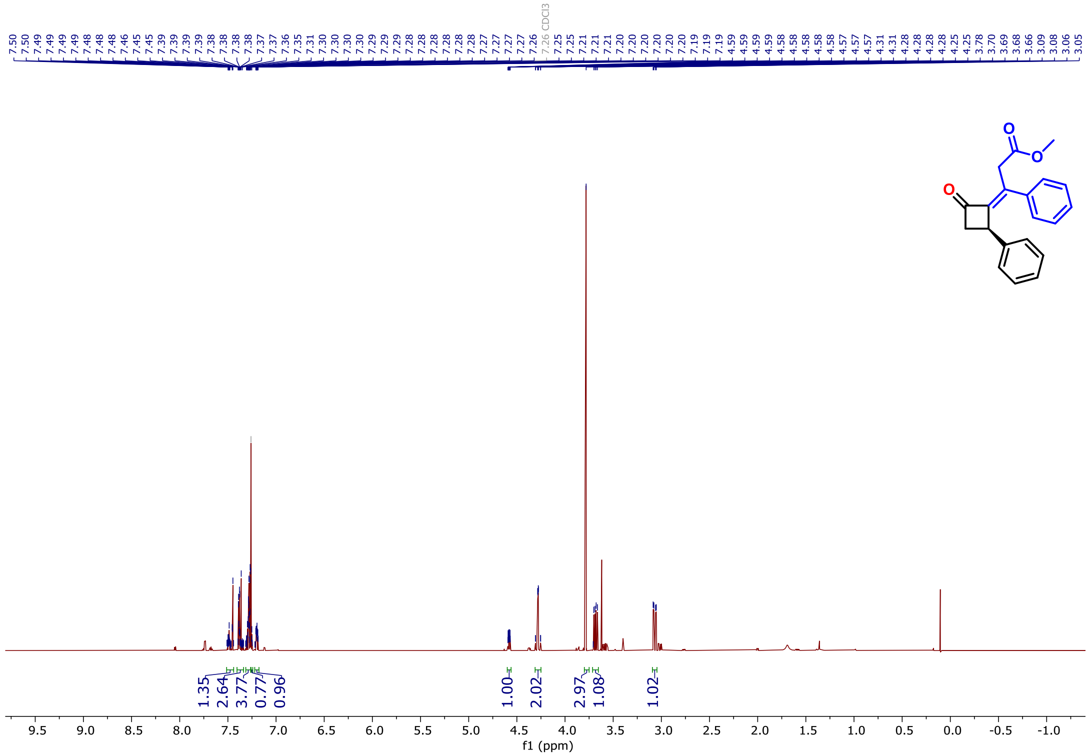

^{1}H-NMR (700 MHz, $CDCl_3$) of **3ca**

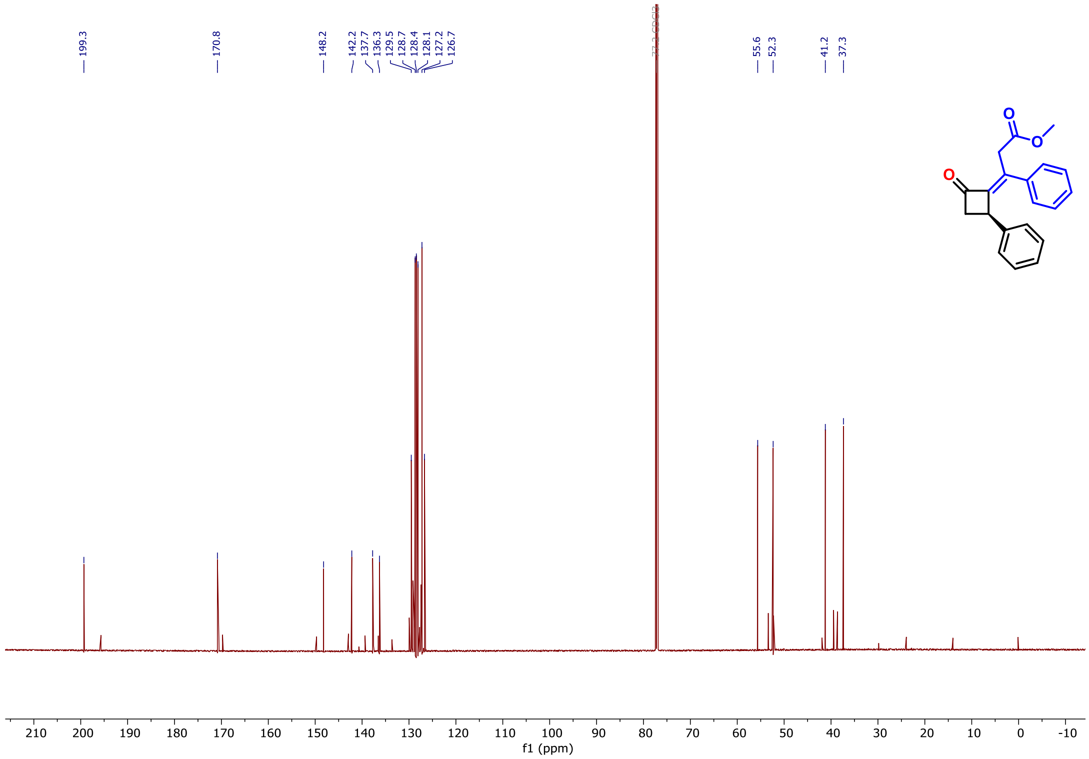

^{13}C{^{1}H}-NMR (176 MHz, $CDCl_3$) of **3ca**

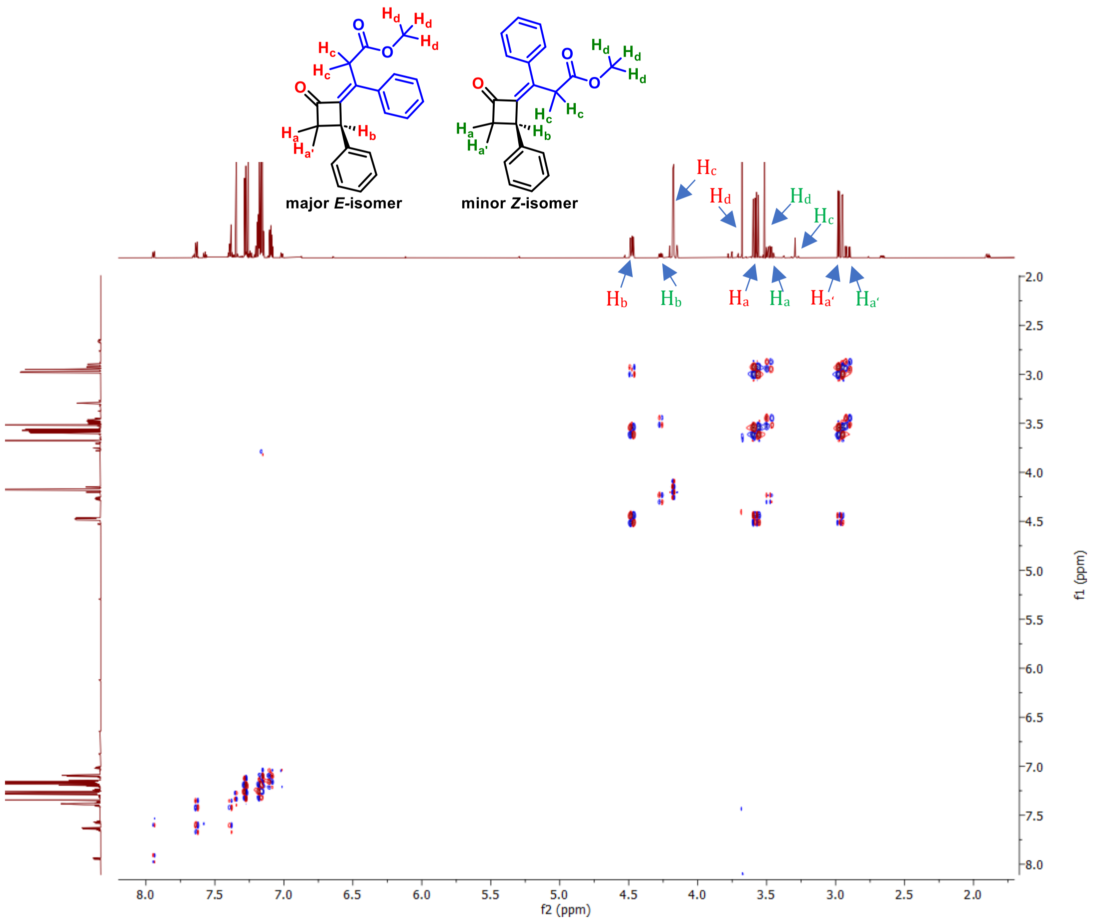

^{1}H-^{1}H-COSY NMR of **3ca**

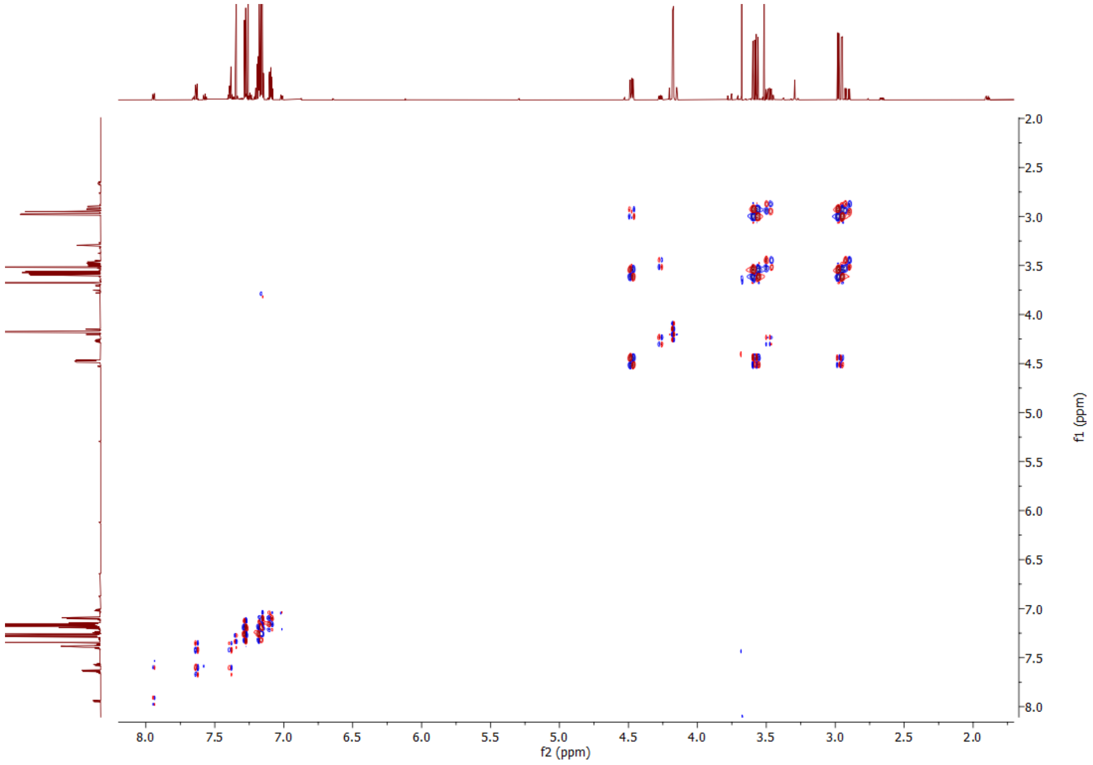

^{1}H-^{13}C-HSQC NMR of **3ca**

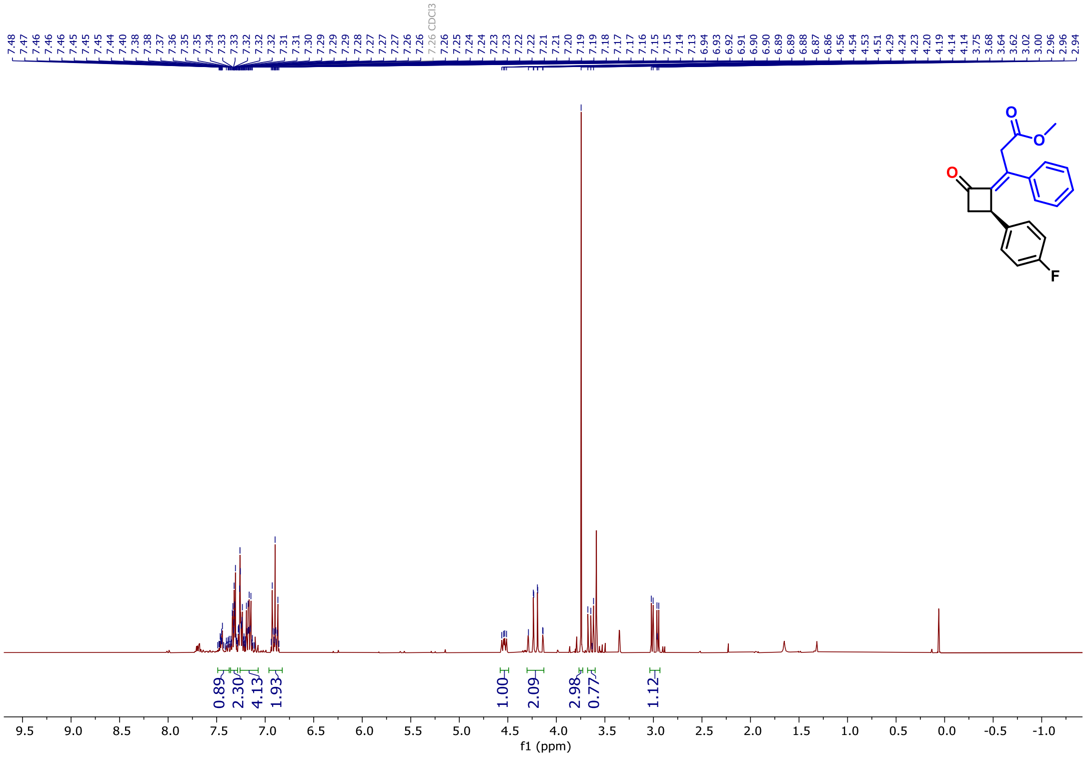

¹H-NMR (300 MHz, CDCl$_3$) of **3da**

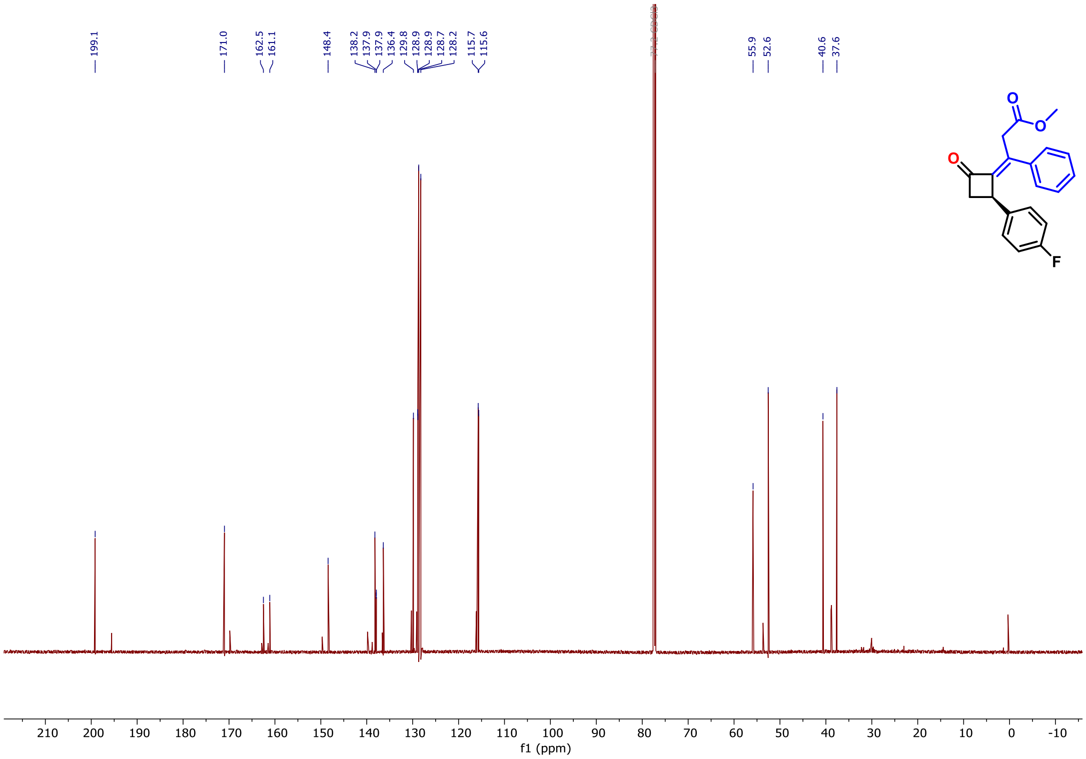

^{13}C{^{1}H}-NMR (176 MHz, CDCl$_3$) of **3da**

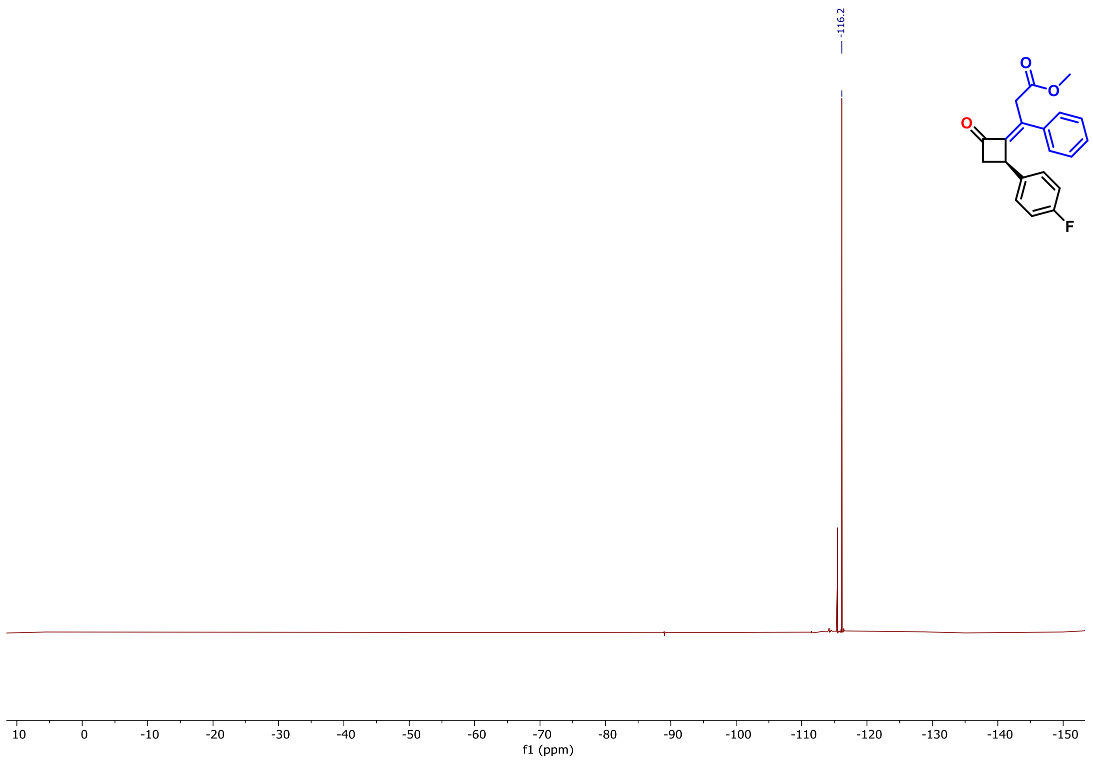

^{19}F NMR (659 MHz, $CDCl_3$) of **3da**

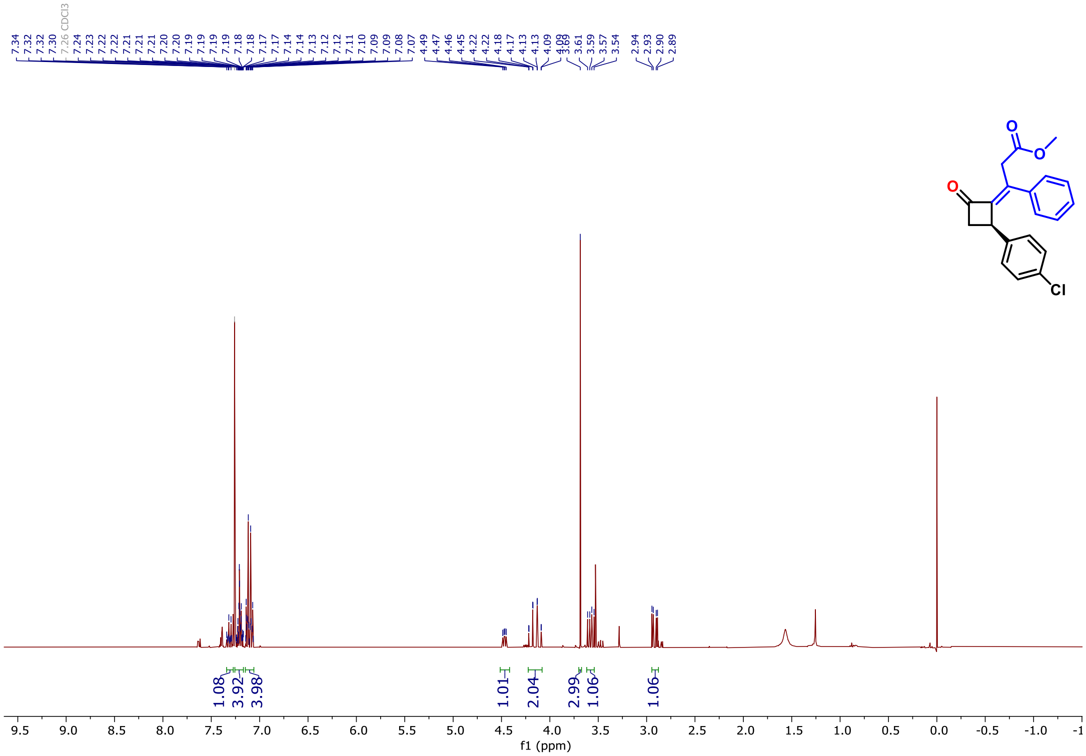

¹H-NMR (400 MHz, $CDCl_3$) of **3ea**

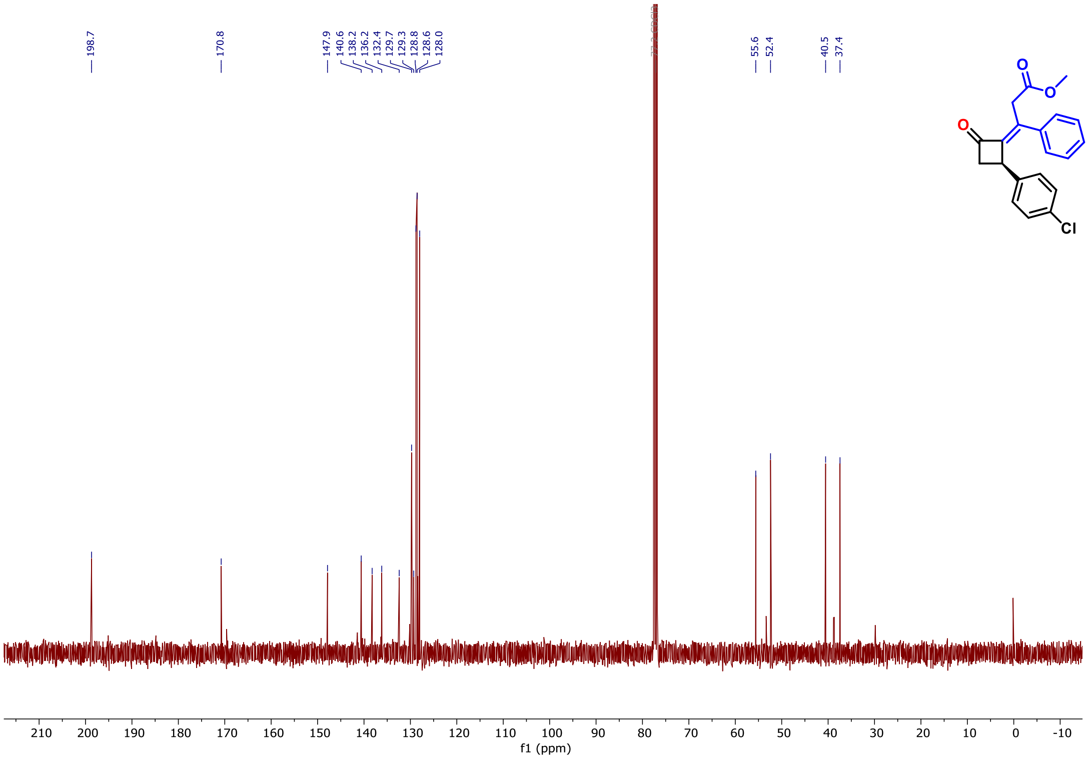

¹³C{¹H}-NMR (101 MHz, $CDCl_3$) of **3ea**

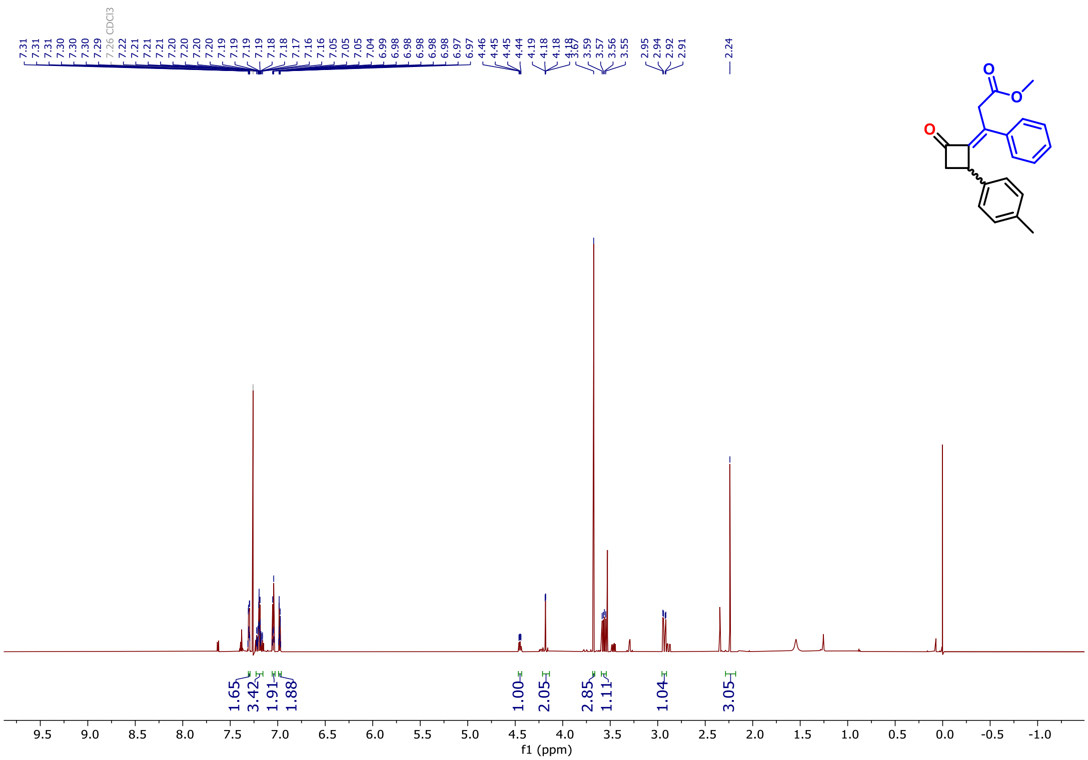

¹H-NMR (700 MHz, $CDCl_3$) of **3fa**

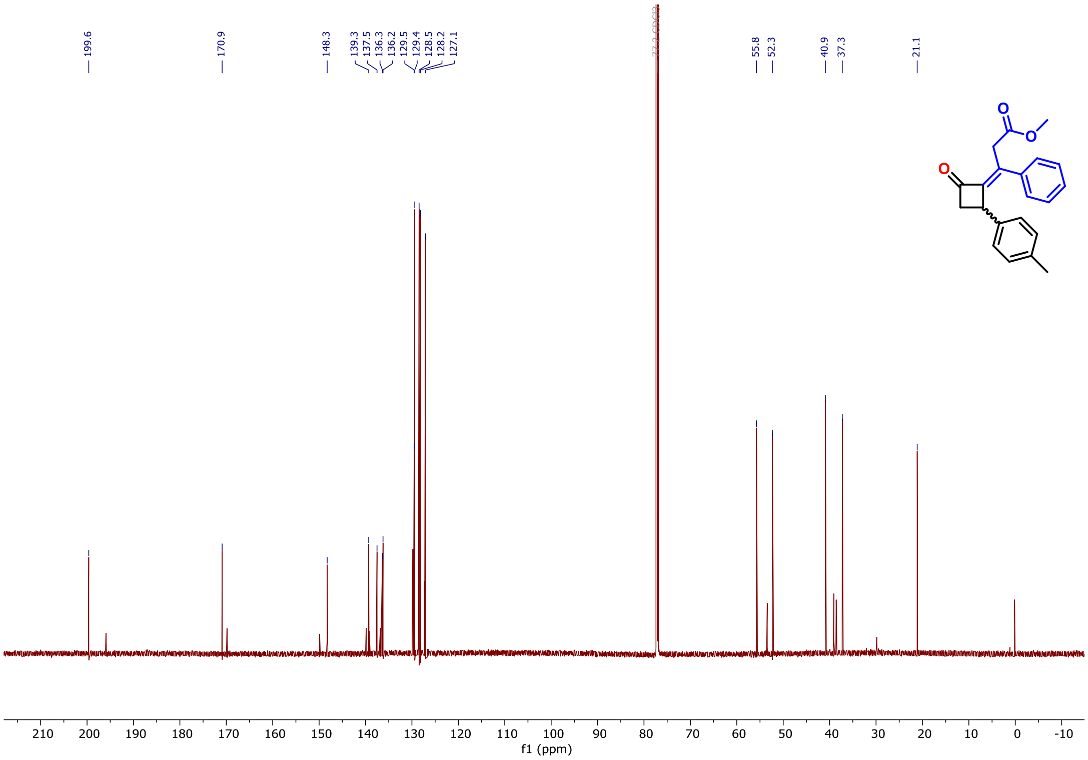

^{13}C{^{1}H}-NMR (176 MHz, $CDCl_3$) of **3fa**

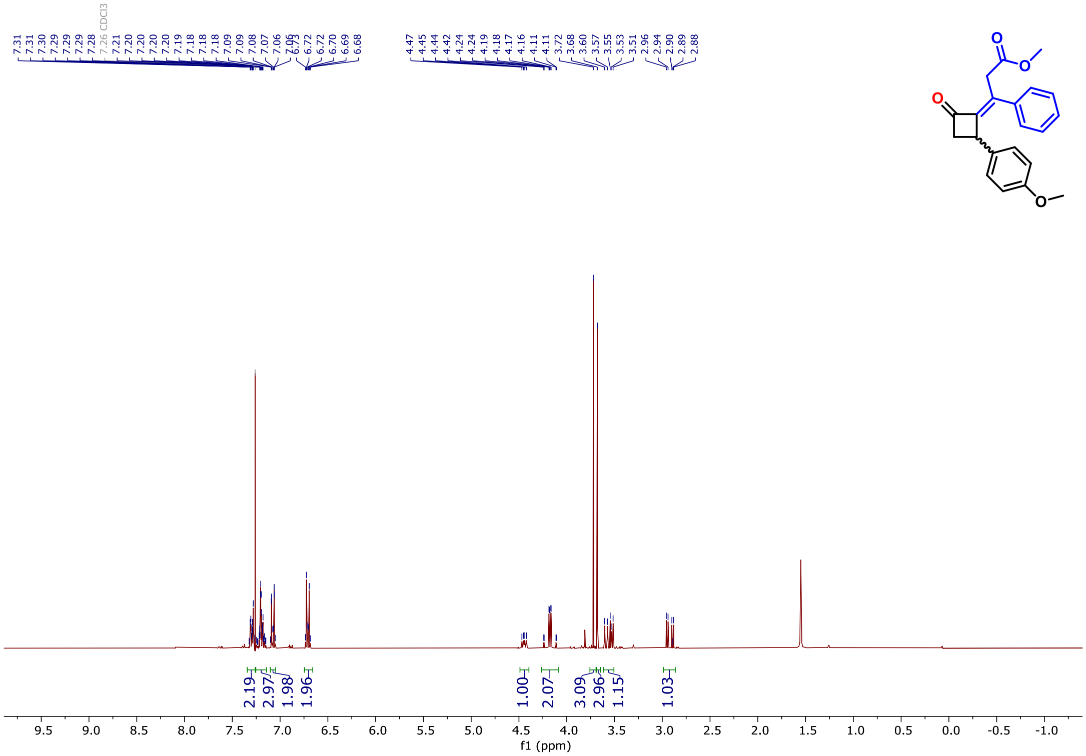

¹H-NMR (300 MHz, $CDCl_3$) of **3ga**

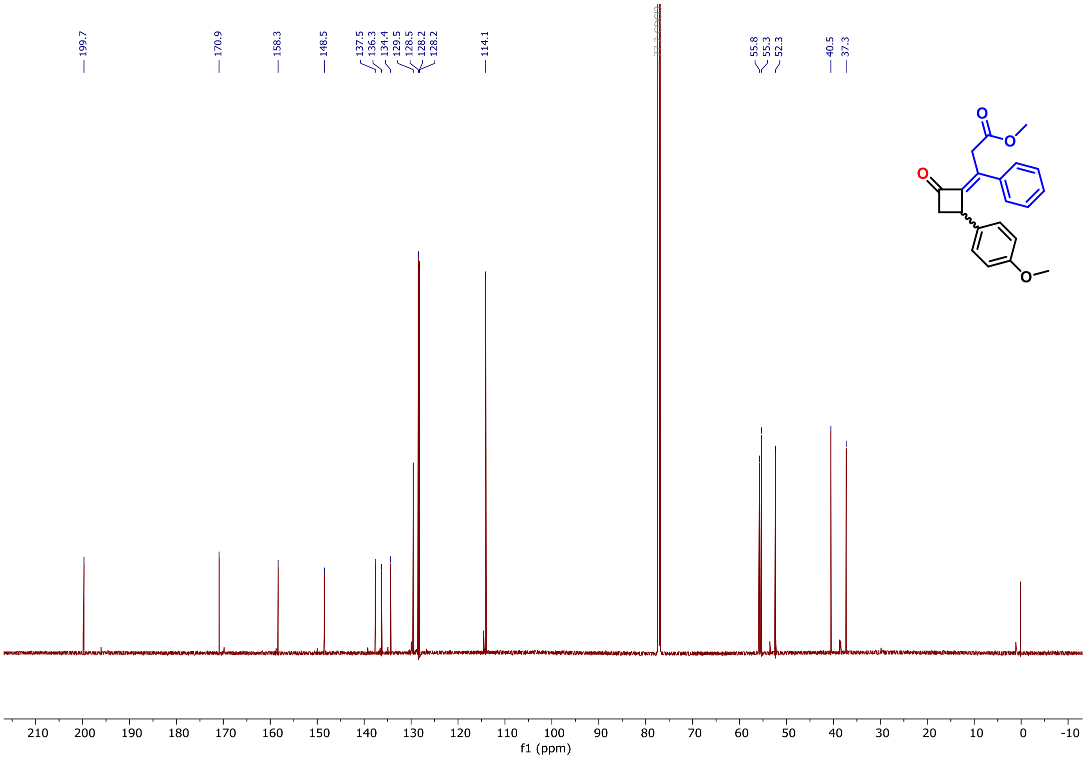

¹³C{¹H}-NMR (176 MHz, $CDCl_3$) of **3ga**

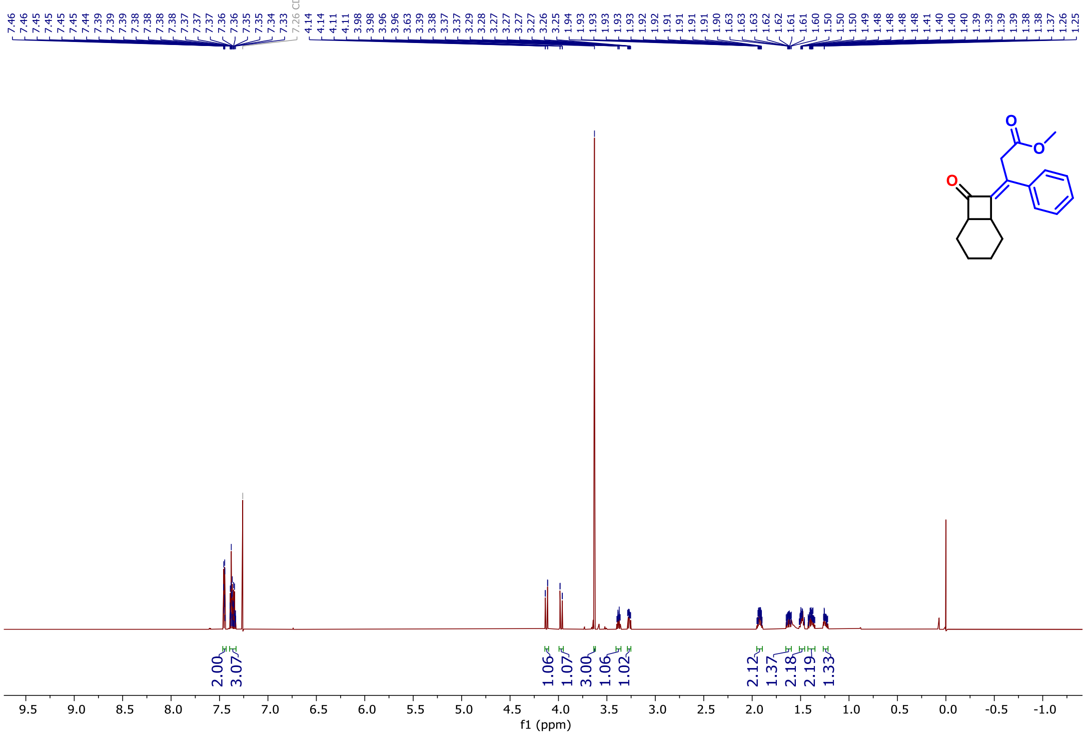

¹H-NMR (700 MHz, $CDCl_3$) of **3ha**

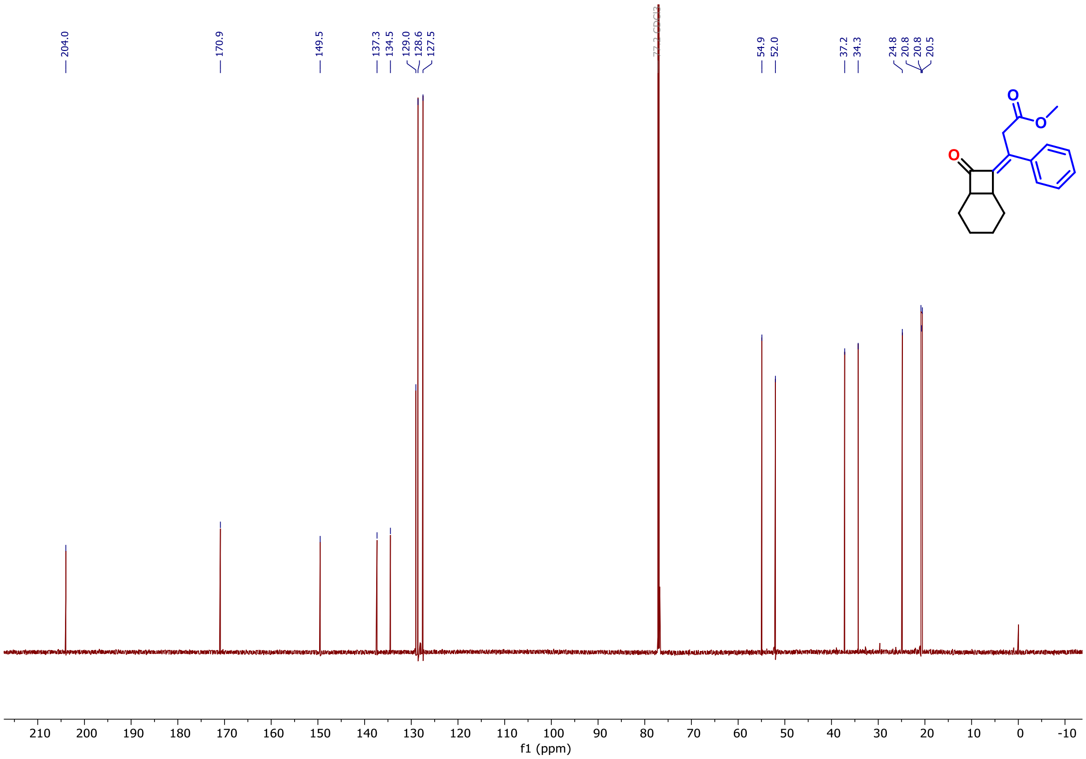

¹³C{¹H}-NMR (176 MHz, $CDCl_3$) of **3ha**

Follow-up Reactions: NMR spectra:

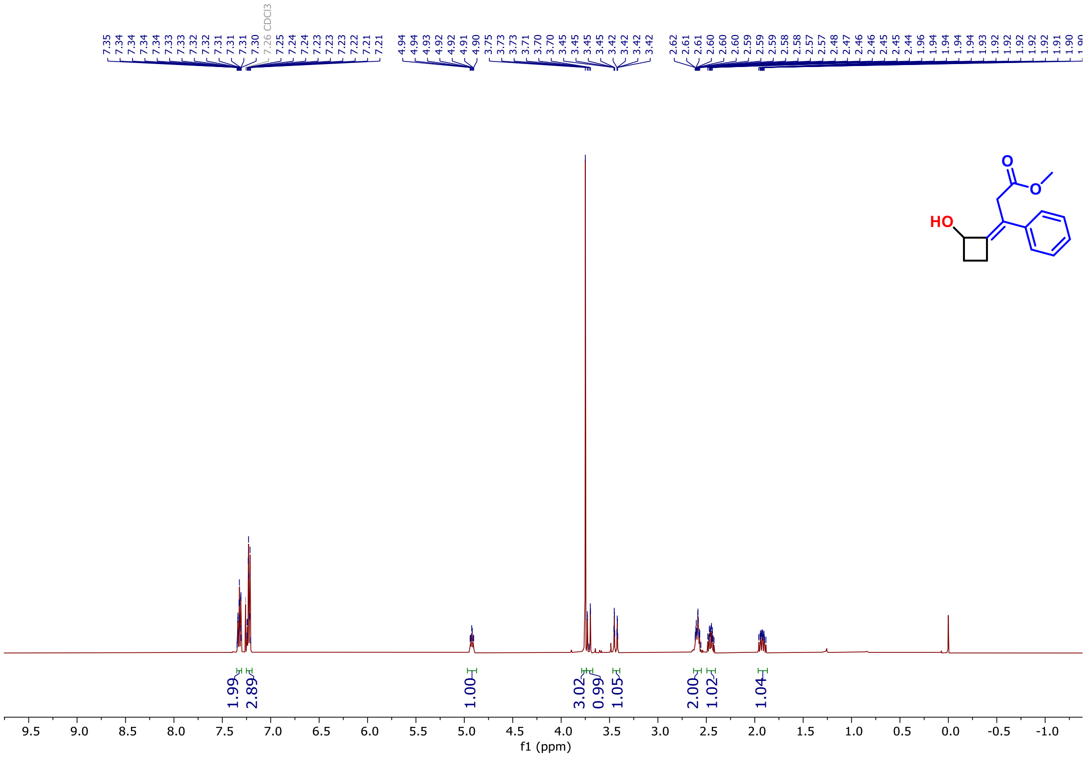

^{1}H-NMR (500 MHz, $CDCl_3$) of **4**

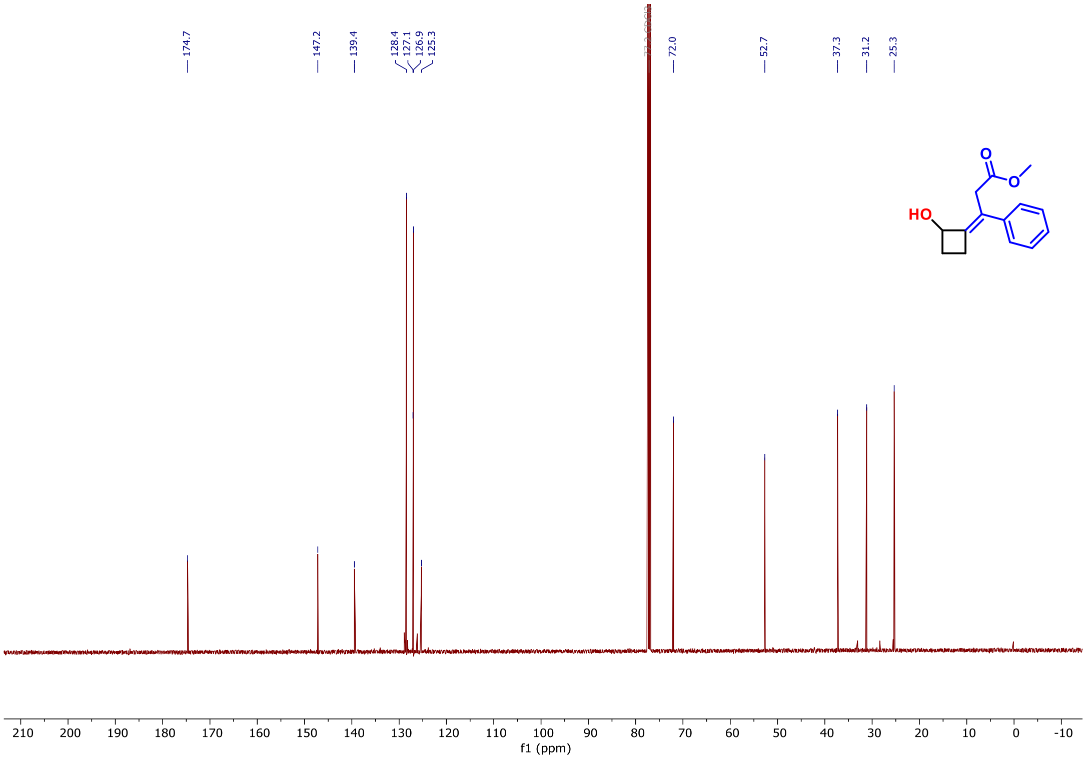

^{13}C{^{1}H}-NMR (126 MHz, $CDCl_3$) of **4**

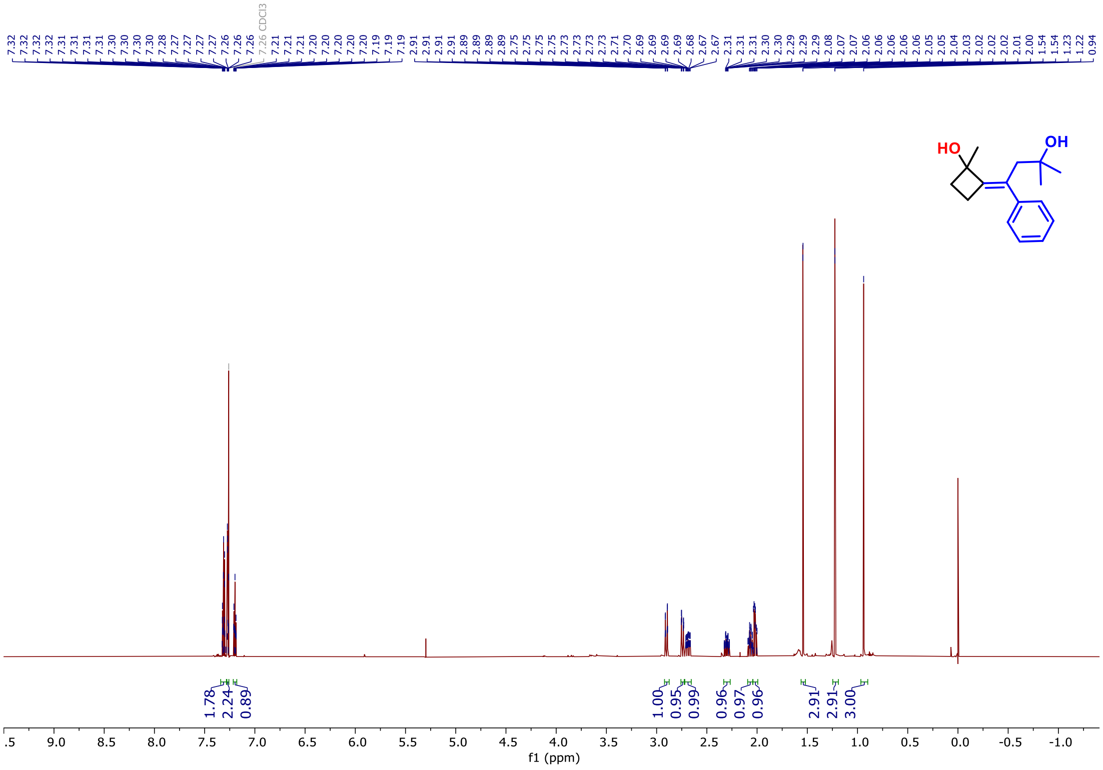

¹H-NMR (700 MHz, $CDCl_3$) of **5**

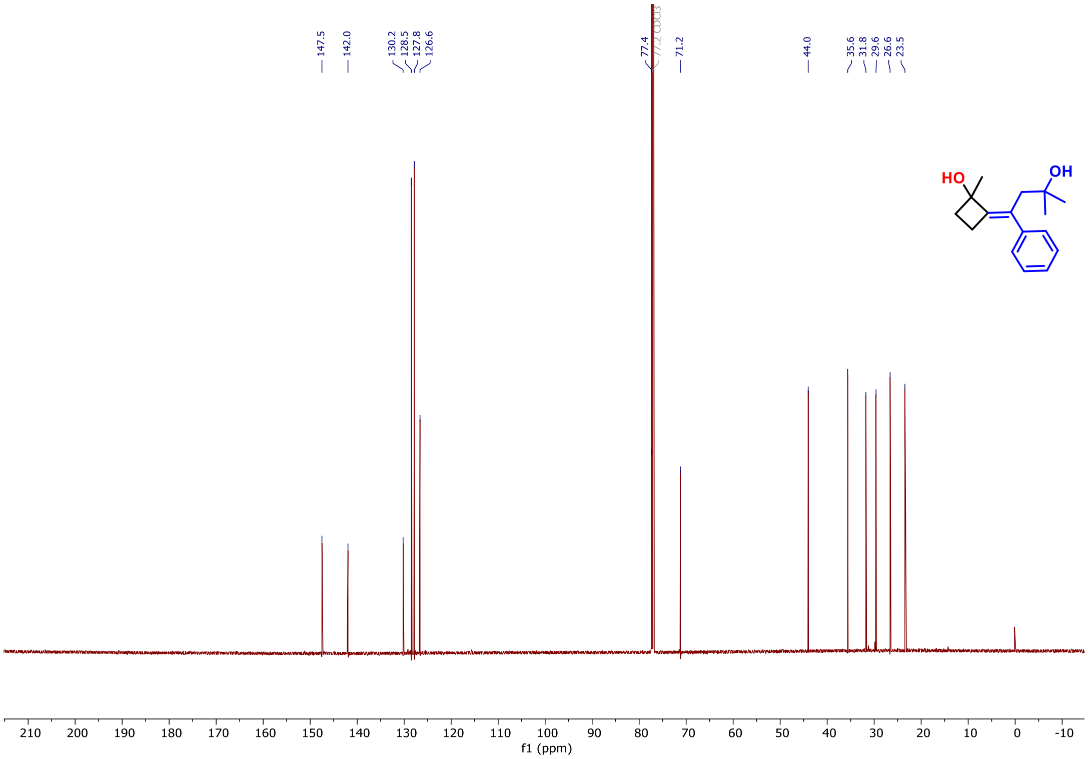

$^{13}C\{^1H\}$-NMR (176 MHz, $CDCl_3$) of **5**

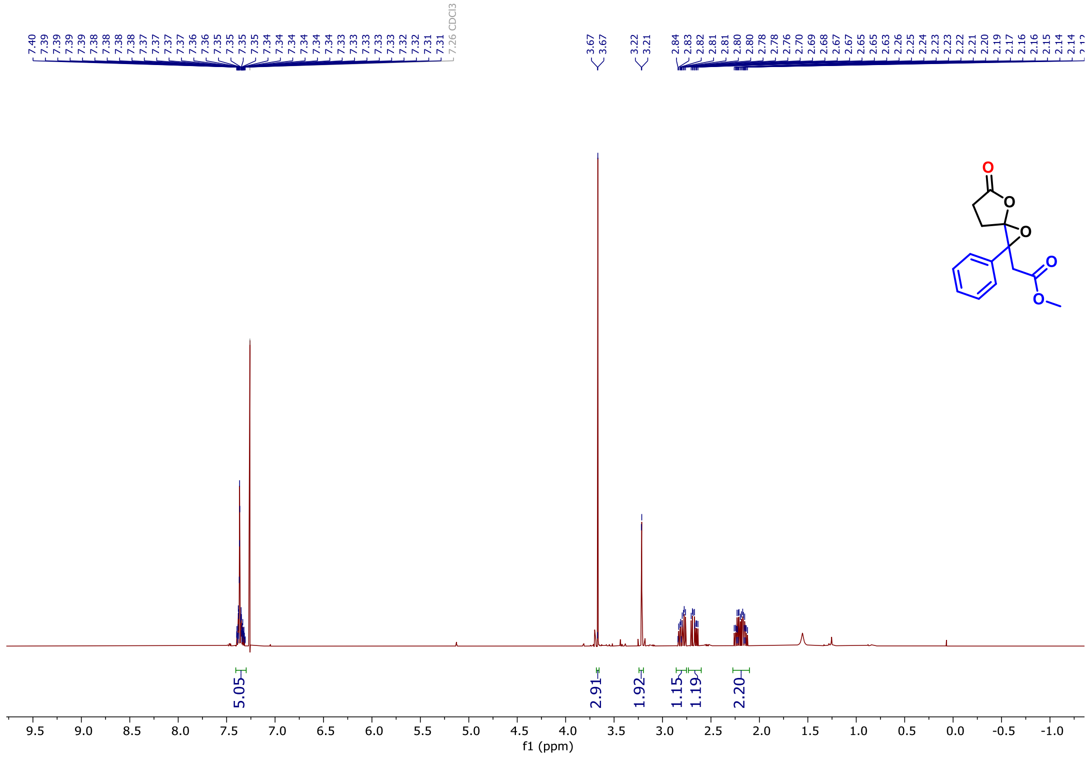

^{1}H-NMR (500 MHz, $CDCl_3$) of **6**

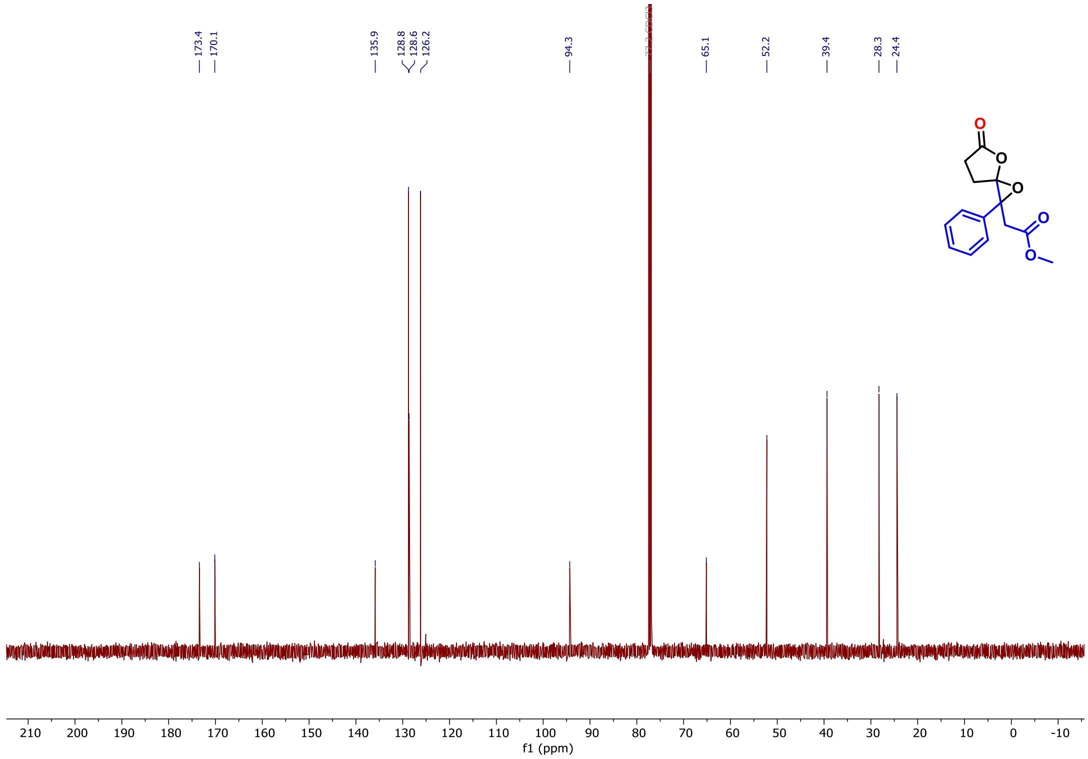

^{13}C{^{1}H}-NMR (126 MHz, $CDCl_3$) of **6**

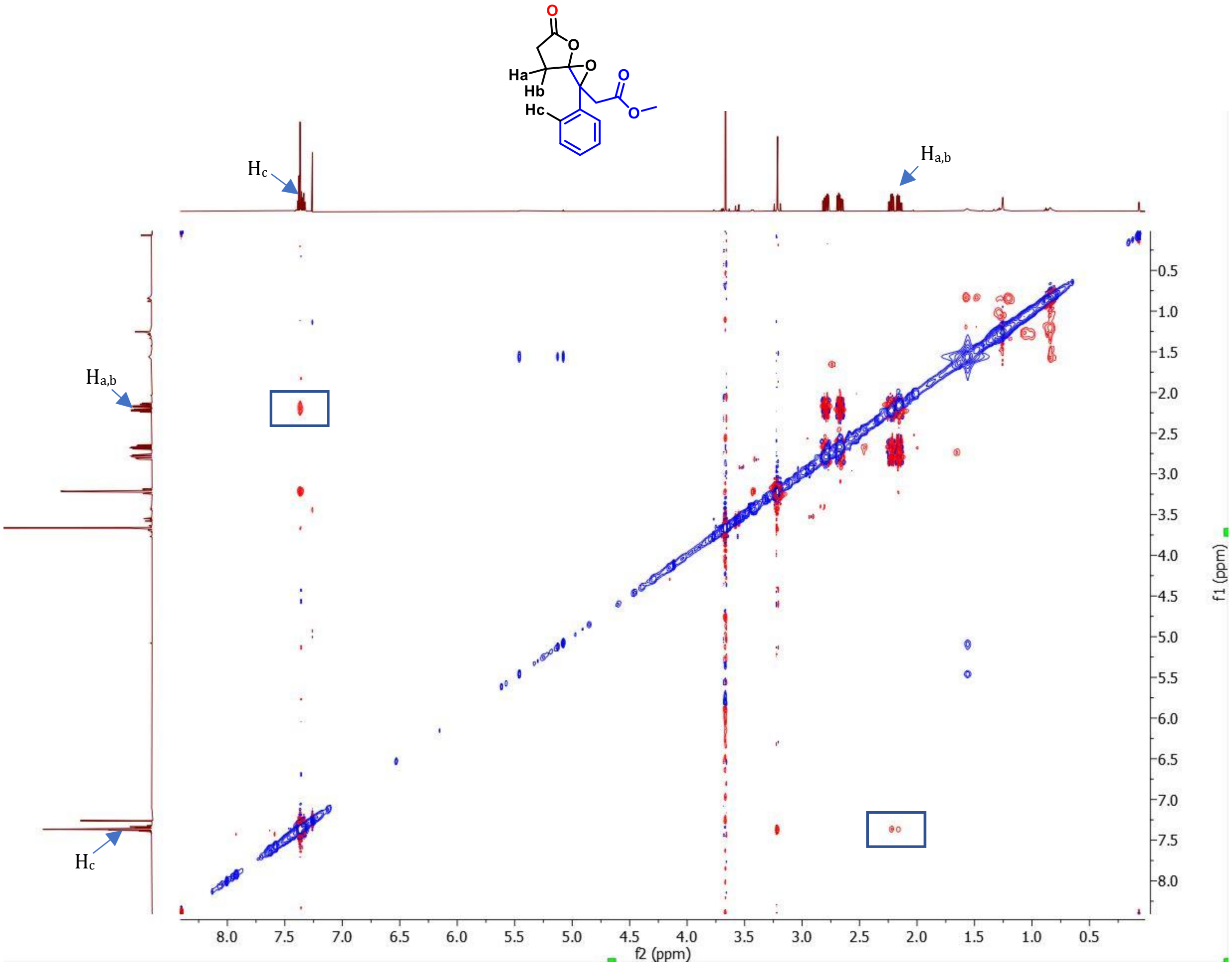

NOESY NMR of **6**

Stereoselectivity Experiments

Conditions for the separation of SCP 1b:

Enantiomeric excess was determined using a Chiralcel OD-3 column (3 µm; 150 x 4.6 mm) with *n*-heptane/*i*-PrOH (95:5) w/ 0.1% AcOH eluent mixtures. Detection was conducted at 218 nm using a 1290 Infinity II detector.

flow rate: 0.5 mL · min^{-1}

column temperature holds at 22 °C

Conditions for the separation of CB 3ba:

Enantiomeric excess was determined by HPLC analysis on chiral stationary phase. ChirakPAK AD-3 was used with *n*-heptane/*i*-PrOH eluent (98:2) mixtures. Detection was conducted at 288 nm using a variable wavelength detector (VWD).

flow rate: 0.5 mL · min^{-1}

column temperature holds at 22 °C

Conditions for the separation of SCP 1c:

Enantiomeric excess was determined using an Agilent 1260 Infinity column with a G1311B-1260 quaternary pump. A Chiralpak AD-3 column (3 µm; 150 x 4.6 mm) was used with *n*-heptane/EtOH (85/15) eluent mixtures. Detection was conducted at 230 nm using a 1290 Infinity II DAD detector.

flow rate: 0.5 mL · min^{-1}

column temperature holds at 22 °C.

Conditions for the separation of CB 3ca:

Enantiomeric excess was determined by HPLC analysis on chiral stationary phase. ChirakPAK AD-3 was used with *n*-heptane/EtOH eluent (80:20) mixtures. Detection was conducted at 288 nm using a variable wavelength detector (VWD).

flow rate: 0.5 mL · min^{-1}

column temperature holds at 22 °C.

Conditions for the separation of 3da:

Enantiomeric excess of **3da** was determined by HPLC analysis on chiral stationary phase. ChirakPAK AD-3 was used with *n*-heptane/EtOH eluent (80:20) mixtures. Detection was conducted at 288 nm using a variable wavelength detector (VWD), flow rate: 0.5 mL · min^{-1}, column temperature holds at 22 °C.

Conditions for the separation of 3ea:

Enantiomeric excess of **3ea** was determined by HPLC analysis on chiral stationary phase. ChirakPAK AD-3 was used with *n*-heptane/EtOH eluent (80:20) mixtures. Detection was conducted at 288 nm using a variable wavelength detector (VWD), flow rate: 0.5 mL · min^{-1}, column temperature holds at 22 °C.

Conditions for the separation of 3fa:

For HPLC analysis of **3fa**, chiral stationary phase ChirakPAK AD-3 was used with *n*-heptane/EtOH eluent (80:20) mixtures. Detection was conducted at 288 nm using a variable wavelength detector (VWD), flow rate: 0.5 mL · min^{-1}, column temperature holds at 22 °C.

Conditions for the separation of 3ga:

For HPLC analysis of **3ga**, chiral stationary phase ChirakPAK AD-3 was used with *n*-heptane/EtOH eluent (80:20) mixtures. Detection was conducted at 288 nm using a variable wavelength detector (VWD), flow rate: 0.5 mL · min^{-1}, column temperature holds at 22 °C.

Chromatogram of racemic SCP **1b**.

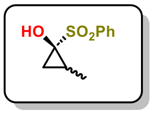

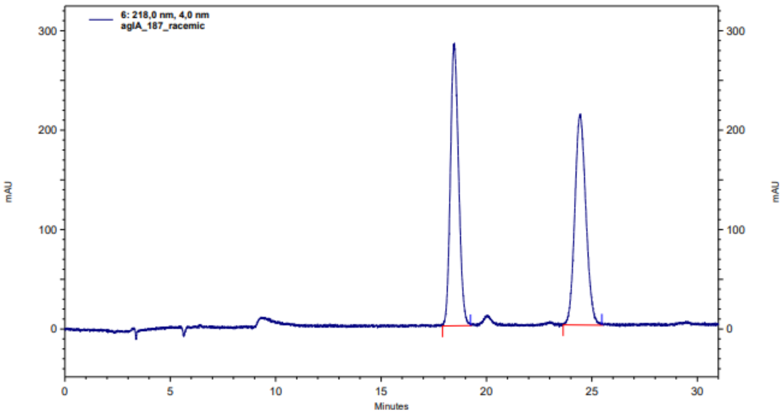

6: 218,0 nm, 4,0 nm
Results

Peak Number	Retention Time	Area Percent	Area
1	18,482	49,875	1033535424
2	24,455	50,125	1038701563
Totals		100,000	2072236987

Chromatogram of enantioenriched SCP **1b**.

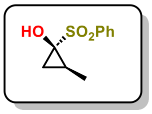

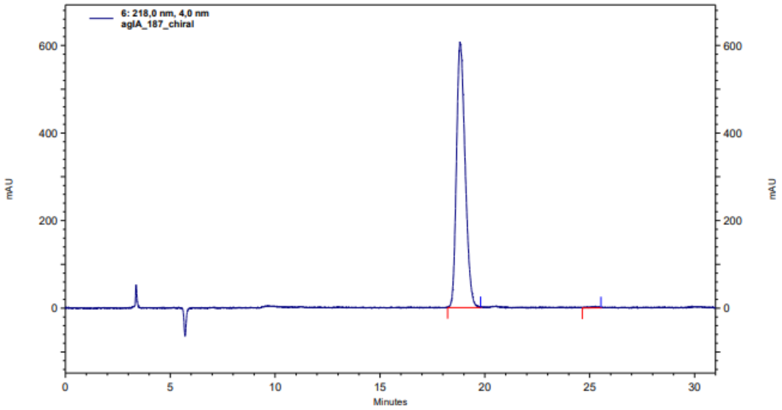

6: 218,0 nm, 4,0 nm
Results

Peak Number	Retention Time	Area Percent	Area
1	18,815	99,473	2336721448
2	25,245	0,527	12373090
Totals		100,000	2349094538

Chromatogram of racemic CB **3ba**.

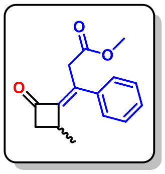

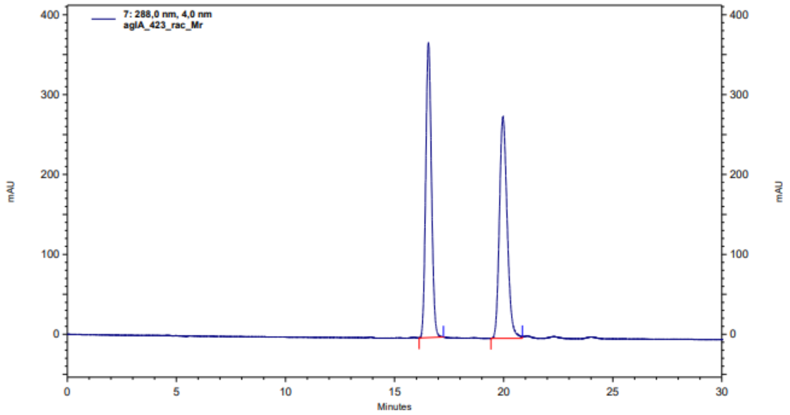

7: 288,0 nm, 4,0 nm
Results

Peak Number	Retention Time	Area Percent	Area
1	16,553	49,580	905307869
2	19,967	50,420	920630811
Totals		100,000	1825938680

Chromatogram of chiral CB **3ba**.

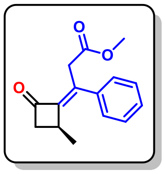

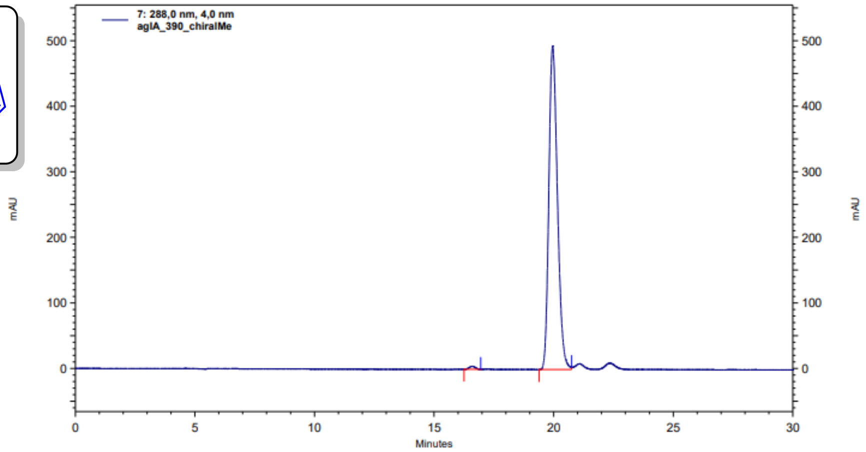

7: 288,0 nm, 4,0 nm
Results

Peak Number	Retention Time	Area Percent	Area
1	16,575	0,654	10889811
2	19,957	99,346	1655061504
Totals		100,000	1665951315

Chromatogram of racemic SCP **1c**

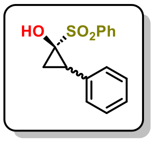

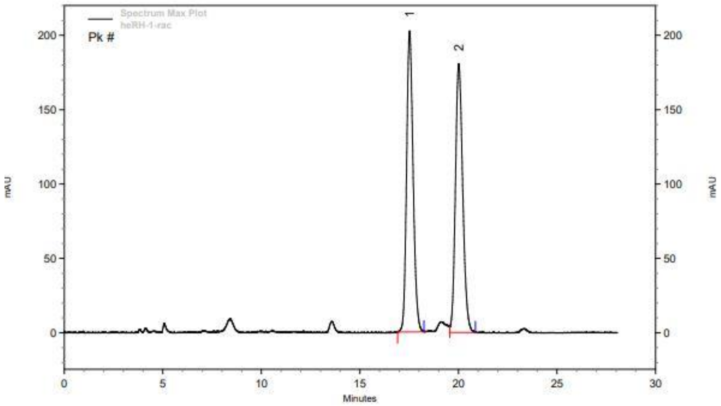

Spectrum Max Plot Results Pk #	Retention Time	Area Percent	Lambda Max
1	17,520	49,964	204
2	20,020	50,036	204

Chromatogram of chiral Ph subs SCP **1c**

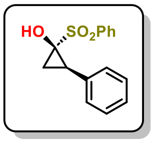

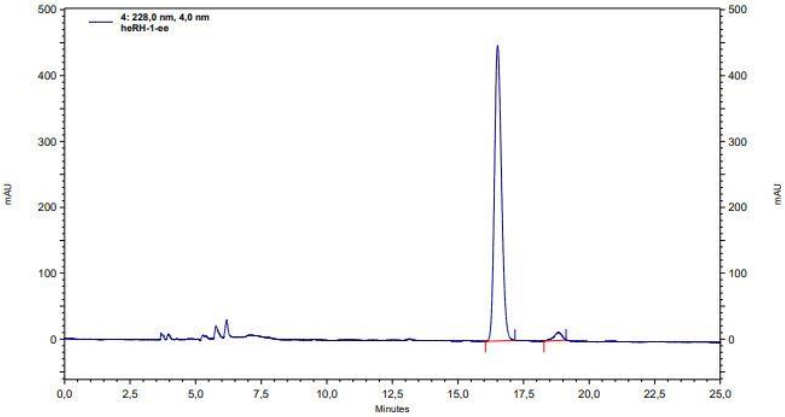

4: 228,0 nm, 4,0 nm

Results Peak Number	Retention Time	Area Percent	Area
1	16,512	97,043	1128893214
2	18,818	2,957	34398691
Totals		100,000	1163291905

Chromatogram of racemic Ph subs CB **3ca**.

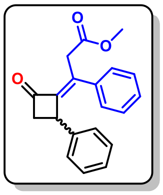

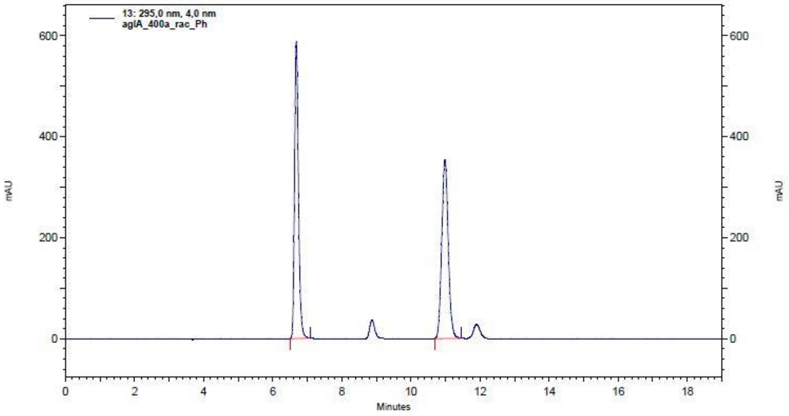

13: 295,0 nm, 4,0 nm
Results

Peak Number	Retention Time	Area Percent	Area
1	6,683	49,949	619404889
2	10,987	50,051	620669625
Totals		100,000	1240074514

Chromatogram of chiral Ph subs CB **3ca**.

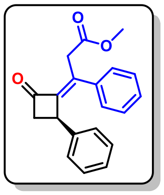

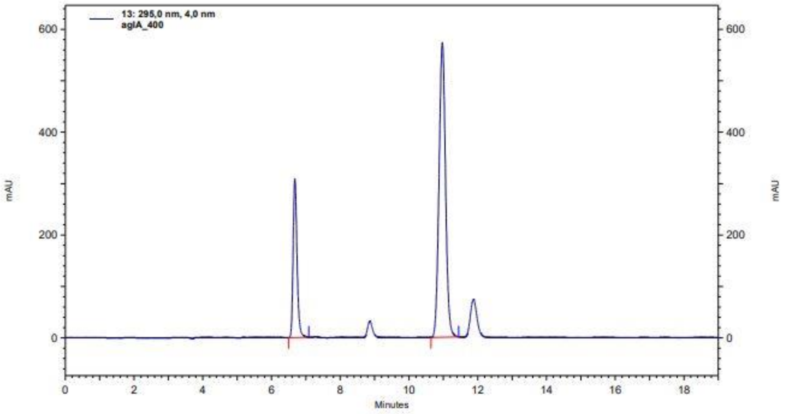

13: 295,0 nm, 4,0 nm
Results

Peak Number	Retention Time	Area Percent	Area
1	6,680	24,478	327120108
2	10,972	75,521	1009236844
Totals		100,000	1336356952

Chromatogram of **3da**

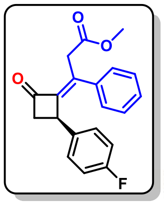

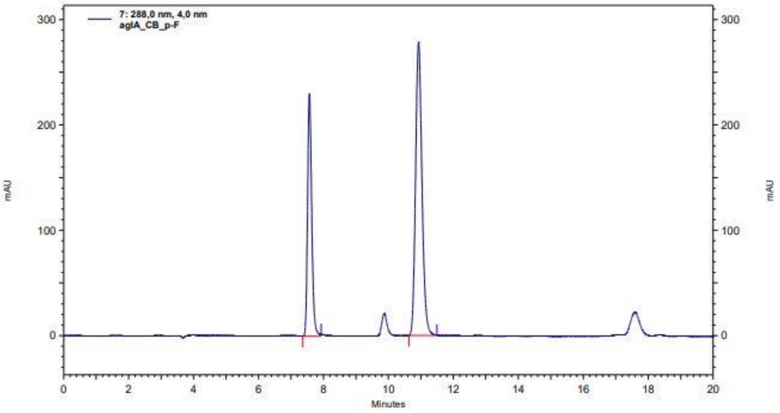

7: 288,0 nm, 4,0 nm
Results

Peak Number	Retention Time	Area Percent	Area
1	7,568	34,751	272071851
2	10,928	65,249	510848413
Totals		100,000	782920264

Chromatogram of **3ea**

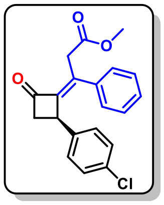

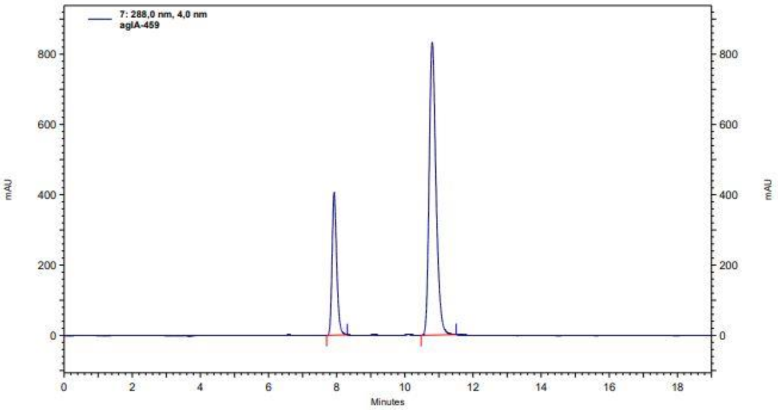

7: 288,0 nm, 4,0 nm
Results

Peak Number	Retention Time	Area Percent	Area
1	7,925	24,779	513835672
2	10,803	75,221	1559843291
Totals		100,000	2073678963

Chromatogram of **3fa**

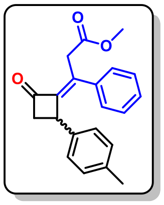

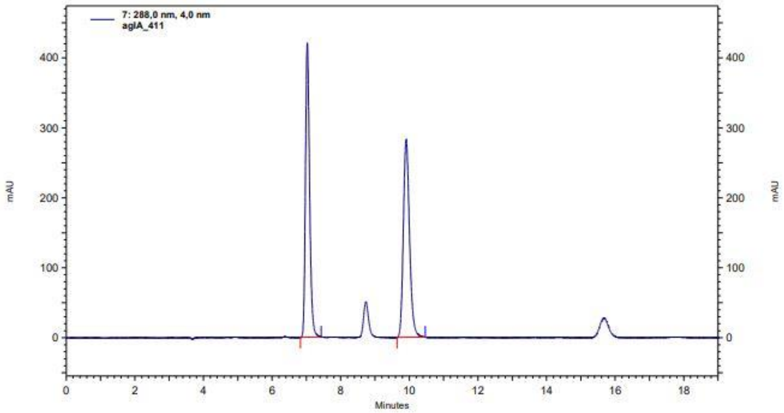

7: 288,0 nm, 4,0 nm

Results

Peak Number	Retention Time	Area Percent	Area
1	7,027	49,917	474566560
2	9,908	50,083	476135566
Totals		100,000	950702126

Chromatogram of **3ga**

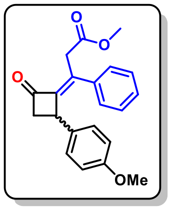

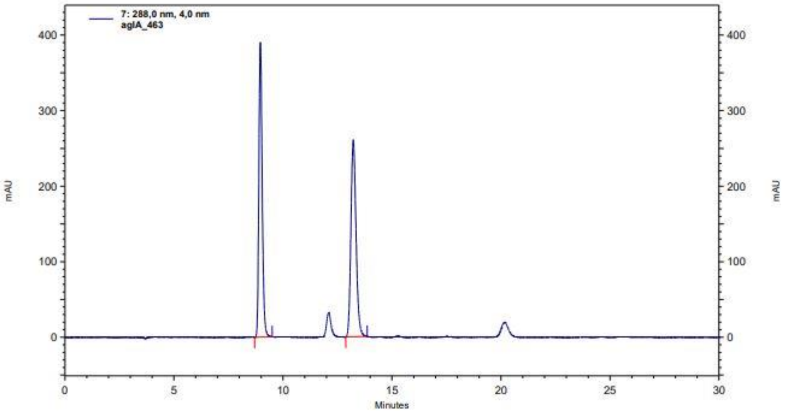

7: 288,0 nm, 4,0 nm

Results

Peak Number	Retention Time	Area Percent	Area
1	8,965	49,298	554391786
2	13,227	50,702	570179661
Totals		100,000	1124571447

Optimization of Reaction Conditions

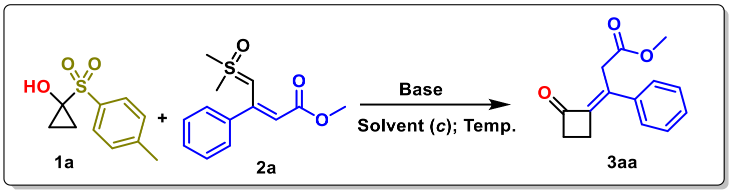

Initial Screening[a]

Table S1

Entry	SCP[b]	Sulfoxonium Ylide	Base	Solvent	Temp. (°C)	NMR yield[c] (%)
1	**1a**	**2a**	LiHMDS	CH_2Cl_2	−78 to rt	traces
2	**1a**	**2a**	KHMDS	CH_2Cl_2	−78 to rt	traces
3	**1a**	**2a**	NEt_3	CH_2Cl_2	−78 to rt	62
4	**1a**	**2a**	NEt_3	CH_2Cl_2	0 to rt	64
5	**1a**	**2a**	KOH	CH_2Cl_2	0 to rt	37
6	**1a**	**2a**	Cs_2CO_3	CH_2Cl_2	0 to rt	44
7	**1a**	**2a**	DIPEA	CH_2Cl_2	0 to rt	80
8	**1a**	**2a**	DIPEA	DCE	0 to rt	77
9	**1a**	**2a**	DIPEA	$CHCl_3$	0 to rt	79
10	**1a**	**2a**	DIPEA	THF	0 to rt	46
11	**1a**	**2a**	DIPEA	DMF	0 to rt	55
12	**1a**	**2a**	DIPEA	DMSO	0 to rt	62
13	**1a**	**2a**	DIPEA	EtOAc	0 to rt	66
14	**1a**	**2a**	DIPEA	Toluene	0 to rt	61

[a]General procedure A: A stock solution of **1a** (1.0 eq.) and **2a** (1.5 eq.) in anhydrous solvent (0.05 M) was prepared and distributed to multiple reaction vials. After cooling to above mentioned temperature, the respective base (1.5 eq.) was added dropwise. After warming to rt overnight, the crude reaction mixture was analyzed by GC/MS. [b]All reactions were carried out on a 0.1 mmol scale with respect to SCP **1a**. [c]Yields refer to ^{1}H-NMR yield against a 1,3,5-trimethoxybenzene standard.

Miscellaneous Screening[a]

Table S2

Entry	SCP[b]	Sulfoxonium Ylide (eq.)	Base (eq.)	Solvent (c/M)	Temp. (°C)	NMR yield (%)[c]
**1**	**1a**	**2a** (1.5)	DIPEA (1.5)	CH_2Cl_2 (0.05)	0 to rt	80
**2**	**1a**	**2a** (2.0)	DIPEA (1.5)	CH_2Cl_2 (0.05)	0 to rt	74
**3**	**1a**	**2a** (1.0)	DIPEA (1.5)	CH_2Cl_2 (0.05)	0 to rt	69
**4**	**1a**	**2a** (1.5)	DIPEA (3.4)	CH_2Cl_2 (0.03)	0 to rt	80
**5**	**1a**	**2a** (1.5)	DIPEA (3.4)	CH_2Cl_2 (0.05)	0 to rt	95
**6**	**1a**	**2a** (1.5)	DIPEA (3.4)	CH_2Cl_2 (0.1)	0 to rt	76
**7**	**1a**	**2a** (1.5)	DIPEA (3.4)	CH_2Cl_2 (0.05)	rt	83

[a] General procedure A: A stock solution of **1a** (1.0 eq.) and **2a** in anhydrous solvent was prepared and distributed to multiple reaction vials. After cooling to above mentioned temperature, the respective base was added dropwise. After warming to rt overnight, the crude reaction mixture was analyzed by GC/MS. [b]All reactions were carried out on a 0.1 mmol scale with respect to SCP **1a**. [c]Yields refer to ^{1}H-NMR yield against a 1,3,5-trimethoxybenzene standard.

Single-Crystal X-Ray Diffraction

Data were collected from shock-cooled single crystals at 100(2) K on a Bruker APEX2 QUAZAR three-circle diffractometer with a microfocus sealed X-ray tube using a mirror optics as monochromator and a Bruker APEXII detector and a Bruker D8 VENTURE dual wavelength Mo/Cu three-circle diffractometer with a microfocus sealed X-ray tube using a mirror optics as monochromator and a Bruker PHOTON III detector. The crystals were mounted on a MiTeGen micromount with perfluoroether oil. The diffractometers are equipped with an Oxford Cryostream 800 low temperature device. Both MoK_{α} radiation (λ = 0.71073 Å) and CuK_{α} radiation (λ = 1.54178 Å) were used for the experiments. All data were integrated with SAINT V8.41 and a multi-scan absorption correction using SADABS 2016/2 was applied.[3,4] The structures were solved by direct methods with SHELXT and refined by full-matrix least-squares methods against F^2 using SHELXL-2019/2.[5,6] All non-hydrogen atoms were refined with anisotropic displacement parameters. All hydrogen atoms were refined isotropic on calculated positions using a riding model with their U_{iso} values constrained to 1.5 times the U_{eq} of their pivot atoms for terminal sp^3 carbon atoms and 1.2 times for all other carbon atoms. Crystallographic data for the structures (**Table S3**) have been deposited with the Cambridge Crystallographic Data Centre.[7] CCDC 2465160 (**3aa**), CCDC 2477661 (**3aj**), CCDC 2477635 (**3ak**), and CCDC 2491176 (**3ba**) contain the supplementary crystallographic data for this paper. These data can be obtained free of charge from The Cambridge Crystallographic Data Centre via www.ccdc.cam.ac.uk/structures. This report and the CIF file were generated using FinalCif.[8]

Table S3. Crystallographic data and structure refinement details for compound 3aa, 3aj, 3ak, and 3ba.

CCDC number	2465160 (**3aa**)	2477661 (**3aj**)	2477635 (**3ak**)	2491176 (**3ba**)
Empirical formula	$C_{14}H_{14}O_3$	$C_{19}H_{16}O_2$	$C_{20}H_{18}O_3$	$C_{15}H_{16}O_3$
Formula weight	230.25	276.32	306.34	244.28
Crystal size [mm³]	0.160×0.220×0.357	0.086×0.156×0.160	0.080×0.138×0.602	0.377×0.397×0.412
Crystal shape	block	block	block	block
Crystal system	triclinic	orthorhombic	triclinic	orthorhombic
Space group (number)	$P\overline{1}$ (2)	$Pbca$ (61)	$P\overline{1}$ (2)	$P2_12_12_1$ (19)
a [Å]	7.988(6)	10.069(2)	4.4843(12)	8.2414(7)
b [Å]	10.300(7)	9.296(2)	10.040(3)	8.9659(7)
c [Å]	14.389(11)	30.172(5)	17.221(5)	16.9866(15)
α [°]	90.646(10)	90	95.885(7)	90
β [°]	105.75(2)	90	93.589(9)	90
γ [°]	90.28(3)	90	99.415(16)	90
Volume [Å³]	1139.3(14)	2824.1(10)	758.4(3)	1255.17(18)
Z	4	8	2	4
Radiation	MoK_α (λ=0.71073 Å)	MoK_α (λ=0.71073 Å)	MoK_α (λ=0.71073 Å)	CuK_α (λ=1.54178 Å)
ρ_{calc} [gcm^{-3}]	1.342	1.300	1.342	1.293
μ [mm^{-1}]	0.094	0.083	0.089	0.724
F(000)	488	1168	324	520
2θ range [°]	2.94 to 56.76 (0.75 Å)	2.70 to 66.34 (0.65 Å)	4.14 to 80.93 (0.55 Å)	10.42 to 155.07 (0.79 Å)
Reflections collected	29964	165699	80207	65895
Independent reflections	5713 R_{int} = 0.0472 R_{sigma} = 0.0393	5400 R_{int} = 0.0853 R_{sigma} = 0.0274	9635 R_{int} = 0.0800 R_{sigma} = 0.0463	2669 R_{int} = 0.0576 R_{sigma} = 0.0146
Data / Restraints / Parameters	5713 / 0 / 309	5400 / 0 / 190	9635 / 0 / 209	2669 / 0 / 166
Final R indexes [I≥2σ(I)]	R_1 = 0.0474 wR_2 = 0.1115	R_1 = 0.0495 wR_2 = 0.1228	R_1 = 0.0482 wR_2 = 0.1312	R_1 = 0.0264 wR_2 = 0.0717
Final R indexes [all data]	R_1 = 0.0657 wR_2 = 0.1206	R_1 = 0.0652 wR_2 = 0.1333	R_1 = 0.0681 wR_2 = 0.1475	R_1 = 0.0265 wR_2 = 0.0718
Largest peak/hole [eÅ^{-3}]	0.32/−0.24	0.31/−0.26	0.49/−0.29	0.22/−0.14
Goodness-of-fit on F^2	1.043	1.101	1.044	1.062
Flack X parameter	-	-	-	-0.03(4)

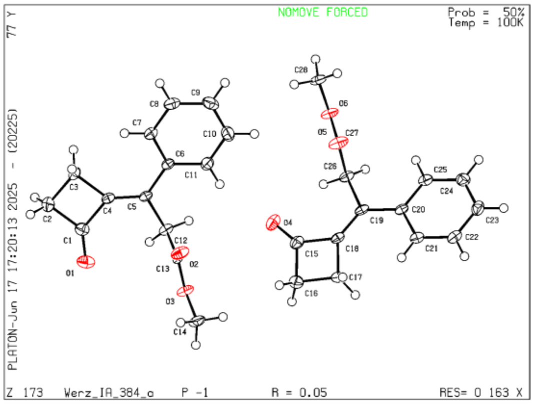

Fig. S1 Molecular structure of **3aa**. Ellipsoids correspond to 50% probability levels.

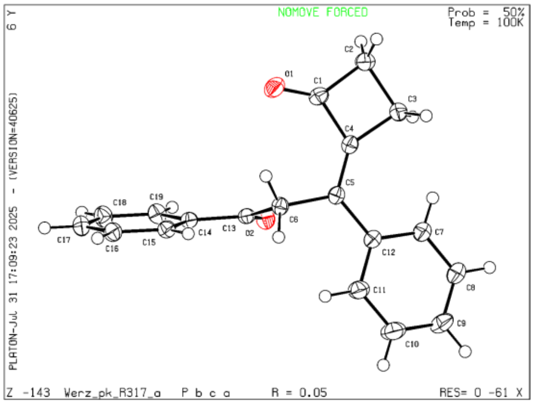

Fig. S2 Molecular structure of **3aj**. Ellipsoids correspond to 50% probability levels.

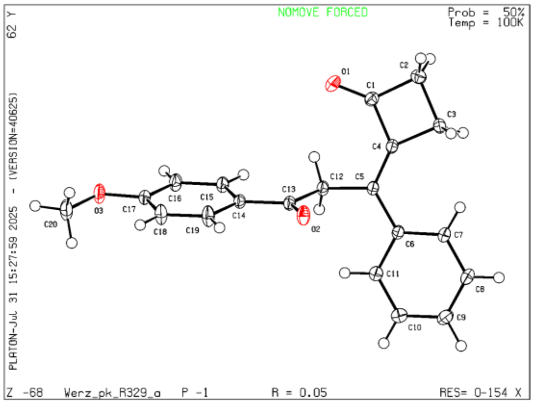

Fig. S3 Molecular structure of **3ak**. Ellipsoids correspond to 50% probability levels.

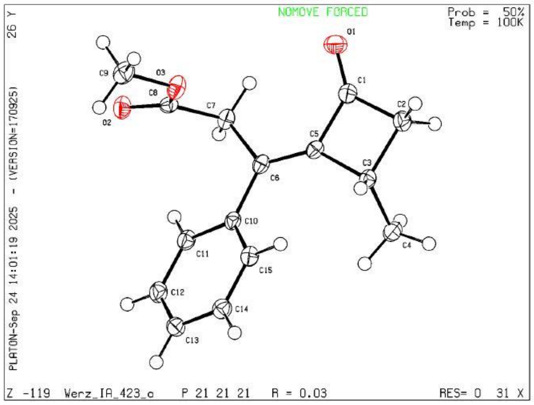

Fig. S4 Molecular structure of **3ba**. Ellipsoids correspond to 50% probability levels.

References

(1) Poteat, C. M.; Jang, Y.; Jung, M.; Johnson, J. D.; Williams, R. G.; Lindsay, V. N. G. Enantioselective Synthesis of Cyclopropanone Equivalents and Application to the Formation of Chiral β-Lactams. *Angew. Chem., Int. Ed.* **2020**, *59*, 18655−18661.

(2) (a) Vaitla, J.; Bayer, A.; Hopmann, K. H. Iron-Catalyzed Carbenoid-Transfer Reactions of Vinyl Sulfoxonium Ylides: An Experimental and Computational Study. *Angew. Chem. Int. Ed.* **2018**, *57*, 16180–16184. (b) Davas, D. S.; Gopalakrishnan, D. K.; Kumar, D.; Vaitla, J. Ru-Catalyzed Benzannulation of Vinyl Sulfoxonium Ylide with Electron-Deficient Alkynes and Alkenes. *Org. Lett.* **2022**, *24*, 8359–8363. (c) Gopalakrishnan, D. K.; Panigrahi, S.; Sen, R.; Vaitla, J. Ir(I)-Catalyzed Synthesis of Furanones from Vinyl Sulfoxonium Ylides. *Org. Lett.* **2023**, *25*, 1519–1524.

(3) Bruker, *SAINT*, *V8.41*, Bruker AXS Inc., Madison, Wisconsin, USA.

(4) Krause, L.; Herbst-Irmer, R.; Sheldrick, G. M.; Stalke, D. Comparison of Silver and Molybdenum Microfocus X-ray Sources for Single-Crystal Structure Determination. *J. Appl. Cryst.* **2015**, *48*, 3–10.

(5) Sheldrick, G. M. SHELXT - Integrated Space-Group and Crystal-Structure Determination. *Acta Cryst.* **2015**, *A71*, 3–8.

(6) Sheldrick, G. M. Crystal Structure Refinement With SHELXL. *Acta Cryst.* **2015**, *C71*, 3–8.

(7) Groom, C. R.; Bruno, I. J.; Lightfoot, M. P.; Ward, S. C. The Cambridge Structural Database. *Acta Cryst.* **2016**, *B72*, 171–179.

(8) D. Kratzert, *FinalCif*, *V143*, https://dkratzert.de/finalcif.html.
